# GWAS-Guided Compact SNP Panels Enable Breeding-Relevant Prediction of Bolting and Flowering Timing of Lettuce

**DOI:** 10.3390/plants15111621

**Published:** 2026-05-25

**Authors:** Kyung-San Son, Kyung-Man Kim, Daegwan Kim, Haying Youl Lee, Sung Yi Hong, So Hyun Kim, Suk-Woo Jang, Junhui Park, Tae-Sung Kim

**Affiliations:** 1 Department of Agricultural and Life Sciences, Korea National Open University, Seoul 03087, Republic of Korea; sson0116@knou.ac.kr (K.-S.S.); kalim229@gmail.com (K.-M.K.); lhy1559@hanmail.net (H.Y.L.); h353996@knou.ac.kr (S.Y.H.); rla0417@naver.com (S.H.K.); 2 DNACARE Co., Ltd., Seoul 06126, Republic of Korea; gardener@dnacare.co.kr; 3 Jeonbuk Agricultural Research & Extension Services, Iksan 54591, Republic of Korea; swjang63@naver.com; 4 Department of Statistics and Data Science, Korea National Open University, Seoul 03087, Republic of Korea; j22u22n22@knou.ac.kr

**Keywords:** lettuce, reproductive timing, GWAS, genotyping-by-target-sequencing (GBTS), SNP panel, genomic prediction

## Abstract

High temperatures accelerate bolting and shorten the vegetative phase, thereby reducing the marketable yield in lettuce(*Lactuca sativa* L.). Using the KNOU lettuce core collection (KLC; *n* = 288), which represents major horticultural types, we integrated genome-wide association studies (GWAS) with genotyping-by-target-sequencing (GBTS), a multiplex target amplicon sequencing approach, to develop compact SNP marker panels for breeding-relevant prediction of reproductive timing. The KLC was genotyped via genotyping-by-sequencing (GBS; 97,528 SNPs) and phenotyped across two spring-to-summer seasons to analyze cumulative temperature to bolting (CTTB) and cumulative temperature to anthesis (CTTA) under protected cultivation conditions, revealing broad variation and high heritability (H = 0.79 and 0.74, respectively). Multi-model GWAS consistently identified a major hotspot on chromosome 7 for both traits, whereas additional loci showed trait- and year-specific effects. A lead SNP on chromosome 7 was validated by KASP, confirming a consistent allelic effect across genetic backgrounds. GWAS-supported loci were converted into compact GBTS panels (CTTB-only, CTTA-only, and pooled), and their ability to predict genomic estimated breeding values (GEBVs) was evaluated via repeated 5-fold cross-validation. The pooled panel achieved the highest predictive performance for CTTB (up to R^2^ = 0.41 with random forest and R^2^ = 0.37 with RR-BLUP), outperforming the CTTB-only panel. In contrast, CTTA prediction was more moderate (up to R^2^ = 0.32). Overall, this GWAS-to-GBTS panel strategy provides a practical basis for low-cost, early selection of reproductive timing in lettuce breeding.

## 1. Introduction

Lettuce (*Lactuca sativa* L.) is an economically important vegetable valued at over $10 billion worldwide [1]. Cultivated lettuce is generally classified into several major horticultural types based on head architecture, including leaf, romaine, iceberg, and butterhead [2]. However, lettuce cultivars show substantial variation in texture, flavor, and color and provide nutritional value, including vitamin K, when consumed fresh [2]. In many temperate regions, low-cost greenhouse facilities enable commercial lettuce production during the cooler months, including winter. However, cultivation during the warmer months (spring to fall) remains challenging because lettuce is a cool-season crop, making it highly susceptible to heat stress, which severely impairs its growth and development [3].

Bolting is the developmental transition from a vegetative to a reproductive phase [4]. Long days and high temperatures promote differentiation of the shoot apical meristem into a floral meristem, leading to rapid stem elongation and flowering [5,6]. Consequently, lettuce grown under warmer conditions bolts earlier, thereby shortening the vegetative period and compromising yield potential [5,6]. Premature bolting further reduces marketability by impairing head formation, altering leaf shape, and increasing leaf bitterness [7,8]. These effects are likely to intensify with continued global warming [9].

Extensive studies in Arabidopsis thaliana have provided a conceptual framework for bolting and flowering in crops [3,4,10,11,12,13], which has guided functional studies in lettuce to dissect the underlying molecular mechanisms [14,15,16]. The availability of a high-quality lettuce reference genome [17] has further enabled natural-variation approaches, including QTL mapping and genome-wide association studies (GWAS) [18,19,20], as well as omics and functional analyses [5,6,21,22]. Together, these efforts have accelerated the dissection of regulatory networks controlling the vegetative-to-reproductive transition in lettuce. In particular, flowering integrators such as *FLOWERING LOCUS T* (*LsFT*) and *SUPPRESSOR OF OVEREXPRESSION OF CONSTANS 1* (*LsSOC1*), together with hormone-related regulators including LsGA3ox1, LsARF3, and the DELLA protein LsRGL1, have been identified as key components of heat-induced bolting pathways [14,16]. Collectively, these findings indicate that temperature, gibberellin, auxin, and DELLA-mediated signaling converge to promote bolting under high-temperature conditions [14,16]. However, this molecular knowledge alone does not readily translate into robust, breeding-ready marker sets that can effectively predict heat-induced bolting, a complex quantitative trait [23,24].

Previous QTL mapping studies have begun to bridge this gap by identifying loci associated with bolting and flowering traits [18,19]. Rosental et al. [18] reported *qFLT7.2* on chromosome 7 as a major locus under long-day (16 h) and high-temperature (35 °C) conditions, and *qBFr2.1* on chromosome 2, co-localizing with *LsFT*, as a complementary regulator across diverse environments. A separate study identified significant QTLs confined to a narrow 15.5–22.4 cM window on chromosome 7 [19], suggesting a genomic hotspot with pleiotropic effects on bolting, flowering time, and stem elongation.

Despite these advances, QTL mapping remains limited for broad breeding use because it is typically confined to biparental populations [25]. In contrast, GWAS captures natural variation across diverse germplasm panels and can identify major-effect loci in breeding-relevant backgrounds [26,27]. Even without fully capturing the genetic architecture of a complex trait, GWAS-derived SNPs may still be useful when assembled into marker panels for practical prediction using the well-established genomic prediction approach [28,29,30].

To this end, we used a genetically diverse lettuce core panel representing the major horticultural types, with particular emphasis on Korean leaf lettuce resources, to characterize population structure and quantitative variation in cumulative temperature to bolting (CTTB) and cumulative temperature to anthesis (CTTA) under warm conditions. We then applied multi-model GWAS, together with genotyping-by-target sequencing (GBTS), to identify and validate key loci associated with CTTB and CTTA. Based on these loci, we assembled trait-specific SNP marker panels and additionally constructed a pooled panel combining CTTB- and CTTA-associated SNPs into a single marker set. Finally, we developed and evaluated multiple genomic prediction models using these small SNP panels to assess whether they provide sufficient accuracy for predicting bolting tolerance in breeding lines. Overall, this study provides a practical, cost-effective SNP marker set to support GEBV-based selection for improved bolting tolerance in lettuce breeding via a genomic prediction framework.

## 2. Results

### 2.1. Population Structure and Relative Kinship of the KNOU Lettuce Core (KLC)

**Figure 1:**
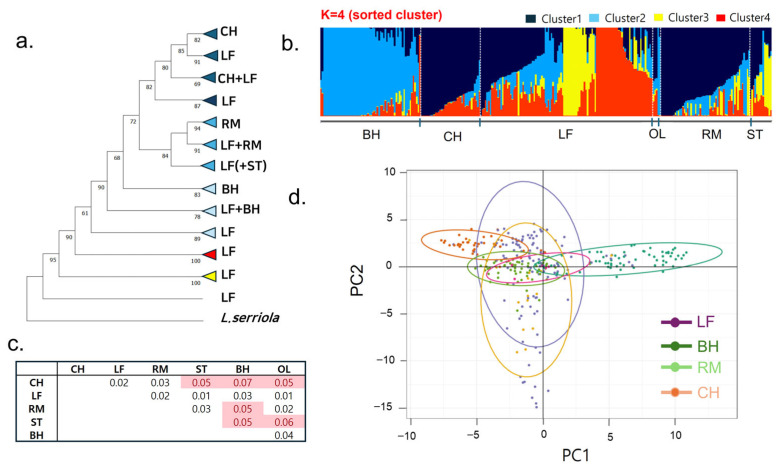
Genetic relationship and population structure among KNOU lettuce core (KLC) accessions. (**a**) Phylogenetic tree constructed using the Maximum Likelihood (ML) method, rooted with *Lactuca serriola* as the outgroup. Bootstrap values from 1000 iterations are displayed at each node. Branches representing the same lettuce type—including crisphead (CH), leaf (LF), romaine (RM), butterhead (BH), oakleaf (OL), and stem (ST)—are compressed for clarity. (**b**) Population structure analysis of KLC accessions. The bar plot illustrates the estimated membership proportions of individual accessions across four genetic clusters (K = 4), determined using the Evanno method. Each vertical bar represents an accession, with colors indicating the genomic proportion assigned to each cluster, grouped by lettuce type, with white dashed vertical lines indicating the boundaries between horticultural types. (**c**) Pairwise Fst matrix among lettuce types within KLC. Higher Fst values (>0.05), indicating greater genetic differentiation between types, are highlighted in red. (**d**) Principal component analysis (PCA) of KLC accessions. The scatter plot displays genetic variation among accessions, with colored ellipses representing the 95% confidence intervals for each lettuce type. All analyses were based on 3227 high-quality (HQ) SNPs.

To comprehensively analyze the population structure and genetic relationships among accessions in the KNOU Lettuce Core (KLC), we performed a series of analyses, including phylogenetic tree construction, STRUCTURE analysis, Fst calculation, and principal component analysis (PCA), based on 3227 high-quality (HQ) SNPs (Figure 1).

We first constructed a phylogenetic tree using the Maximum Likelihood (ML) method with 1000 bootstrap iterations, rooted with *L. serriola* as the outgroup, to provide a framework for the genealogy among the KLC accessions (Figure 1a). To enhance clarity, branches representing the same lettuce type were compressed, emphasizing the clustering patterns of crisphead (CH), butterhead (BH), romaine (RM), and leaf (LF) types, thereby illustrating their genetic relationships within the KLC (Figure S1).

The resulting tree showed partial clustering by lettuce type, with several subclades supported by moderate-to-high bootstrap values (Figure 1a). Although complete separation among horticultural types was not observed, BH occupied a relatively distinct branch, whereas CH and RM were each embedded in LF-associated mixed clades. LF was distributed across multiple clades, and LF-associated intermediate groups further reinforced the incomplete differentiation among horticultural types.

We next performed STRUCTURE analysis using the same SNP set to investigate population structure within the KLC (Figure 1b). The optimal number of clusters (K = 4) was determined using the Evanno method [22] (Figure S2). At K = 4, BH exhibited a distinct genetic profile dominated by Cluster 2, clearly distinguishing it from CH, RM, and ST. CH and RM were primarily associated with Cluster 1, although CH also contributed to Cluster 4. RM and ST, by contrast, exhibited varying degrees of admixture from other clusters. LF displayed the most heterogeneous ancestry pattern, with contributions from all four clusters, indicating broad genetic diversity and extensive admixture across horticultural types. OL also showed a complex ancestry pattern, but the small number of OL accessions in the KLC may not support a definitive interpretation. Together, the phylogenetic tree and STRUCTURE analyses indicate the relative distinctness of BH, the extensive admixture in LF, and incomplete differentiation among the remaining horticultural types (Figure 1a,b).

To further investigate genetic differentiation among lettuce types in the KLC, we calculated pairwise Fst values (Figure 1c). Overall, pairwise Fst values were low, ranging from 0.01 to 0.07, indicating limited but variable differentiation among horticultural types. LF showed consistently low differentiation from the other major lettuce types, with Fst values of 0.01 with ST, 0.02 with CH and RM, and 0.03 with BH. CH-RM and RM-ST comparisons were also relatively low (both Fst = 0.03). In contrast, BH showed the highest differentiation, particularly from CH (Fst = 0.07) and from RM and ST (Fst = 0.05 for both), supporting its relative distinctness. Overall, the Fst results complement the phylogenetic tree and STRUCTURE analyses by supporting the relative distinctness of BH, the low differentiation of LF from multiple horticultural types, and incomplete differentiation among the remaining horticultural types (Figure 1a–c).

To complement the phylogenetic, STRUCTURE, and Fst analyses, we performed principal component analysis (PCA) using the same SNP dataset (Figure 1d). PC1 and PC2 explained 45% and 35% of the total genetic variance, respectively. The PCA pattern was broadly consistent with the previous analyses. BH was clearly shifted along PC1 in the positive direction, indicating relative separation from the other lettuce types. In contrast, CH was displaced in the opposite direction, although partial overlap with RM persisted. RM occupied a more central position and did not form a fully separated cluster. In contrast, LF showed the broadest dispersion, particularly along PC2, consistent with a heterogeneous genetic background. Overall, the PCA supports the relative distinctness of BH, the broad dispersion of LF, and incomplete differentiation among the remaining horticultural types in the KLC (Figure 1d).

### 2.2. Phenotypic Variation in Bolting and Flowering Traits Within the KLC

**Figure 2:**
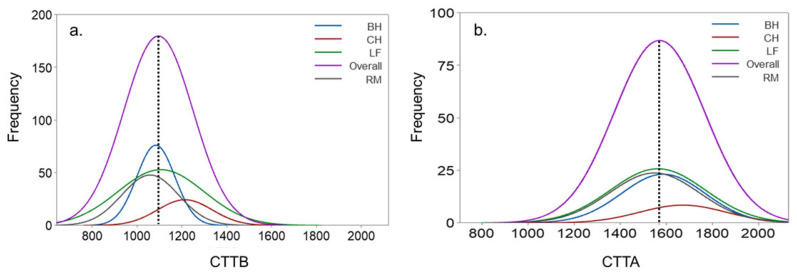
The distribution of cumulative temperature to bolting (CTTB) and cumulative temperature to anthesis (CTTA) among KLC accessions. (**a**) The frequency distribution of CTTB values across major lettuce types, including butterhead (BH), crisphead (CH), leaf (LF), and romaine (RM), as well as the overall distribution combining all KLC accessions. (**b**) The frequency distribution of CTTA values across the same lettuce types and the overall population. The dashed vertical line represents the mean value across the entire KLC population for each trait.

To characterize phenotypic variation in bolting and flowering traits within the KLC, we analyzed cumulative temperature to bolting (CTTB) and cumulative temperature to anthesis (CTTA) measured across two spring-to-summer greenhouse seasons (Figure 2, Figure S3 and S4, Table 1). Across all accessions, both traits showed continuous, approximately normal distributions, suggesting polygenic inheritance. Mean CTTB and CTTA were 1118.83 °C and 1603.62 °C, respectively, corresponding to an average difference of 484.79 °C between bolting and anthesis. Broad-sense heritability was high for both traits (H = 0.79 for CTTB and 0.74 for CTTA), and PCV exceeded GCV in both cases, indicating that environmental effects contributed to phenotypic variation, although genetic effects remained predominant.

Among horticultural types, CTTB differed significantly among all four groups (Figure 2a and Figure S3a, and Table 1). CH showed the highest mean CTTB (1225.52 °C), followed by LF (1131.37 °C), BH (1085.00 °C), and RM (1065.55 °C). LF had the highest GCV (14.00) and PCV (15.71), indicating the greatest within-type variability for bolting, whereas BH showed the lowest values (GCV = 4.57; PCV = 5.97), indicating relatively limited variation. Broad-sense heritability for CTTB was highest in LF and RM (both H = 0.79).

For CTTA, differences among types were less pronounced than for CTTB (Figure 2b and Figure S3b, and Table 1). CH again showed the highest mean CTTA (1682.54 °C), whereas BH (1613.49 °C) and LF (1600.14 °C) were intermediate, and RM showed the lowest mean (1564.52 °C). LF showed the highest GCV (8.24) and PCV (9.23) for CTTA, whereas CH showed the lowest values (GCV = 3.02; PCV = 4.76). CTTB and CTTA were positively associated (R-sq = 18.45%, *p* < 0.001; Figure S4), indicating that accessions with later bolting tended to reach anthesis later, although the relationship was moderate. Overall, CTTA showed lower GCV and PCV than CTTB, indicating lower relative variation at anthesis than at bolting.

### 2.3. Genome-Wide Association Study of Bolting and Flowering Traits in the KLC

**Figure 3:**
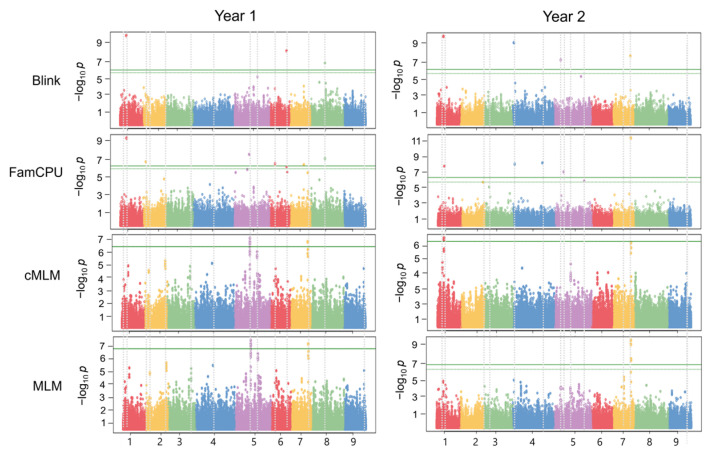
Manhattan plots of GWAS results for cumulative temperature to bolting (CTTB) across two years and four GWAS methods. Four GWAS methods—Blink, FamCPU, cMLM, and MLM—were used to identify associated loci across two years. The *x*-axis represents genomic positions across nine chromosomes, while the *y*-axis shows the −log10(*p*-value or *p*) of SNP associations. The solid green horizontal line indicates the significance threshold specific to each method. Dashed gray lines highlight regions containing candidate SNPs, either repeatedly exceeding the significance threshold or showing distinct patterns suggestive of association. In each Manhattan plot, different colors indicate SNPs located on different chromosomes.

Bolting and flowering are complex traits influenced by multiple genes and environmental factors. To identify SNPs associated with cumulative temperature to bolting (CTTB) and cumulative temperature to anthesis (CTTA), we performed GWAS using four complementary models: MLM, cMLM, FarmCPU, and BLINK. We evaluated 288 KLC accessions over two years, and GWAS was performed separately for each year using accession-level mean phenotypes calculated from the corresponding annual measurements. In the Manhattan plots, we focused primarily on SNPs exceeding the model-specific significance threshold and additionally noted recurrent suggestive signals detected across multiple years or models (Figure 3 and Figure S5).

For CTTB, recurrent association signals were mainly detected on chromosomes 1 and 7, with additional year-specific signals on several other chromosomes (Figure 3 and Table 2). Among these, Ch1_59238807 was the most robust locus, showing the highest significance in both years and being detected by all four GWAS models (Table 2). Three chromosome 7 SNPs, Ch7_160010057, Ch7_162215421, and Ch7_162466345, were also repeatedly detected across years and models, supporting chromosome 7 as another major associated region for bolting time (Table 2). Together, these results indicate that CTTB variation in the KLC is influenced by recurrent major loci on chromosomes 1 and 7, as well as by additional loci with smaller or less stable effects across years and models.

For CTTA, recurrent association signals were also concentrated on chromosomes 1 and 7 (Figure S5). The chromosome 1 signal was especially pronounced in Year 2, whereas chromosome 7 showed repeated signals across several models and years (Figure S5 and Table S1). Compared with CTTB, CTTA showed fewer recurrent peaks above the significance threshold, consistent with the lower GCV and PCV estimated for CTTA (Table 1 and Table S1, Figure S5). Overall, these results indicate that CTTB and CTTA share part of their genetic architecture, particularly association signals on chromosomes 1 and 7, while differing in the strength and stability of individual loci across years and models.

### 2.4. Phenotypic Validation of Trait-Associated SNPs

**Figure 4:**
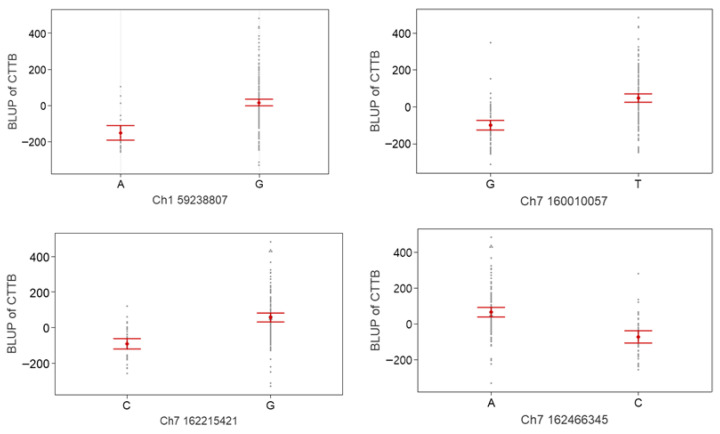
The marker effects of significant SNPs associated with the cumulative temperature to bolting (CTTB) across two years. Interval plots display the BLUP (Best Linear Unbiased Prediction) estimates of CTTB for four SNPs (Ch1: 59238807, Ch7: 160010057, Ch7: 162215421, and Ch7: 162466345) consistently identified in both Year 1 and Year 2. Each plot illustrates the effect of alleles at the corresponding SNP loci. Red points represent the mean BLUP values for each allele group, with horizontal red lines indicating the 95% confidence intervals. Individual black dots depict the BLUP values for individual accessions. These SNPs were selected as representative examples; the full results are shown in Figures S6 and S7.

To validate the phenotypic effects of recurrent GWAS loci, we compared BLUP values among allelic classes for selected SNPs associated with CTTB and CTTA. For CTTB, four loci detected in both years, Ch1_59238807, Ch7_160010057, Ch7_162215421, and Ch7_162466345, showed clear allele-group separation in BLUP interval plots, supporting consistent phenotypic effects across years (Figure 4). Additional CTTB-associated loci also showed allele-group differences, although several were detected in only one year, suggesting year-dependent effects and/or model-specific sensitivity (Figures S6 and S7).

**Figure 5:**
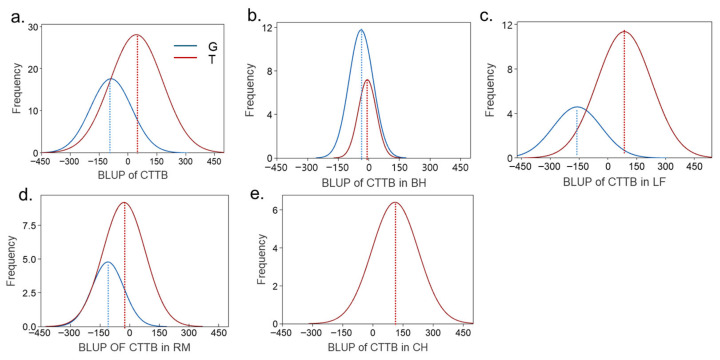
The KASP-validated allelic effect on cumulative temperature to bolting (CTTB) across lettuce types. Frequency plots depict the normalized distribution of BLUP (Best Linear Unbiased Prediction) values for CTTB, based on alleles (G and T) at SNP Ch7:160010057, across the major lettuce types within the KLC. (**a**) The overall CTTB distribution for all KLC accessions. (**b**–**e**) CTTB distributions for individual lettuce types: butterhead (BH), leaf (LF), romaine (RM), and crisphead (CH), respectively. Blue curves and dotted lines represent the G allele, while red curves and dotted lines represent the T allele. Dotted vertical lines denote the mean BLUP values for each allele group.

To further validate a recurrent locus, Ch7_160010057 was re-genotyped using KASP. The allelic contrast observed in the GBS data was reproduced, with the T allele associated with higher CTTB BLUP values than the G allele (Figure S8a,b). When examined by lettuce type, the direction of effect remained consistent. Still, its magnitude differed among types, appearing largest in LF, moderate in RM, smaller in BH, and not estimable in CH because the T allele was nearly fixed (Figure 5a–e). These results support a robust phenotypic effect at Ch7_160010057 in the horticultural types where the locus segregates and suggest that allele-frequency differences, together with genetic background, contribute to between-type differences in CTTB.

Applying the same validation framework to CTTA revealed broadly similar patterns. Several CTTA-associated loci overlapped with CTTB-associated loci, whereas others appeared to be trait-specific. Year-stratified interval plots showed clear allele-group separation for the relevant SNPs in each year (Figures S9 and S10). A KASP assay at Ch7_160010057 likewise reproduced the T > G contrast for CTTA (Figure S11), and recurrent CTTA loci showed consistent allelic effects in BLUP-based comparisons (Figure S12). Type-wise CTTA BLUP density curves also paralleled the CTTB pattern, with consistent effect direction but varying magnitudes among types; within-type comparisons were not possible in CH because the T allele was nearly fixed (Figure S13). Overall, these results support the phenotypic relevance of the validated loci for both CTTB and CTTA and suggest that differences in allele frequency and genetic background may modulate their effects.

### 2.5. Developing Predictive Models for CTTB and CTTA Using the Validated Candidate SNPs

Guided by the polygenic, environment-sensitive, yet highly heritable nature of CTTB and CTTA, we combined the validated loci into a multi-locus predictive framework (Figure S14). We first assessed trait-specific prediction using CTTB- and CTTA-associated SNPs for their respective targets (Figures S15 and S16). For each trait, full-data fits between observed deregressed BLUPs (DRPs) and predicted GEBVs were used as an exploratory baseline before model performance was further evaluated by repeated five-fold cross-validation.

Model-observation concordance varied across algorithms (Figure S15). The linear baselines (FEM, RR-BLUP, LASSO, and EN0.5) showed very similar performance, each explaining about 38% of the variance, with S (RMSE) values of 116.5–116.6. RKHS improved the fit relative to the linear models (R^2^ = 44.2%, S = 110.8), whereas RF performed best, with the highest explained variance and the lowest error (R^2^ = 51.9%, S = 102.8). All models showed modest miscalibration, with intercepts ranging from 13.7 to 31.1 and slopes from 1.09 to 1.23, indicating underestimation in the upper tail and overestimation in the lower tail (Figure S15). We therefore applied model-wise linear recalibration and derived approximate 95% prediction intervals (Table S2). After recalibration, the overall ranking remained unchanged: RF yielded the narrowest interval widths, followed by RKHS, whereas the linear models remained interpretable baseline approaches (Table S2).

To verify that the in-sample patterns were not artifacts of resubstitution, we next evaluated CTTB prediction from the validated CTTB-SNP panel via five-fold cross-validation (5 folds × 5 repeats = 25 runs) on the pooled two-year dataset (Table S3). Prediction accuracy was highly similar across models, with mean R^2^ values of approximately 0.27 and mean Pearson correlations of 0.49–0.50. RF showed the lowest mean RMSE (122.03), whereas the linear baselines (FEM, RR-BLUP, LASSO, and EN0.5) also performed similarly, with mean RMSE values ranging from 123.50 to 123.93. RKHS showed comparable mean R^2^ and mean correlation. Still, it had the highest mean RMSE (124.83) and extreme slope instability across runs (mean slope = 338.13, SD = 1683.91), indicating poor calibration robustness in cross-validation (Table S3). These results suggest that RF provided the strongest overall cross-validation performance, whereas the linear models remained competitive and more stable in calibration.

Parallel analyses for CTTA showed a similar pattern to CTTB, with the non-linear models outperforming the linear baselines. The linear baseline models clustered closely, with R^2^ values of 31.9–33.4% and S values of 109.9–111.2 (e.g., RR-BLUP, R^2^ = 33.4%, S = 109.9; FEM, R^2^ = 32.5%, S = 110.6; Table S4). RF and RKHS both improved model performance relative to the linear baselines, but in different ways: RF achieved the highest explained variance (R^2^ = 51.2%), whereas RKHS achieved the lowest error (S = 95.1). Both models also showed slopes close to unity (1.09 and 1.07, respectively) and small positive intercepts (22.7 and 23.9; Table S4). Approximate 95% prediction-interval half-widths were narrowest for RKHS and RF (178 and 181, respectively), compared with 201–209 for the linear baseline models (Table S4).

In five-fold cross-validation on the pooled two-year dataset, CTTA only partly recapitulated the full-fit pattern (Table S5). RF performed best overall (mean R^2^ = 0.31, mean r = 0.54, mean RMSE = 117.07), whereas the linear baselines clustered closely (mean R^2^ = 0.23–0.24; mean RMSE = 119.88–120.52) and remained competitive as stable, interpretable alternatives. By contrast, RKHS did not improve practical cross-validated performance.

Taken together, these results suggest that recalibrated RR-BLUP provides a practical default model for routine prediction of both CTTB and CTTA, given its stable calibration, simplicity, and competitive predictive performance. RF may serve as a useful non-linear alternative for operational screening.

### 2.6. Assessing Cross-Trait Pooling of Previously Validated SNPs for Predicting CTTB and CTTA

**Figure 6:**
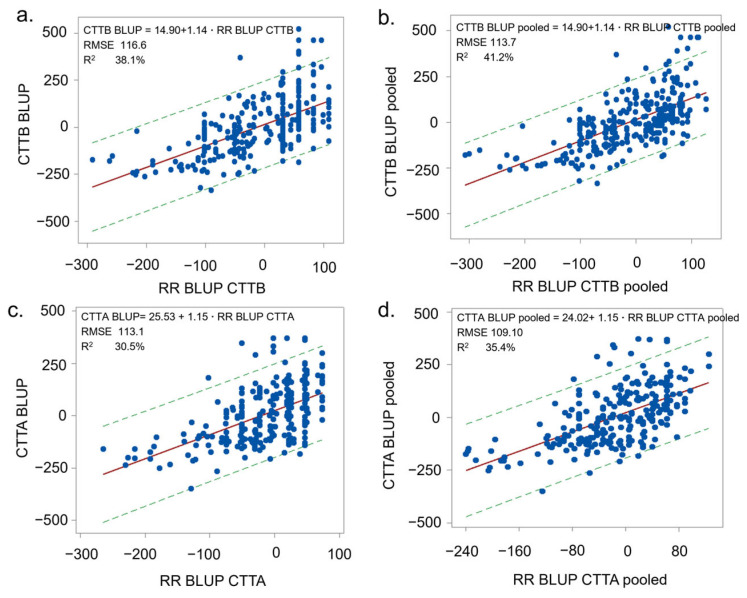
Full-data RR-BLUP fits comparing trait-specific versus pooled SNP panels for CTTB and CTTA. (**a**) CTTB trait-specific. (**b**) CTTB pooled. (**c**) CTTA trait-specific. (**d**) CTTA pooled. The pooled set combines the validated CTTB- and CTTA-associated SNPs into a single panel. The *x*-axis shows RR-BLUP predictions (genomic estimated breeding values, GEBVs), and the *y*-axis shows observed deregressed BLUPs (DRPs) for each accession. Dashed green lines indicate 95% prediction intervals, and the red line illustrates the ordinary least-squares fit with no recalibration.

Building on the trait-specific results, and given that CTTB and CTTA are biologically linked yet share only part of their validated signal, we next tested whether additional cross-trait information could be captured by combining the validated CTTB- and CTTA-associated SNPs into a single pooled panel. To visualize the effect of pooling, we compared pooled and trait-specific full-data RR-BLUP fits for CTTB and CTTA (Figure 6). In both traits, the pooled RR-BLUP fits showed slightly tighter dispersion and narrower approximate 95% prediction intervals around the regression line. We therefore evaluated pooled-panel performance more formally using five-fold cross-validation (Table 3).

To visualize the effect of pooling, we compared pooled and trait-specific full-data RR-BLUP fits for CTTB and CTTA (Figure 6). In both traits, the pooled fits showed slightly tighter dispersion and narrower approximate 95% prediction intervals around the regression line. Motivated by this visual pattern, we next evaluated the performance of the pooled panel more formally using five-fold cross-validation (Table 3).

Relative to the trait-specific baselines (Table S3), the pooled panel improved cross-validated prediction of CTTB across all models. RF achieved the highest point accuracy (R^2^ = 0.41 ± 0.10, r = 0.63 ± 0.08, RMSE = 114.50 ± 13.37, *b* = 1.16 ± 0.22), while RR-BLUP was competitive with lower variability and stable calibration (R^2^= 0.37 ± 0.08, *r* = 0.60 ± 0.07, RMSE = 119.23 ± 11.94, *b* = 1.14 ± 0.26) (Table 3). Linear baselines clustered just below RR-BLUP (R^2^ = 0.36 ± 0.08, RMSE = 120 ± 12), and RKHS lay between RF and the linear group (R^2^ = 0.39 ± 0.09, RMSE = 118.48 ± 12.97) but with a steeper slope (*b* = 1.25 ± 0.27) (Table 3).

For CTTA, pooling also improved cross-validated prediction relative to the trait-specific panel (Table S5), although the gains were more modest than for CTTB. RF again performed best overall (R^2^ = 0.35 ± 0.12, r = 0.59 ± 0.10), with the RMSE essentially unchanged (110.00 ± 14.06, *b* = 1.08 ± 0.22). RR-BLUP had the strongest linear baseline (R^2^ = 0.29 ± 0.11, r = 0.52 ± 0.12, RMSE = 116.09 ± 14.02, *b* = 1.06 ± 0.29), and FEM, LASSO, and EN (*α* = 0.5) also improved modestly under pooling. RKHS showed substantial recovery relative to the trait-specific panel, although RF remained superior overall (Table 3).

Pooling improved CTTB prediction by expanding the marker set and strengthening cross-trait signals, while leaving the relative rankings of models largely unchanged (Figure 6a,b, and Table 3). For CTTA, pooling also improved prediction, although the gains were smaller and more model-dependent (Figure 6c,d, and Table 3). Under the pooled panel, recalibrated RR-BLUP remained the most practical default for routine deployment, whereas RF remained the preferred non-linear alternative for operational screening.

## 3. Discussion

Warm-season bolting limits lettuce production by accelerating the vegetative-to-reproductive transition [31,32]. By translating trait-relevant signals into low-cost marker panels and demonstrating their predictive value with robust prediction models, we provide a breeding-oriented framework that supports practical selection for bolting tolerance under warm greenhouse conditions.

### 3.1. Population Structure Supports KLC as a Mapping Panel and a Breeding Resource

The KNOU lettuce core collection (KLC) was originally assembled as a breeding-oriented diversity panel to connect Korean leaf lettuce germplasm with globally diverse lettuce types. Of the 288 accessions, 186 were selected from the USDA lettuce germplasm collection, whereas 102 were domestic elite varieties mainly representing Korean leaf lettuce breeding materials. This composition was designed to combine broad genetic diversity with breeding-relevant Korean germplasm and support reciprocal trait improvement between Korean leaf lettuce and other lettuce types. In particular, the panel was intended to facilitate the incorporation of desirable heading- or romaine-type attributes, such as texture, uniformity, and market quality, into Korean leaf lettuce while also enabling the deployment of leaf-lettuce-associated traits into lettuce backgrounds familiar to international consumers.

Genome-wide analyses of 3227 high-quality SNPs showed that the KLC is structured in a type-dependent manner while retaining cross-type overlap. Across the maximum-likelihood phylogeny, STRUCTURE (K = 4), pairwise FST, and PCA, butterhead (BH) was consistently the most differentiated group. Crisphead (CH) and romaine (RM) shared partial ancestry and showed relatively low differentiation, although their separation was not complete. Leaf lettuce (LF), by contrast, displayed the broadest multi-component ancestry pattern and the lowest differentiation from other types, particularly RM and ST. In PCA, BH and CH were separated mainly along PC1, whereas LF remained comparatively central and broadly dispersed along PC2.

The relative positioning of individual non-leaf types may vary across panels depending on sampling balance and germplasm composition. However, the LF bridging pattern appears to be recurrent rather than incidental, as recent core-collection studies have also reported substantial admixture within LF [33]. These observations suggest that LF harbors broad segregating diversity while remaining genetically connected to the broader cultivated gene pool.

Because the KLC was not constructed as an origin-balanced global diversity panel, the observed structure was not interpreted primarily in terms of geographic or passport origin. Rather, it was more clearly associated with horticultural type and breeding background, as reflected by the relative distinctness of BH, the partial overlap between CH and RM, and the broad admixture pattern of LF. This interpretation is also consistent with the composition of the KLC, which includes both USDA-distributed germplasm and domestic elite Korean leaf lettuce varieties.

This population structure has two major implications for downstream analyses. First, because type-dependent stratification is substantial, association tests and genomic prediction models consider kinship and population structure to reduce confounding driven by type differences. Second, because LF shows the strongest cross-type overlap, the KLC provides a suitable framework for detecting alleles whose effects can be observed across multiple type backgrounds and for supporting cross-type validation and deployment.

Accordingly, the leaf-centered composition of the KLC reflects both the Korean consumption context and the intended breeding use of the panel, since leaf lettuce remains an important lettuce type in Korea, where salad consumption is gradually increasing [34,35]. Therefore, a panel connecting leaf lettuce with other lettuce types can provide a valuable genetic resource for cultivar development in both domestic and international markets. Consistent with this original purpose, the KLC has value beyond the present GWAS and prediction study, particularly as a reusable pre-breeding resource for future lettuce improvement.

### 3.2. Quantitative Genetic Properties Suggest Shared Biology but Partly Distinct Genetic Control of CTTB and CTTA

Across two spring-to-summer evaluations under non-temperature-controlled, protected-soil-bed greenhouse conditions, cumulative temperature to bolting (CTTB) and cumulative temperature to anthesis (CTTA) showed broad, continuous, near-normal variation in the KLC (Figure 2 and Figure S3). The population means were 1118.83 °C for CTTB and 1603.62 °C for CTTA (Table 1), corresponding to an average thermal-time interval of 484.79 °C from bolting to anthesis. Both traits showed high broad-sense heritability (H = 0.79 for CTTB; H = 0.74 for CTTA), while PCV exceeded GCV for both traits (CTTB: 12.96 vs. 11.53; CTTA: 8.13 vs. 7.02; Table 1). This indicates that environmental variation contributed to the observed phenotypic variation, but that genetic effects remained predominant and sufficiently stable for genetic analysis and breeding-oriented interpretation.

The robustness of this phenotypic dataset derives from both its experimental design and trait definition. The KLC was evaluated over two spring-to-summer seasons using replicated RCBD trials, and accession-level values were calculated from multiple plants within blocks in each season, as described in Section 4.1. In addition, CTTB and CTTA were expressed as cumulative thermal time rather than calendar days, allowing developmental timing to be interpreted in relation to seasonal temperature exposure. Under non-temperature-controlled, protected-soil-bed greenhouse conditions, year-to-year differences in temperature trajectories, solar radiation, humidity, ventilation, and soil-bed microenvironments can still influence developmental timing. Despite these environmental components, the high broad-sense heritability values for both traits indicate that genetic effects remained predominant, while the broad phenotypic variation and type-wise differentiation further support the breeding relevance of the dataset (Figure 2 and Figure S3; Table 1). Therefore, the CTTB and CTTA phenotypes provided a suitable basis for GWAS, BLUP-based allele-effect validation, and genomic prediction for early selection of delayed bolting (Figure 3, Figure 4, Figure 5 and Figure 6 and Figures S5–S13; Table 2 and Table 3).

CTTB and CTTA are developmentally coupled because anthesis follows bolting, and their significant positive correlation supports this shared biology (R^2^ = 18.45%, *p* < 0.001; Figure S4). However, the moderate strength of this relationship indicates that the two traits are not fully interchangeable. CTTA showed lower relative dispersion than CTTB, as reflected by lower GCV and PCV values (Table 1). This pattern is consistent with tighter constraints on anthesis timing and/or scale effects expected for a downstream trait defined after bolting. Therefore, it is more conservative to treat the apparent asymmetry—broader variation in bolting onset than in anthesis timing—as a panel-level observation that motivates genetic dissection, rather than as definitive evidence of independent regulation. Thus, it is plausible that CTTB and CTTA share part of their upstream developmental control while retaining trait-specific components, which may explain their moderate correlation and distinct variation patterns.

Type-wise summaries provided a structured view of this variation (Figure 2 and Figure S3, and Table 1). CH had the latest mean values for both traits (CTTB 1225.52 °C; CTTA 1682.54 °C), whereas RM was the earliest (CTTB 1065.55 °C; CTTA 1564.52 °C). Differences among types were more pronounced for CTTB than for CTTA, consistent with the narrower relative spread observed for CTTA at the population level. Importantly, LF combined intermediate means (CTTB 1131.37 °C; CTTA 1600.14 °C) with strong within-type segregation, especially for bolting (CTTB GCV 14.00; H = 0.79), and substantial variation for CTTA (GCV 8.24; H = 0.80; Table 1). This indicates that LF contains useful segregating variation for reproductive timing while retaining overlap with other horticultural types.

Overall, the type-wise phenotypic patterns indicate that CTTB and CTTA capture both between-type differences and within-type variation relevant to selection. In particular, the substantial within-type variation observed in LF suggests that delayed-bolting selection can be pursued within leaf lettuce germplasm as well as across broader horticultural backgrounds.

### 3.3. Chromosome 7 Harbors a Developmental Hotspot That Consistently Delays Bolting and Flowering, but Additional Loci Explain Trait- and Environment-Dependent Variation

Comparative studies in lettuce indicate that bolting and flowering time are polygenic traits, with QTLs and association signals distributed across all chromosomes and recurrent hotspots, particularly on chromosomes 2 and 7 [18,31,36]. In this context, our KLC analyses indicate that Chr7 represents a reproducible hotspot for developmental timing, whereas additional loci contribute in trait- and environment-dependent ways. In the KLC, Chr7 signals were repeatedly recovered for CTTB across years, and GWAS models (Figure 3; Table 2), and Chr7 enrichment was also observed for CTTA (Figure S5), suggesting that this region may harbor component(s) associated with developmental timing and may therefore contribute to a shared upstream developmental process.

Despite this repeatability, causal resolution within the Chr7 interval remains limited. The broad Chr7 peak could reflect a single causal variant tagged by multiple SNPs, several linked variants within the same interval, or both. Accordingly, it is more defensible to interpret Chr7 as a robust association region than as a single resolved causal site. Fine mapping, haplotype dissection, and functional validation will therefore be needed to resolve the causal basis of this hotspot.

Independent lettuce studies further support the biological plausibility of this region. Rosental et al. evaluated a biparental lettuce RIL population under combinations of photoperiod and temperature and identified qFLT7.2 on Chr7 as a major QTL for bolting and flowering time [18]. Whereas that study established the importance of the Chr7 region in a specific two-parent genetic background, the present KLC analysis extends its relevance to a broader diversity panel representing multiple horticultural types and breeding backgrounds. The repeated detection of Chr7 signals across these different experimental frameworks suggests that this region contributes to a broad developmental-timing axis in lettuce. However, this does not necessarily imply that the same causal variant or haplotype explains all Chr7-associated variation; rather, the Chr7 hotspot should be viewed as a shared but still unresolved developmental-timing region.

This interpretation is consistent with other recent lettuce studies. Tripodi et al. detected a strong bolting-time association near 164 Mb on Chr7 [36], and Anton-Sales et al. showed that a truncating PHYC allele delays bolting and flowering while decelerating the circadian clock [37]. Together, these studies point to light- and circadian-regulation as a plausible mechanistic basis for recurrent Chr7 signals in lettuce [18,36,37]. However, because the broad Chr7 region may contain multiple candidate genes, regulatory variants, and possible interactions among causal components, PHYC should be considered a biologically plausible candidate rather than a confirmed causal gene. In particular, its relationship to the Chr7 hotspot identified in the present study should be tested in future work, especially if local LD or haplotype analysis indicates that the association signal extends into the PHYC region.

Although GBS enabled effective genome-wide scanning and the development of practical SNP panels, additional sequence-level resolution will be required to identify the causal gene(s) or variant(s) within the broad Chr7 hotspot. The observed association may reflect a causal variant tagged by nearby SNPs, multiple linked variants, regulatory variation affecting one or more candidate genes, or interactions among causal genes or regulatory elements within the region. In this context, the KASP validation of Ch7:160010057 provided important technical support for the robustness of the lead marker by resolving the GBS missing genotype class and reproducing the allelic effect for both CTTB and CTTA. Thus, the Chr7 signal should be interpreted as a robust but unresolved developmental-timing hotspot.

Together, these results provide a foundation for future fine-scale genetic and functional studies of the Chr7 hotspot. Available lettuce resequencing resources [33] can guide GBTS-assisted map-based cloning of this region by refining candidate SNPs, constructing local haplotypes, and prioritizing variants associated with developmental timing. After the Chr7 interval and candidate haplotypes are refined, controlled-environment transcriptomic and proteomic profiling of materials carrying contrasting favorable and unfavorable haplotypes may help identify causal components within the hotspot and clarify how they are connected to downstream developmental pathways, including flowering integrators and hormone-related regulators. Recent single-cell and spatial transcriptomic approaches in crop and shoot-apex studies further suggest that such pathways could be resolved at cell-type or spatial-domain resolution during the vegetative-to-reproductive transition [38]. Subsequent functional validation of the prioritized candidates will further clarify how the Chr7 hotspot contributes to bolting and flowering time in lettuce.

Type-stratified density profiles further indicate that the direction of effect is conserved in BH, LF, and RM, where the locus segregates. In contrast, within-type contrasts are limited in CH because the late-development allele is nearly fixed. The relatively balanced allele frequency of the lead Chr7 marker at the panel level, together with the conserved direction of allelic effect across segregating horticultural backgrounds, supports the interpretation that this Chr7 signal is not simply a type-specific association. Instead, it likely represents a broadly relevant developmental-timing region that can contribute to delayed bolting and flowering across genetic backgrounds. However, the near fixation of the late-development allele in CH is unlikely to reflect random sampling alone and suggests an allele-frequency shift associated with the CH background. Whether this pattern resulted from selection for delayed bolting, founder effects, or sampling composition remains unresolved and should be tested with a larger, more balanced CH germplasm set.

Beyond Chr7, our results also indicate additional loci whose detectability varies by trait and year. For CTTB, several non-Chr7 loci showed clear allelic contrasts in the specific year in which they were detected (Figures S6 and S7), whereas for CTTA, additional signals, particularly on Chr1, became more prominent in specific years (Figures S9 and S10). These patterns may reflect small modifier effects detectable only for a particular trait, year, or genetic background. However, some trait- or year-specific signals may also reflect statistical fluctuations or false-positive associations. Because CTTB and CTTA represent sequential, partially shared developmental processes, loci that are weakly detected for CTTB in a given year may still be captured by CTTA-associated signals and contribute to pooled-panel prediction. The improved pooled-panel performance may therefore reflect the recovery of shared developmental-timing information that was partially masked in single-trait or single-year GWAS. Together, these results suggest a two-layer genetic architecture of developmental timing in the KLC, comprising a stable Chr7 region shared by CTTB and CTTA, as well as additional putative modifier loci with trait- and environment-dependent effects.

### 3.4. Prediction with Small SNP Panels Is Feasible in Future Lettuce Breeding

GWAS identifies marker-trait associations, but single-locus signals are rarely sufficient on their own for routine selection of quantitative traits. In this context, marker-assisted selection (MAS) and genomic prediction-based selection are not interchangeable [39,40,41]. MAS is useful for tracking major validated loci, such as the recurrent Chr7 signal. Still, even when multiple target markers are used, selection is often based on allele presence or on simple multi-marker combinations rather than on phenotype-informed estimation of their joint effects [40]. This limits its ability to capture the unequal, background-dependent contributions of multiple loci to quantitative variation [40]. By contrast, genomic prediction-based selection integrates the joint effects of multiple validated SNPs into per-line genomic estimated breeding values (GEBVs), thereby supporting more refined line prioritization in the breeding program [39,41]. In the present study, this framework was evaluated using compact, validated SNP panels for CTTB and CTTA via repeated cross-validation on a pooled two-year dataset, along with calibration checks to improve interpretability for breeding use.

GBTS offers a practical implementation route because predefined loci can be multiplexed into a single targeted panel via a standard PCR-based workflow, followed by next-generation sequencing and downstream genotype calling, allowing multiple loci to be assayed simultaneously rather than individually. The agreement between GBTS- and KASP-based genotypes at the validated lead locus further supports the reproducibility of targeted marker calls. It indicates that such panels can be incorporated into routine breeding workflows (Figure S14). In this sense, the present panel is better viewed not as a conventional MAS tool based on a single or a few diagnostic loci, but as a compact prediction platform that translates validated GWAS signals into breeding values usable in routine selection.

Using these compact panels, prediction was repeatable for both traits under cross-validation, although accuracy remained moderate. For the trait-specific panels, mean cross-validation R^2^ was about 0.27 for CTTB across models and about 0.23–0.31 for CTTA, with RR-BLUP remaining competitive and stable among the linear baselines. Accordingly, the current panels appear best suited to early-stage culling and preliminary line prioritization rather than to direct replacement of replicated phenotypic testing. Pooling previously validated CTTB- and CTTA-associated SNPs showed that modest panel expansion can recover additional usable signals. For CTTB, pooled-panel performance improved clearly, reaching cross-validated R^2^ values of 0.41 with RF and 0.37 with RR-BLUP (Figure 6a,b). For CTTA, pooling also improved prediction, although the gains were smaller than for CTTB (Figure 6c,d; Table 3). This pattern is consistent with partial sharing of developmental timing signals between the two traits, alongside trait-specific components and environmental sensitivity, suggesting that the pooled panel may recover predictive information that is partially masked in single-trait or single-year GWAS.

From a deployment perspective, recalibrated RR-BLUP remains a practical default because of its stable calibration, simplicity, and competitive performance. In contrast, RF provides a useful non-linear alternative when departures from purely additive behavior are likely to matter. These results show that a small, GWAS-validated SNP panel can provide repeatable ranking information relevant to breeding decisions, even when the utility of individual lead markers varies among horticultural types. For example, the lead Chr7 allele was nearly fixed in CH, limiting its within-type discriminatory power, whereas the pooled panel could still capture additional multi-locus information. Importantly, GBTS panels can be flexibly modified and expanded to include newly validated target loci, so the present panel should be viewed as an updateable predictive platform rather than a fixed marker set. Further multi-environment validation and incremental incorporation of additional validated loci will be important for improving predictive accuracy, reliability, and transferability before routine use in warm-season lettuce breeding.

## 4. Materials and Methods

### 4.1. Plant Materials and Phenotypic Evaluation of Bolting and Flowering Traits

A KNOU (Korea National Open University) lettuce core collection (KLC) comprising 288 accessions was assembled for this study. The panel covered diverse horticultural types, including butterhead (BH; *n* = 64, 22%), romaine (RM; *n* = 57, 20%), crisphead (CH; *n* = 39, 14%), leaf type (LF; *n* = 109, 38%), stem lettuce (ST; *n* = 14, 5%), and oakleaf (OL; *n* = 5, 2%). Detailed passport information and accession sources are provided in Supplementary Table S6.

Phenotyping was conducted in 2018 and 2019 during spring-to-summer seasons under non-temperature-controlled protected soil-bed greenhouse conditions. In both years, seeds were sown, and seedlings were raised under the same germination and nursery management conditions before transplanting. Seedlings were transplanted into greenhouse soil beds at 20 × 20 cm spacing in early May 2018 and late April 2019. Each year, accessions were evaluated in a randomized complete block design (RCBD) with two blocks and four plants per accession per block. Standard greenhouse management practices were applied throughout the cultivation period, and accession-level trait values were calculated by averaging replicate plants within each block and season.

Bolting-related developmental timing was quantified as cumulative temperature to bolting (CTTB). Based on observations that visible stem elongation typically occurred approximately 10 days after flower-bud differentiation under our conditions, the bolting date was operationally defined as the date of first visible flower-bud emergence (bud differentiation stage) and used as a proxy for bolting. Thermal time was calculated from daily mean greenhouse air temperature using a base temperature of 5 °C (daily value = T_mean − 5 °C). CTTB for each plant was obtained by summing daily thermal time from sowing to the defined bolting date.

Flowering time was quantified as cumulative temperature to anthesis (CTTA). For each accession, anthesis was defined as the date when 50% of plants had opened their first flower. CTTA was calculated by summing daily thermal time from sowing to the anthesis date using the same base temperature and calculation procedure as for CTTB. Thermal time from bolting to anthesis was derived as CTTA − CTTB when required. Quantitative-genetic parameters for CTTB and CTTA (mean, SE, GCV, PCV, and broad-sense heritability) were estimated following Kim et al. [42].

### 4.2. SNP Calling and Quality Control for the KLC Panel

Genome profiling of the KLC was conducted using genotyping-by-sequencing (GBS) with two restriction-enzyme library systems (ApeKI and PstI–MspI). Sequencing reads were processed by (i) unique dual-index (UDI) trimming using Cutadapt (v4.4) and (ii) quality trimming using Trimmomatic (v0.39). Demultiplexing was performed using the STACKS process_shortreads module (v2.61). Quality-filtered reads were mapped to the lettuce reference genome using BWA-MEM (v0.7.17), followed by variant calling using GATK HaplotypeCaller (v4.2). SNP filtering was performed using VCFtools (v0.1.16).

Quality control filtering was applied to obtain SNP sets tailored for downstream analyses. SNPs were retained only when supported by a minimum read depth > 5 and genotype quality (GQ) > 15. Missingness thresholds were applied according to the target analysis: a relaxed cutoff (maximum missingness up to 40%) was used to generate the GWAS input set, whereas a stricter cutoff (maximum missingness up to 20%) was applied to generate a high-quality subset for population-structure and relatedness analyses. After filtering, 91,048 SNPs were used for GWAS, and 3227 high-quality SNPs were retained for the population analyses described below.

### 4.3. Phylogenetic Inference, STRUCTURE Clustering, PCA, and Pairwise Fst Estimation

Population structure and genetic relationships within the KLC were evaluated using the high-quality SNP set retained for population analyses (*n* = 3227). A maximum-likelihood (ML) phylogenetic tree was constructed in MEGA11 with branch support assessed by 1000 bootstrap replicates, and *L. serriola* was used as an outgroup for rooting. Bayesian clustering was inferred with STRUCTURE, while replicate runs across K values were automated and parallelized using Structure_threader [43]. The optimal K was then determined using the Delta K method of Evanno et al. [44]. Principal component analysis (PCA) and pairwise Fst estimation were conducted using R packages based on the same SNP matrix, and results were summarized by horticultural type for visualization and comparison.

### 4.4. Genome-Wide Association Analyses and SNP Validation by KASP

Genome-wide association studies (GWAS) for CTTB and CTTA were performed using the R package (v.4.4.1) GAPIT (v.3.4) with the filtered SNP set described in Section 4.2 (91,048 SNPs). GWAS was conducted separately for each year using year-specific accession means as trait inputs. To detect loci under complementary model assumptions, four GWAS methods implemented in GAPIT were applied, including MLM, cMLM, FarmCPU, and BLINK.

Candidate SNPs were prioritized through a two-step validation scheme. First, within each year, GWAS hits were checked by comparing allele-group means for CTTB and CTTA, and only SNPs showing clear separation of the mean values with 95% confidence intervals were retained to build year-specific candidate sets. Second, SNPs consistently detected in both years were further validated using a linear mixed model, with SNP genotype class as a fixed effect and accession, year, and block-within-year as random effects. CTTB and CTTA BLUPs (Best Linear Unbiased Predictions) were obtained from the fitted model, and SNPs consistently detected in both years were confirmed by comparing allele-group mean BLUPs and their 95% confidence intervals to support stable allelic effects across years.

For technical validation, a representative candidate SNP (ch7:160010057) was re-genotyped using Kompetitive Allele Specific PCR (KASP) following the workflow described by Kalendar et al. (2022) [45].

### 4.5. Genomic Prediction of CTTB and CTTA Using Validated SNP Panels

The SNP loci used for genomic prediction were selected from a targeted genotyping set generated by genotyping-by-target-sequencing (GBTS). For each locus, locus-specific flanking primers were designed, and Illumina P5 and P7 adapter sequences were appended together with sample-identifying barcodes and index sequences to enable multiplexed library construction. Multiplex PCR was performed to amplify targeted loci across samples, pooled amplicons were used to construct sequencing libraries, and sequencing was conducted on an Illumina platform. SNP calling from GBTS reads followed the same processing strategy as the GBS dataset described above (read preprocessing, reference-based mapping, and variant calling), yielding genotype matrices for downstream prediction analyses.

Genotype-level response values for genomic prediction were obtained as BLUPs from a linear mixed model fitted to the pooled two-year phenotypic data, with genotype, year, and block-within-year treated as random effects. These BLUPs were deregressed using their reliabilities (r_i^2^) to generate DRPs (DRP_i = BLUP_i/r_i^2^), which were modeled with reliability-derived weights (w_i = r_i^2^/(1 − r_i^2^)) to account for heterogeneous precision.

For model input, GBTS-derived genotypes were recoded as −1, 0, or 1 according to the direction of allelic effects. Homozygotes carrying the allele associated with delayed bolting (for CTTB) or delayed anthesis (for CTTA) were coded as 1, homozygotes carrying the alternative allele were coded as −1, and heterozygotes were coded as 0. The input genotype used for genomic prediction for CTTB, CTTA, and the pooled panel is provided in Supplementary Tables S7–S9.

Genotype-level response values for genomic prediction were obtained as BLUPs from a linear mixed model fitted to the pooled two-year phenotypic data, with genotype, year, and block-within-year treated as random effects. These BLUPs were deregressed using their reliabilities (r_i^2^) to generate deregressed BLUPs (DRP_i = BLUP_i/r_i^2^), which were modeled with reliability-derived weights (w_i = r_i^2^/(1 − r_i^2^)) to account for heterogeneous precision [x,y] (Tables S7–S9).

Genomic prediction for CTTB and CTTA was implemented in R (v.4.5.2) using validated candidate SNP panels. Trait-specific panels were constructed from the loci validated for each trait, and a pooled panel was additionally generated by combining the validated CTTB- and CTTA-associated loci into a single marker set. Prediction models included a fixed-effect model (FEM), RR-BLUP, LASSO, elastic net (α = 0.5), random forest (RF), and RKHS. Model performance was evaluated using five-fold cross-validation with 25 runs (5 folds × 5 repeats). To prevent information leakage among genotypes sharing identical marker profiles, we used a balanced Group K-Fold scheme in which accessions with the same marker profile were kept in the same fold, and folds were constructed via a greedy, size-balancing assignment based on profile group sizes.

Prediction accuracy was summarized using R^2^, Pearson’s r, and RMSE, and calibration was assessed using the slope (b) and intercept (a) from the regression of observed on predicted values.

## 5. Conclusions

Warm-season bolting remains a major constraint on lettuce production, and this study provides a practical route for breeding delayed bolting under warm greenhouse and summer-like conditions. Using the KNOU lettuce core collection (KLC), we showed that a horticultural type-structured but genetically connected panel can support effective association mapping when population structure and kinship are properly controlled. Across two years, CTTB and CTTA showed broad quantitative variation and high heritability, and multi-model GWAS consistently identified a reproducible chromosome 7 hotspot, together with additional loci showing trait- and year-dependent effects. Importantly, validated loci were translated into compact GBTS-based SNP panels that enabled repeatable genomic prediction with a small marker set, supporting early genotype-based elimination of low-ranking candidates without additional phenotyping. Taken together, these results provide a practical, updatable SNP panel framework for delayed-bolting breeding in lettuce.

## Figures and Tables

**Table 1:** A summary of phenotypic variation and quantitative genetic parameters in cumulative temperature to bolting (CTTB) and cumulative temperature to anthesis (CTTA) across major lettuce types within the KNOU lettuce core (KLC).

Types	CTTB ^z^					CTTA ^y^				
	Mean	SE ^x^	GCV ^w^	PCV ^v^	H ^u^	Mean	SE	GCV	PCV	H
Total	1118.83	2.50	11.53	12.96	0.79	1603.62	3.18	7.02	8.13	0.74
BH ^t^	1085.00 c	2.64	4.57	5.97	0.58	1613.49 b	6.09	4.27	5.76	0.55
CH ^s^	1225.52 a	5.13	7.33	8.89	0.68	1682.54 a	7.70	3.02	4.76	0.40
RM ^r^	1065.55 d	4.32	9.52	10.74	0.79	1564.52 b	6.78	7.98	9.10	0.77
LF ^q^	1131.37 b	4.84	14.00	15.71	0.79	1600.14 c	5.24	8.24	9.23	0.80

^q,r,s,t^, LF, RM, CH, and BH denote leaf, romaine, crisphead, and butterhead lettuce types, respectively; ^x^, SE indicates standard error of mean; ^v,w^, PCV and GCV stand for the phenotypic and genotypic coefficient of variation, respectively; ^u^, H represents broad-sense heritability; ^y,z^, CTTB and CTTA refer to cumulative temperature to bolting and cumulative temperature to anthesis, respectively. For CTTB and CTTA mean values, different lowercase letters indicate significant differences among lettuce types according to Tukey’s HSD test (*p* < 0.05).

**Table 2:** A summary of significant SNPs associated with cumulative temperature to bolting (CTTB) was identified over two years using multiple GWAS models.

Phenotype	Chr ^z^	Position	Ref. ^y^	Alt. ^x^	Significance ^w^	MAF ^v^	Nobs ^u^	Model ^t^
CTTB_1 ^s^	1	59238807	G	A	10.19	0.16	283	M, C, F, B
	1	59765951	C	T	4.77	0.18	283	M, C
	2	196799584	A	G	4.75	0.19	283	M, C, F
	5	223529019	A	G	5.49	0.26	283	M, C, B
	6	162425335	T	C	5.61	0.36	283	F
	7	160010057	T	G	6.61	0.36	283	M, C
	7	162215421	G	C	6.04	0.36	283	M, C
	7	162466345	A	C	5.54	0.39	283	M, C, F
CTTB_2 ^r^	1	59238807	G	A	10.03	0.16	278	M, C, F, B
	4	10791955	T	A	9.31	0.48	278	M, C, F, B
	4	71593965	A	T	4.5	0.15	278	M, C
	5	59213356	C	G	7.35	0.47	278	M, C, B
	5	80193683	G	A	6.99	0.44	278	F
	7	98088697	C	T	5.01	0.21	278	M, C
	7	159939239	T	C	5.51	0.49	278	M, C
	7	160010057	T	G	8.25	0.37	278	M, C, F, B
	7	162215421	G	C	6.84	0.37	278	M, C
	7	162466345	A	C	6.68	0.40	278	M, C

^r,s^, CTTB_1 and CTTB_2 represent the cumulative temperature to bolting phenotypes measured in Year 1 and Year 2, respectively; ^t^, GWAS models detecting the SNP are abbreviated as M (MLM), C (cMLM), F (FamCPU), and B (BLINK); ^u,v^, Nobs and MAF refer to the number of observations and minor allele frequency, respectively; ^w^, The significance level is expressed as −log10(P) values; ^x,y,z^, Ref., and Chr denote the alternative allele, reference allele, and relevant chromosomes, respectively.

**Table 3:** Cross-validation performance and calibration diagnostics for pooled-panel prediction of CTTB and CTTA from previously validated SNPs. The pooled panel combines the validated CTTB- and CTTA-associated SNPs into a single set. Results from five-fold CV (5 folds × 5 repeats = 25 runs) are reported as mean ± standard deviation for R^2^, Pearson’s r, RMSE, and the calibration slope *b* from $y=a+b\hat{y}$. Algorithms compared include FEM, RR-BLUP, LASSO, elastic net (α = 0.5), random forest (RF), and RKHS.

Trait	Model	n	R^2^	Pearson r	RMSE	Slope
CTTB ^z^	FEM	25	0.36 ± 0.08	0.60 ± 0.07	120.03 ± 10.99	1.02 ± 0.25
	RR-BLUP	25	0.37 ± 0.08	0.60 ± 0.07	119.23 ± 11.94	1.14 ± 0.26
	LASSO	25	0.36 ± 0.08	0.60 ± 0.07	120.19 ± 11.88	1.11 ± 0.28
	EN (a = 0.5)	25	0.36 ± 0.08	0.60 ± 0.07	119.92 ± 11.90	1.13 ± 0.27
	RF	25	0.41 ± 0.10	0.63 ± 0.08	114.50 ± 13.37	1.16 ± 0.22
	RKHS	25	0.39 ± 0.09	0.62 ± 0.08	118.48 ± 12.97	1.25 ± 0.27
CTTA ^y^	FEM	25	0.27 ± 0.11	0.51 ± 0.12	117.38 ± 13.66	0.91 ± 0.25
	RR-BLUP	25	0.29 ± 0.11	0.52 ± 0.12	116.09 ± 14.02	1.06 ± 0.29
	LASSO	25	0.28 ± 0.11	0.52 ± 0.12	116.93 ± 14.19	1.04 ± 0.31
	EN (a = 0.5)	25	0.28 ± 0.11	0.52 ± 0.12	116.71 ± 14.13	1.06 ± 0.31
	RF	25	0.35 ± 0.12	0.59 ± 0.10	110.00 ± 14.06	1.08 ± 0.22
	RKHS	25	0.32 ± 0.13	0.55 ± 0.12	116.50 ± 16.54	1.21 ± 0.31

^z,y^, CTTB, and CTTA denote the cumulative temperature to bolting and to anthesis, respectively; both were measured in Years 1 and 2.

## Data Availability

The original contributions presented in this study are included in the article/Supplementary Material. Further inquiries can be directed to the corresponding author.

## Supplementary Materials

The following supporting information can be downloaded at: https://www.mdpi.com/article/10.3390/plants15111621/s1, Figure S1: Non-compressed maximum-likelihood (ML) phylogenetic tree of the KNOU lettuce core collection (KLC). Branch lengths represent the estimated number of nucleotide substitutions per site (genetic distance). The tree was inferred from 3227 high-quality SNPs and rooted with *Lactuca serriola* as an outgroup. Node support values are bootstrap percentages from 1000 replicates. Figure S2: Determination of the optimal number of STRUCTURE clusters (K) using the Evanno method. Delta K is shown across K = 1–8, supporting K = 4 as the optimal clustering solution. Figure S3: Type-wise comparisons of bolting and flowering thermal-time traits in the KLC. (a) CTTB and (b) CTTA are shown by horticultural type. Points represent accession values, and diamonds indicate group means with confidence intervals as displayed. Letters above groups indicate significant differences based on all-pairs Tukey–Kramer tests (*p* < 0.05). Figure S4: Relationship between CTTB and CTTA across accessions. The scatter plot shows accession-level values with a fitted linear regression line. Model summary (R-squared and standard error) and ANOVA results are reported on the panel. Figure S5: Manhattan plots of GWAS results for cumulative temperature to anthesis (CTTA) across two years and four GWAS methods. Four GWAS methods—Blink, FamCPU, cMLM, and MLM—were applied to identify loci associated with CTTA across two years. The *x*-axis represents genomic positions across nine chromosomes, while the *y*-axis shows the −log10(*p*-value or P) of SNP associations. The green horizontal solid line indicates the method-specific significance threshold. Gray dashed lines highlight regions with candidate SNPs that either repeatedly exceeded the threshold or showed distinct association patterns. Figure S6: Year 1 allele-group mean differences for CTTB at candidate loci. Interval plots show Year 1 CTTB values stratified by allele group for selected SNPs (chromosome and position shown on each panel). Red points indicate allele-group means, red horizontal lines indicate confidence intervals, and black dots represent accession-level values. Figure S7: Year 2 allele-group mean differences for CTTB at candidate loci. Plots are shown as in Figure S6 using Year 2 CTTB data. Figure S8: Technical validation of a lead Chr7 marker for CTTB using KASP. (a) CTTB BLUPs stratified by GBS genotype calls at ch7:160010057, including the GBS missing/ambiguous category (“N”). (b) CTTB BLUPs stratified by KASP genotype calls after re-genotyping. Red points indicate allele-group mean BLUPs with 95% confidence intervals, and black dots represent accession-level BLUPs. Figure S9: Year 1 allele-group mean differences for CTTA at candidate loci. Interval plots show Year 1 CTTA values stratified by allele group for selected SNPs (chromosome and position shown on each panel). Red points indicate allele-group means, red horizontal lines indicate confidence intervals, and black dots represent accession-level values. Figure S10: Year 2 allele-group mean differences for CTTA at candidate loci. Plots are shown as in Figure S9 using Year 2 CTTA data. Figure S11: Technical validation of a lead Chr7 marker for CTTA using KASP. (a) CTTA BLUPs stratified by GBS genotype calls at ch7:160010057, including the GBS missing/ambiguous category (“N”). (b) CTTA BLUPs stratified by KASP genotype calls after re-genotyping. Red points indicate allele-group mean BLUPs with 95% confidence intervals, and black dots represent accession-level BLUPs. Figure S12: BLUP-based allele-effect validation for key CTTA loci. Interval plots show CTTA BLUPs stratified by allele group at highlighted loci (chromosome and position shown on each panel). Red points indicate allele-group mean BLUPs with 95% confidence intervals, and black dots represent accession-level BLUPs. Figure S13: Lettuce type-wise distributions of CTTA BLUPs by allele group at a major locus. Density curves show CTTA BLUP distributions stratified by allele group in (a) the full panel and within (b) BH, (c) LF, (d) RM, and (e) CH. Vertical dashed lines mark group means as displayed, illustrating conserved direction of allelic effects with type-dependent effect magnitudes and limited within-type contrast where the locus is near-fixed. Figure S14: Concordance between GBTS and KASP genotype calls. Agreement is summarized for 288 paired genotype calls using a mosaic plot and a contingency table. Call consistency metrics, including per-allele concordance and heterozygote inconsistency, are reported in the figure. Figure S15: Full-data fits of genomic prediction models for CTTB using validated SNP panels. Panels show observed CTTB BLUPs versus model-based predictions for FEM, RR-BLUP, LASSO, EN (alpha = 0.5), RF, and RKHS. Regression lines and prediction interval guides are displayed on each panel as shown. Figure S16: Full-data fits of genomic prediction models for CTTA using validated SNP panels. Panels are shown as in Figure S15 for CTTA BLUPs across the same set of prediction models.; Table S1: GWAS-identified SNPs associated with CTTA in each year. For Year 1 (CTTA_1) and Year 2 (CTTA_2), the table lists chromosome (Chr) and physical position (Pos), reference (Ref) and alternative (Alt) alleles, association significance reported as −log_10_(*p)*, minor allele frequency (MAF), and the number of accessions used (Nobs). The “Model” column indicates which GWAS methods detected the SNP (M, MLM; C, cMLM; F, FarmCPU; B, BLINK).; Table S2: Model calibration summary for SNP-based prediction of CTTB using the validated CTTB SNP panel. For each algorithm, calibration is reported from the linear relationship between observed values and model predictions ($y=a+b\hat{y}$), together with S (RMSE) and R^2^ (%). As an interpretable proxy for prediction uncertainty, the approximate 95% prediction interval (PI) half-width near the predictor mean is about 1.96 × S (full width about 3.92 × S). Abbreviations: FEM, fixed-effect model; RR-BLUP, ridge-regression BLUP; EN(0.5), elastic net with alpha = 0.5; RKHS, reproducing-kernel Hilbert space; RF, random forest. Units match the response scale (CTTB).; Table S3: Five-fold cross-validation (cv) performance and calibration diagnostics for SNP-based prediction of CTTB using the validated CTTB SNP panel. Results are summarized as mean and standard deviation across 25 cross-validation runs (5 folds × 5 repeats). Metrics include mean_r^2^ (R^2^), mean_r (Pearson r), mean_rmse (RMSE), and mean_slope (calibration slope *b* from regressing observed on predicted values). The column mean_slope0 reports the slope from the same regression with the intercept constrained to 0, and corresponding standard deviations are provided for each metric. “*n*” indicates the number of SNP loci used in the panel for the corresponding analysis.; Table S4: Model calibration summary for SNP-based prediction of CTTA using the validated CTTA SNP panel. Calibration is reported from $y=a+b\hat{y}$, together with S (RMSE), R^2^ (%), and the approximate 95% PI half-width near the predictor mean (about 1.96 × S). Abbreviations are as in Table S2. Units match the response scale (CTTA).; Table S5: Five-fold cross-validation (cv) performance and calibration diagnostics for SNP-based prediction of CTTA using the validated CTTA SNP panel. Results are summarized as mean and standard deviation across the CV. Metrics include mean_r^2^ (R^2^), mean_r (Pearson r), mean_rmse (RMSE), mean_slope (calibration slope), and mean_slope0 (slope with intercept constrained to 0), with corresponding standard deviations reported for each metric. “*n*” indicates the number of SNP loci used in the panel for the corresponding analysis.; Table S6: Passport information for accessions in the KNOU lettuce core collection (KLC). The table summarizes accession identifiers and associated passport data, including source/origin information, horticultural type classification, and additional descriptors such as leaf color.; Table S7: Input datasets used for genomic prediction of CTTB. The table provides the marker and phenotype inputs used for SNP-panel prediction analyses.; Table S8: Input datasets used for genomic prediction of CTTA. The table provides the marker and phenotype inputs used for SNP-panel prediction analyses.; Table S9: Input datasets used for genomic prediction of the pooled CTTB and CTTA dataset. This table summarizes the marker and phenotype inputs used for SNP-panel-based prediction analyses.
