# Supplementary material for: GWAS-Guided Compact SNP Panels Enable Breeding-Relevant Prediction of Bolting and Flowering Timing of Lettuce
Source: Plants (Basel). 2026 May 25;15(11):1621. doi: 10.3390/plants15111621 (PMC13259484; doi:10.3390/plants15111621)
Supplement: Supplementary file 1 [file plants-15-01621-s001.zip › plants-4255172-supplementary.pdf]

Figure S1.

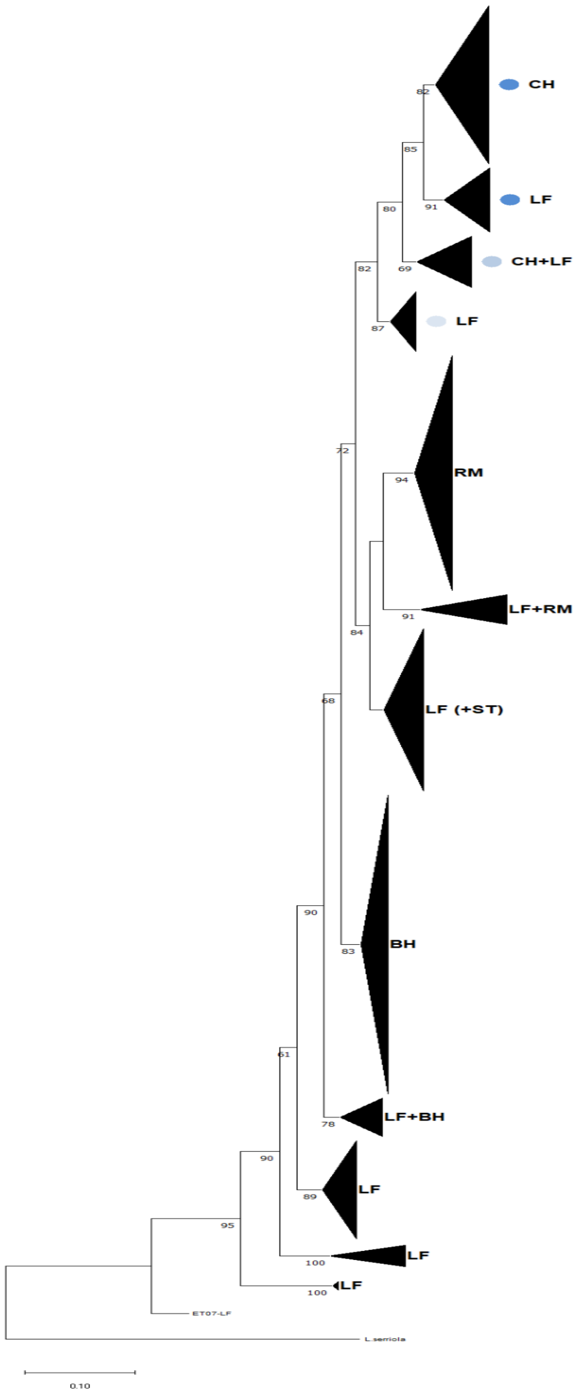
CH
LF
CH+LF
LF
RM
LF+RM
LF (+ST)
BH
LF+BH
LF
LF
LF
ET07-LF
L.serriola
0.10

Figure S2.

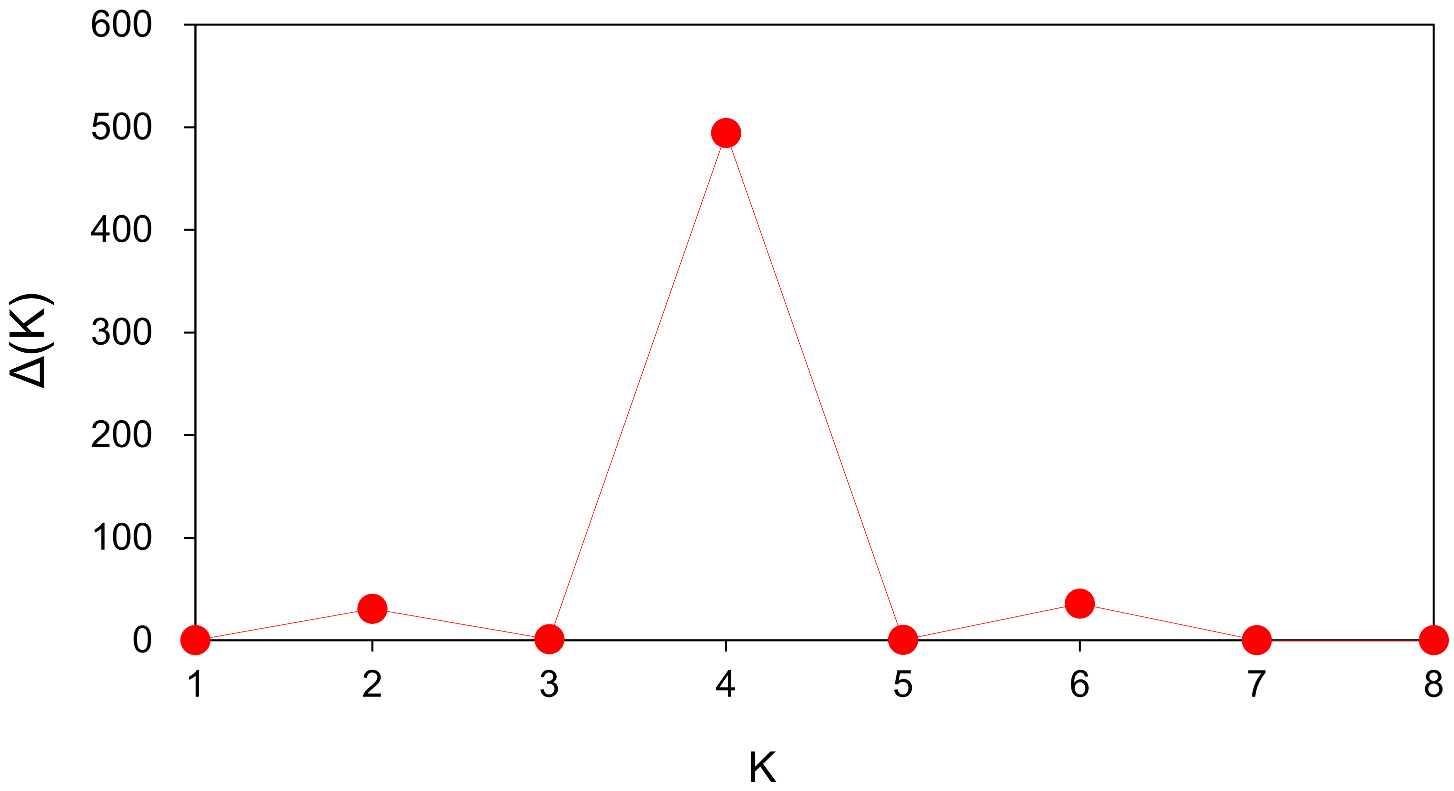

Figure S3.

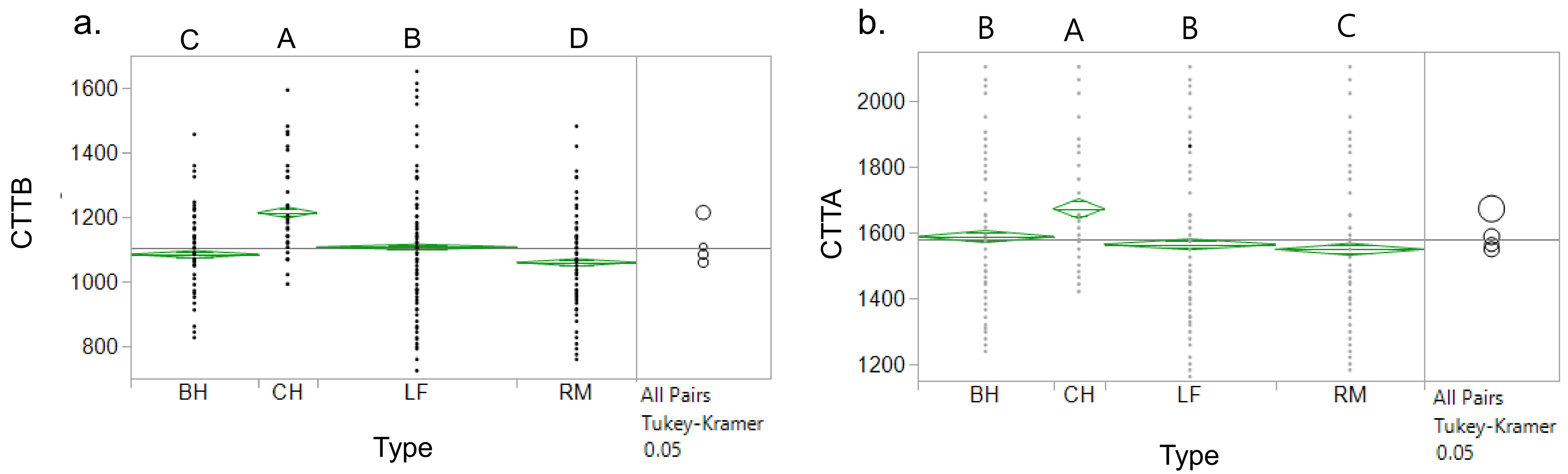
a.
C
A
B
D
1600
1400
1200
1000
800
CTTB
BH
CH
LF
RM
All Pairs
Tukey-Kramer
0.05
Type
b.
B
A
B
C
2000
1800
1600
1400
1200
CTTA
BH
CH
LF
RM
All Pairs
Tukey-Kramer
0.05
Type

Figure S4.

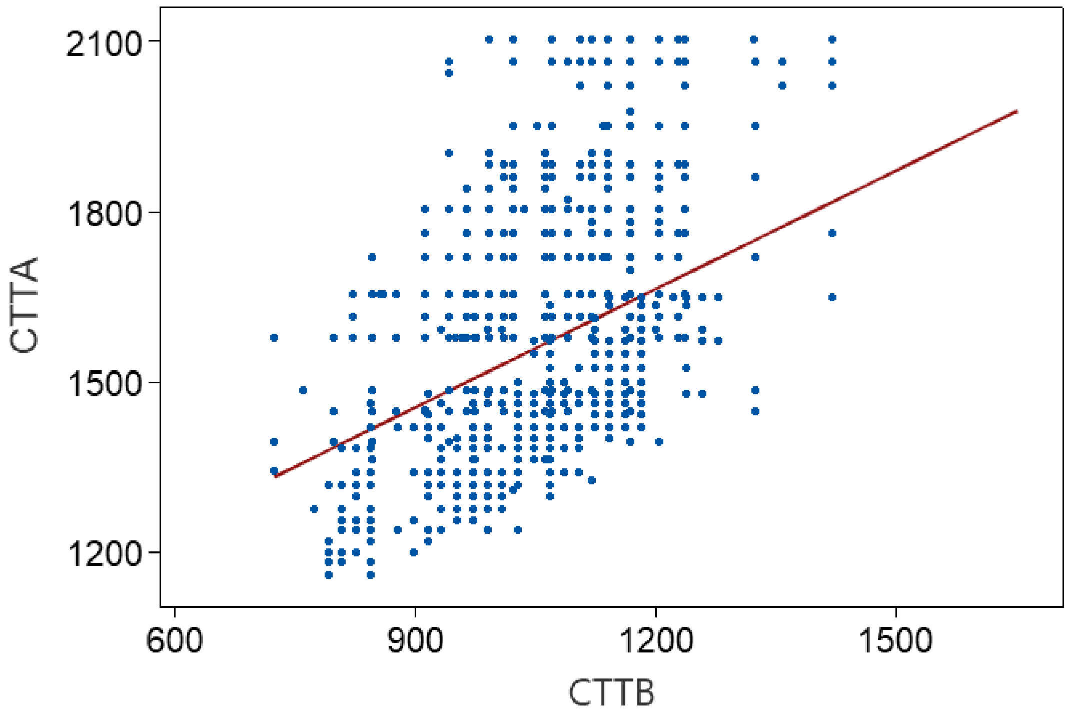

Model Summary

S	R-sq	R-sq(adj)
176.555	18.45%	18.39%

Analysis of Variance

Source	DF	SS	MS	F	P
Regression	1	10076162	10076162	323.25	0.000
Error	1429	44544104	31172		
Total	1430	54620266			

Figure S5.

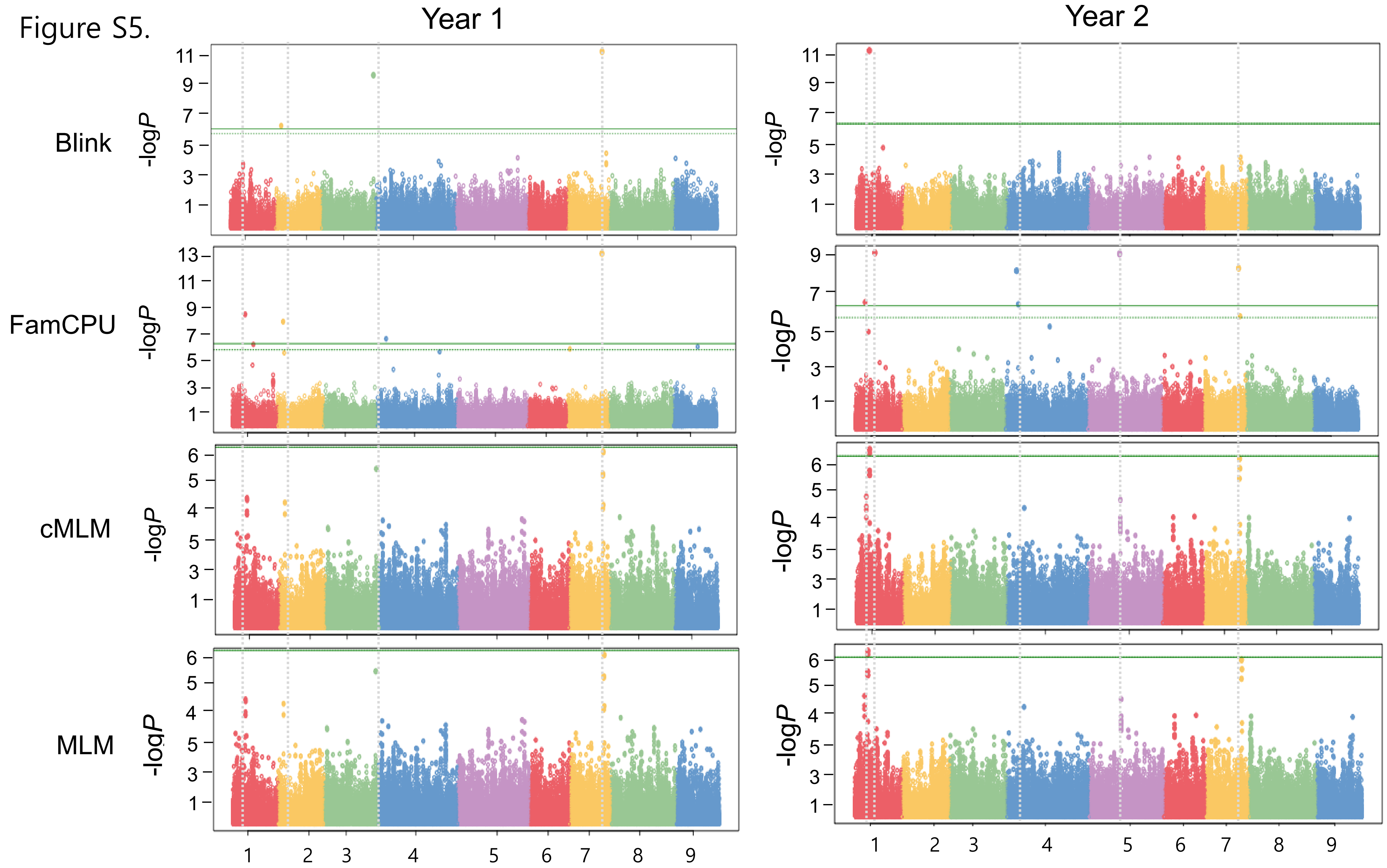

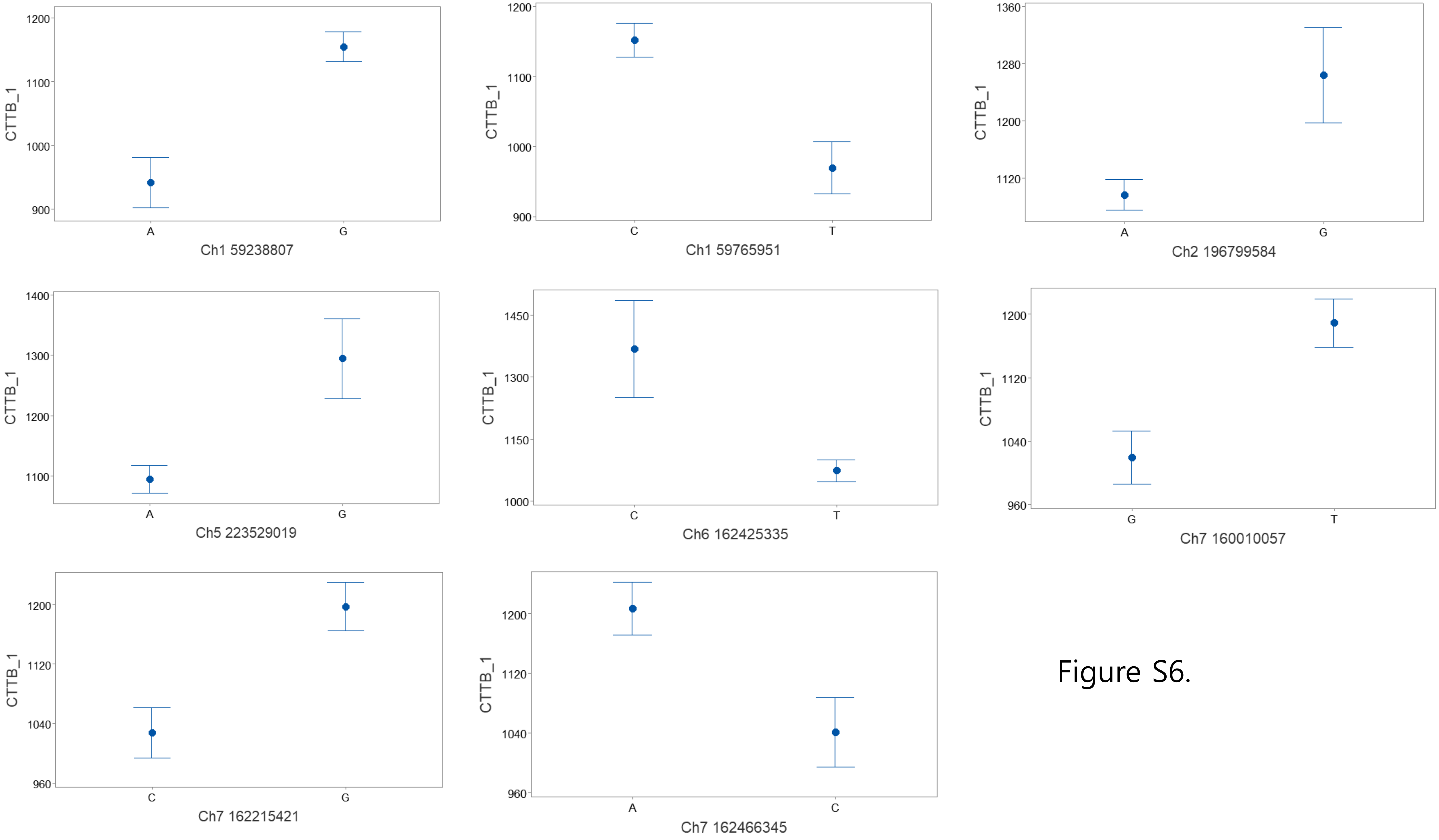
CTTB_1
Ch1 59238807
Ch1 59765951
Ch2 196799584
Ch5 223529019
Ch6 162425335
Ch7 160010057
Ch7 162215421
Ch7 162466345

Figure S6.

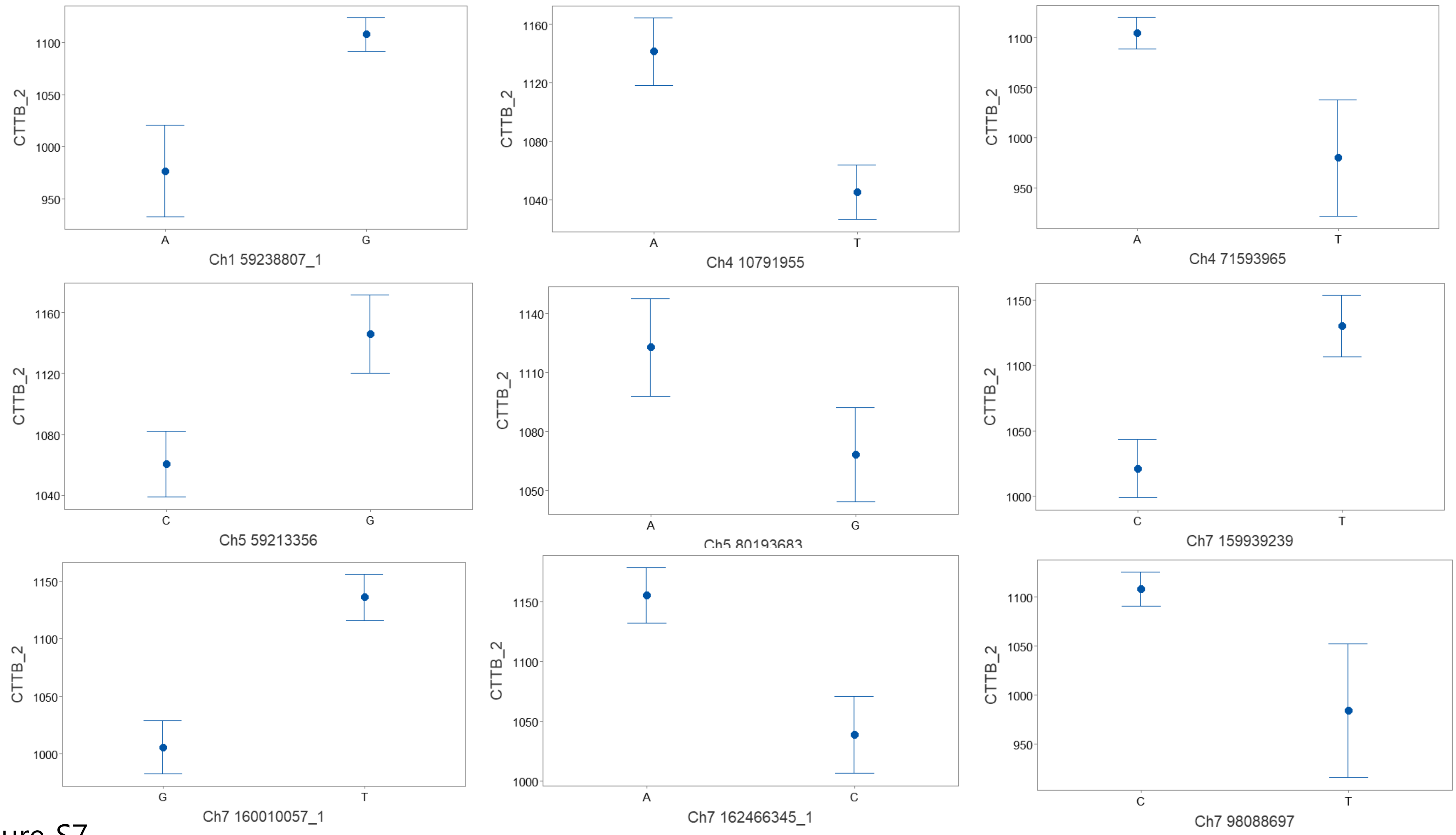
CTTB_2
Ch1 59238807_1
Ch4 10791955
Ch4 71593965
Ch5 59213356
Ch5 80193683
Ch7 159939239
Ch7 160010057_1
Ch7 162466345_1
Ch7 98088697

Figure S7.

Figure S8.

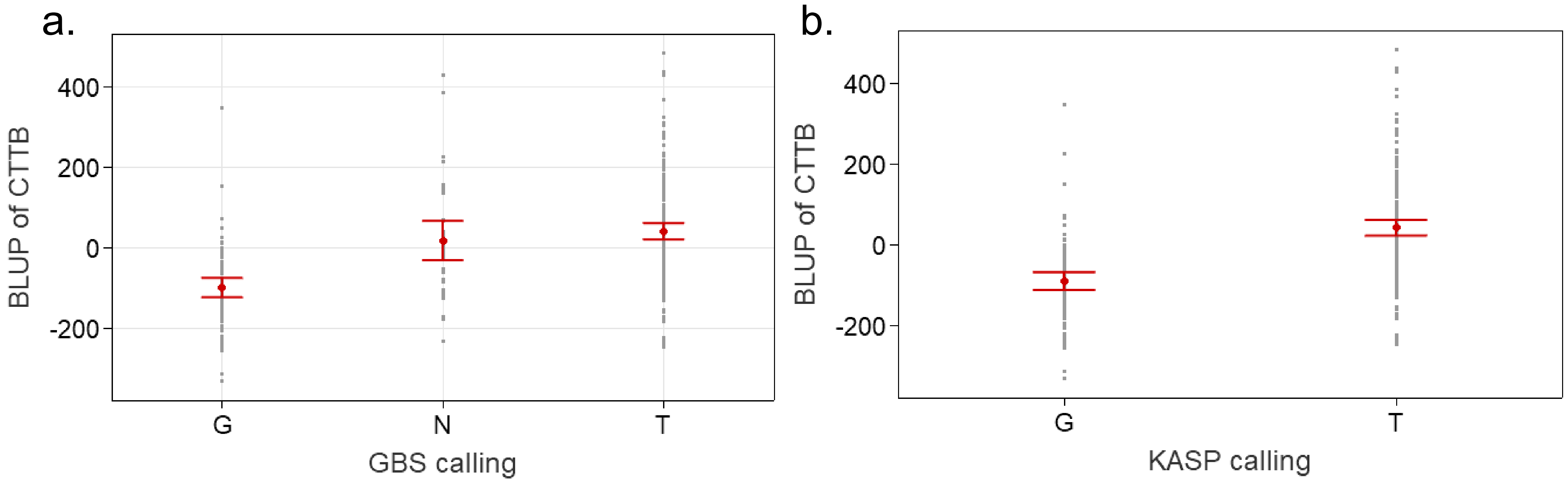
a.
BLUP of CTTB
400
200
0
-200
G
N
T
GBS calling
b.
BLUP of CTTB
400
200
0
-200
G
T
KASP calling

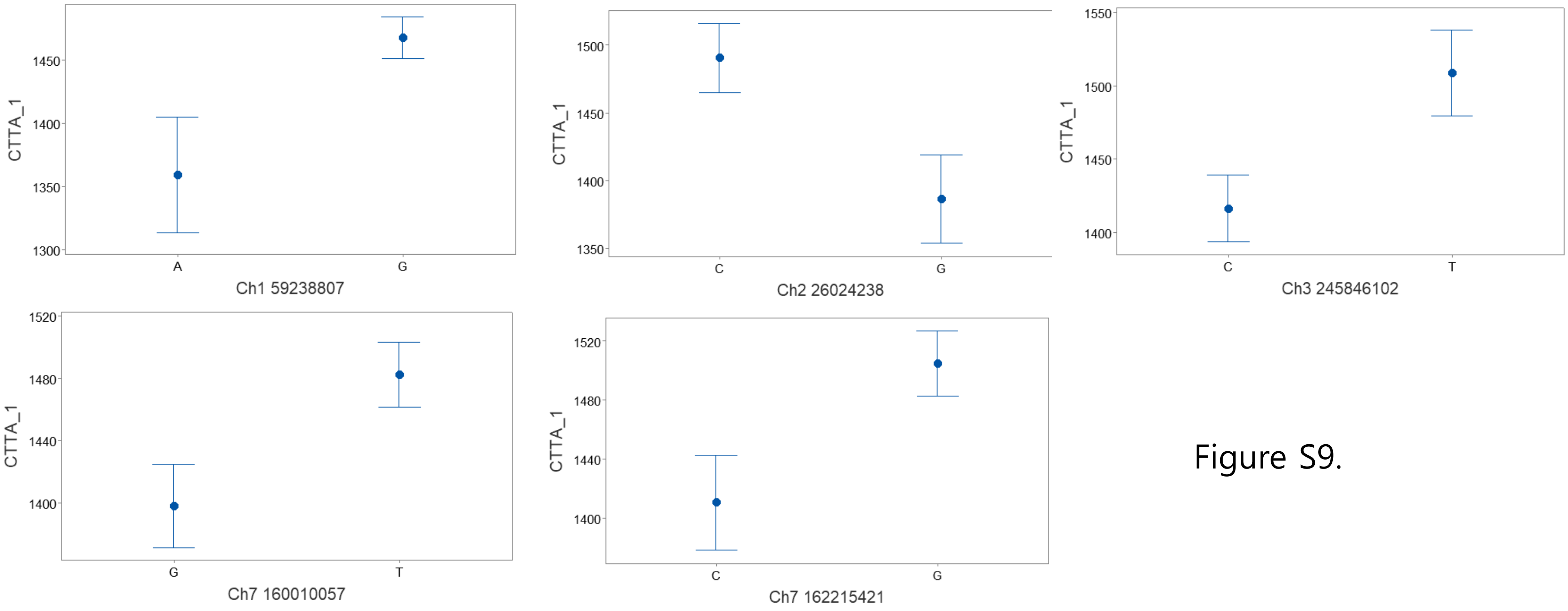
CTTA_1
1300
1350
1400
1450
A
G
Ch1 59238807
1350
1400
1450
1500
C
G
Ch2 26024238
1400
1450
1500
1550
C
T
Ch3 245846102
1400
1440
1480
1520
G
T
Ch7 160010057
1400
1440
1480
1520
C
G
Ch7 162215421

Figure S9.

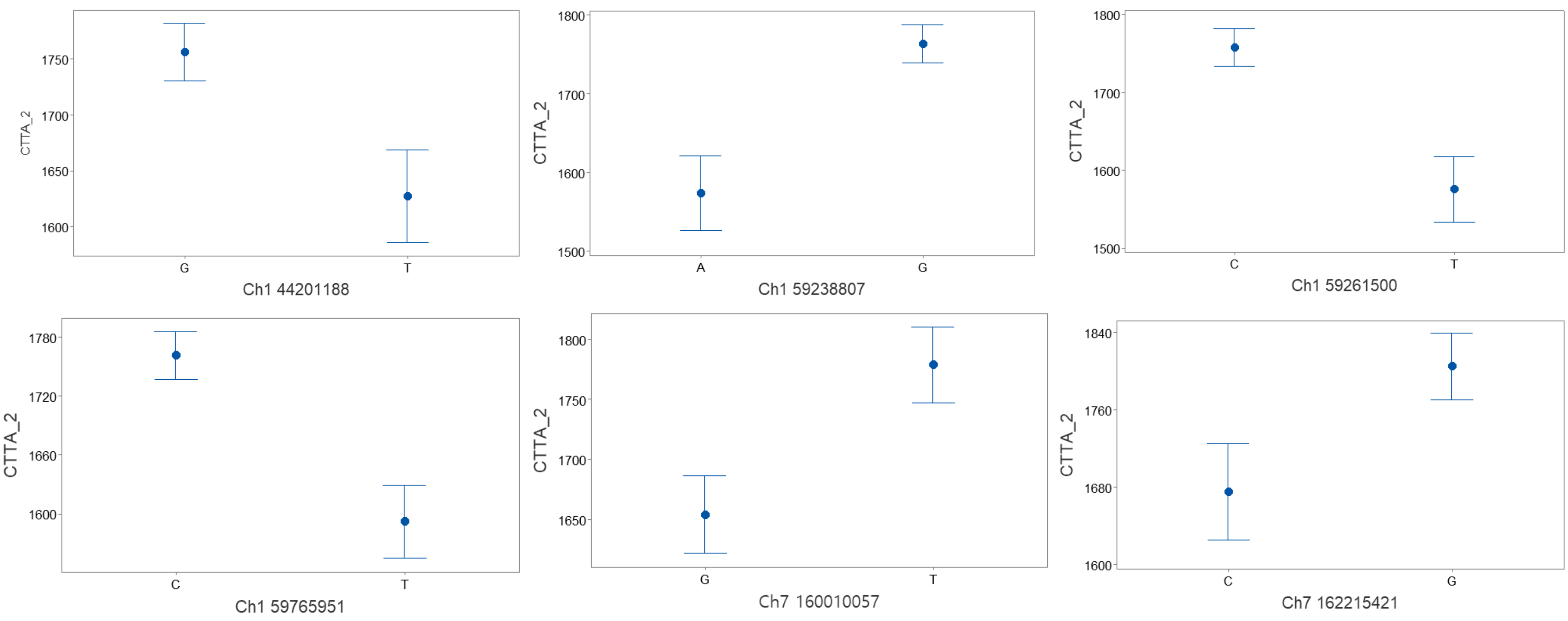
CTTA_2
1750
1700
1650
1600
G
T
Ch1 44201188
CTTA_2
1800
1700
1600
1500
A
G
Ch1 59238807
CTTA_2
1800
1700
1600
1500
C
T
Ch1 59261500
CTTA_2
1780
1720
1660
1600
C
T
Ch1 59765951
CTTA_2
1800
1750
1700
1650
G
T
Ch7 160010057
CTTA_2
1840
1760
1680
1600
C
G
Ch7 162215421

Figure S10.

Figure S11.

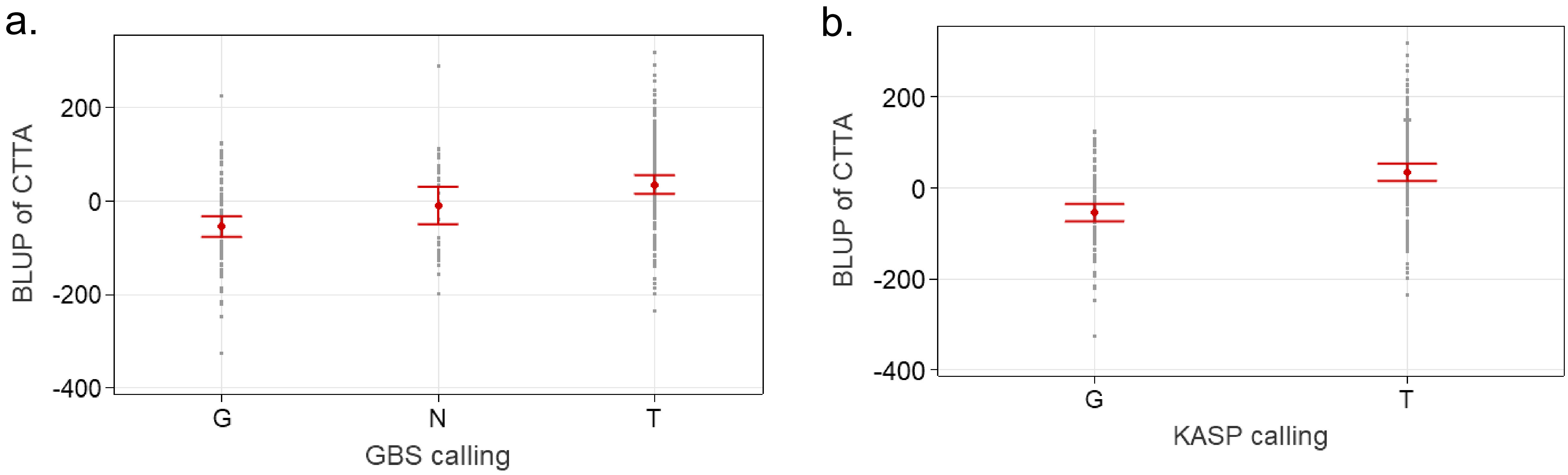
a.
BLUP of CTTA
200
0
-200
-400
G
N
T
GBS calling
b.
BLUP of CTTA
200
0
-200
-400
G
T
KASP calling

Figure S12.

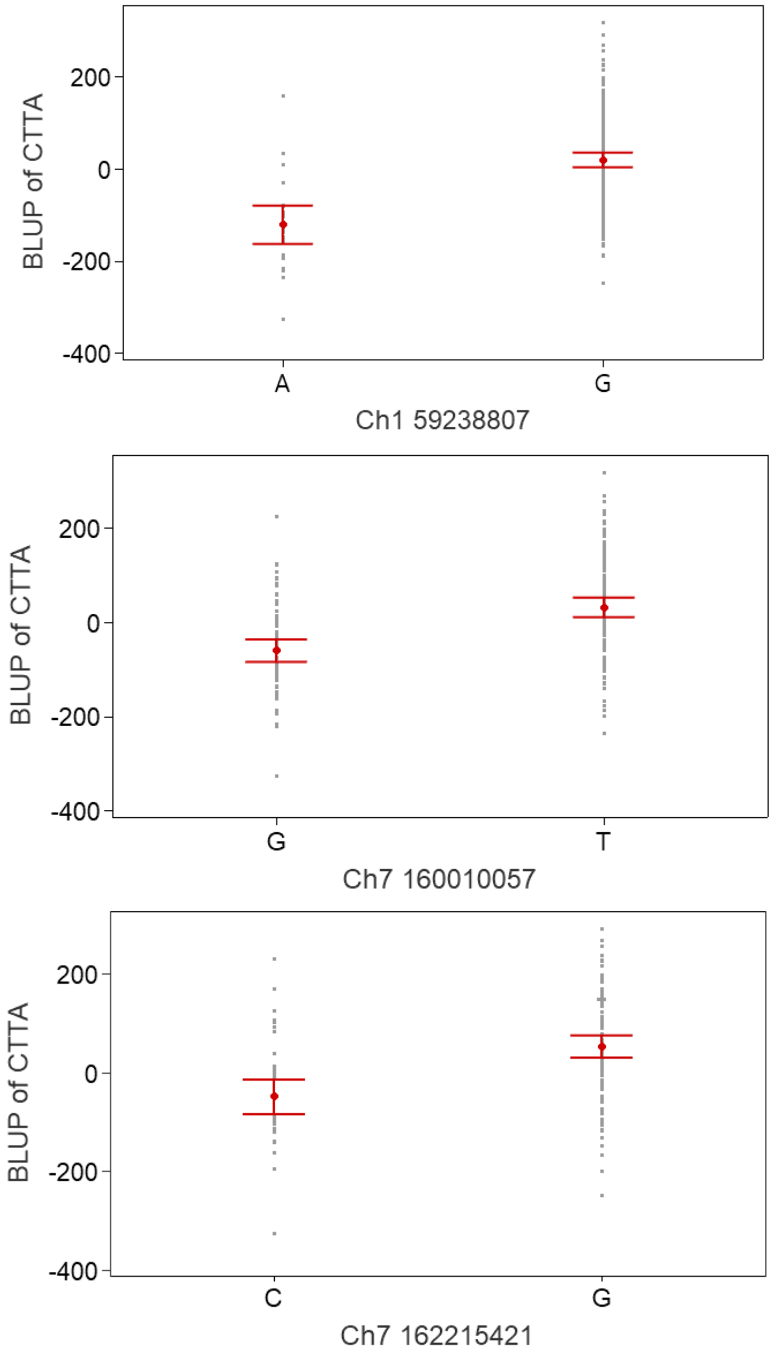
BLUP of CTTA
200
0
-200
-400
A
G
Ch1 59238807
BLUP of CTTA
200
0
-200
-400
G
T
Ch7 160010057
BLUP of CTTA
200
0
-200
-400
C
G
Ch7 162215421

Figure S13.

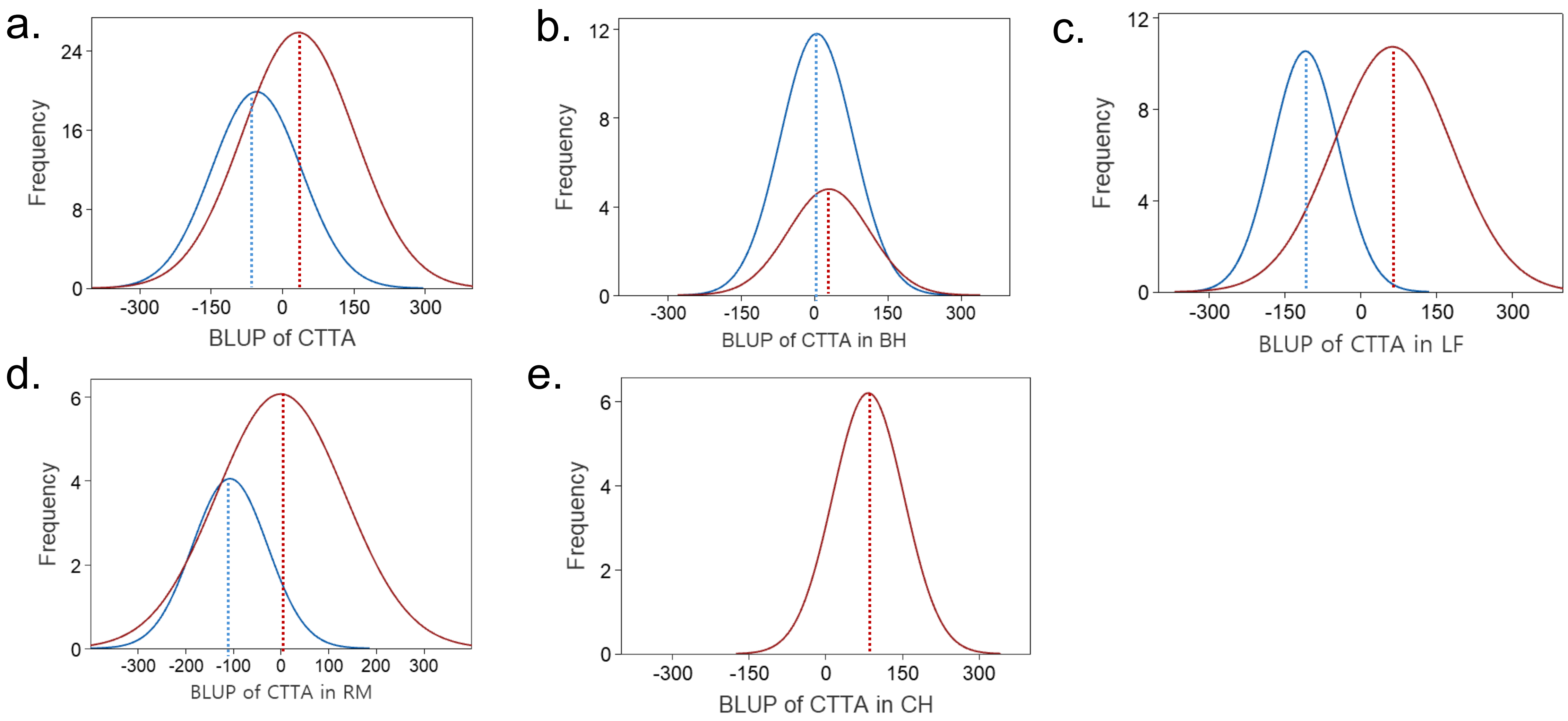
a.
Frequency
24
16
8
0
-300
-150
0
150
300
BLUP of CTTA
b.
Frequency
12
8
4
0
-300
-150
0
150
300
BLUP of CTTA in BH
c.
Frequency
12
8
4
0
-300
-150
0
150
300
BLUP of CTTA in LF
d.
Frequency
6
4
2
0
-300
-200
-100
0
100
200
300
BLUP of CTTA in RM
e.
Frequency
6
4
2
0
-300
-150
0
150
300
BLUP of CTTA in CH

Figure S14.

GBTS vs KASP(288 data points)

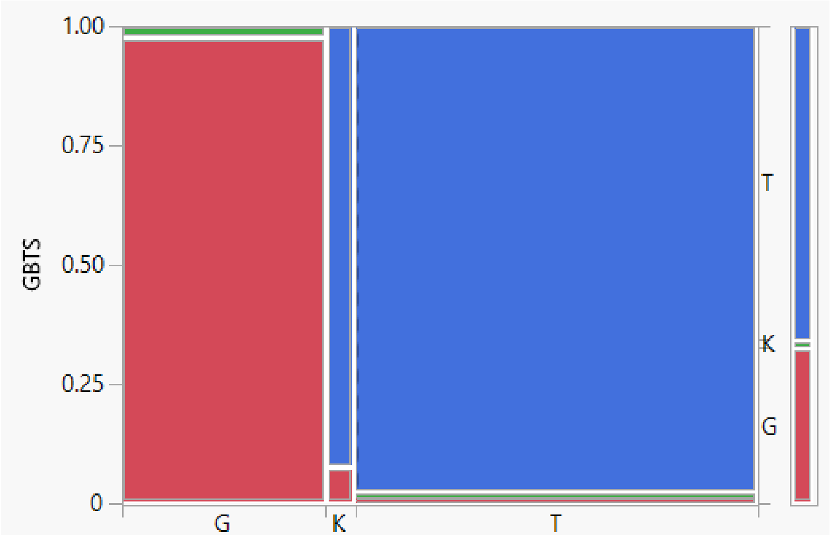

G calling	97.85%
T calling	97.80%
G→T	1.10%
T→G	0.00%
Hetero calling inconsistency	3.25%

		GBTS			
	Count<br>Total %<br>Col %<br>Row %	G	K	T	Total
KASP	G	91	2	0	93
		31.60	0.69	0.00	32.29
		96.81	50.00	0.00	
		97.85	2.15	0.00	
	K	1	0	12	13
		0.35	0.00	4.17	4.51
		1.06	0.00	6.32	
		7.69	0.00	92.31	
	T	2	2	178	182
		0.69	0.69	61.81	63.19
		2.13	50.00	93.68	
		1.10	1.10	97.80	
	Total	94	4	190	288
		32.64	1.39	65.97	

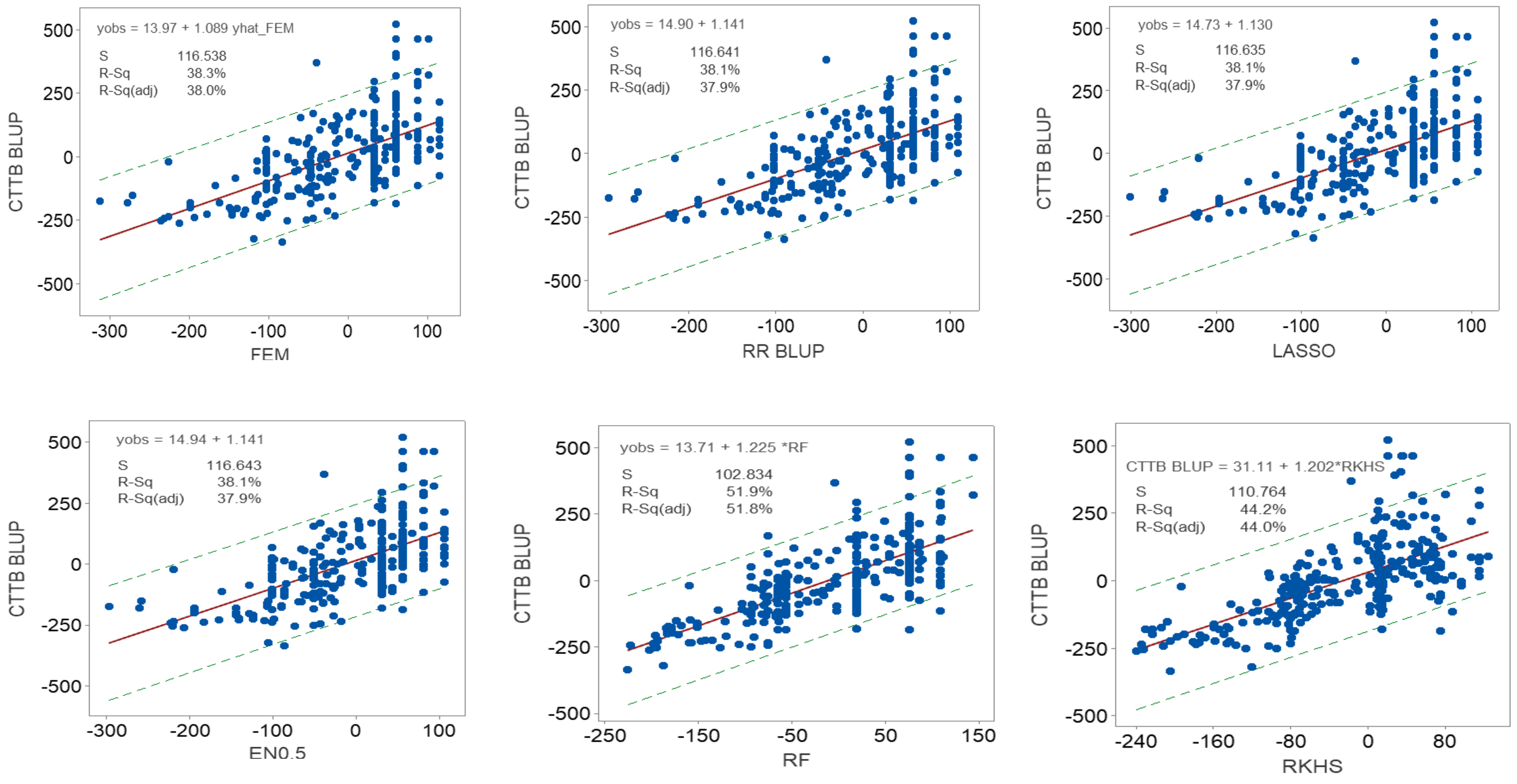

yobs = 13.97 + 1.089 yhat_FEM
S 116.538
R-Sq 38.3%
R-Sq(adj) 38.0%
CTTB BLUP
FEM
yobs = 14.90 + 1.141
S 116.641
R-Sq 38.1%
R-Sq(adj) 37.9%
RR BLUP
yobs = 14.73 + 1.130
S 116.635
R-Sq 38.1%
R-Sq(adj) 37.9%
LASSO
yobs = 14.94 + 1.141
S 116.643
R-Sq 38.1%
R-Sq(adj) 37.9%
EN0.5
yobs = 13.71 + 1.225 *RF
S 102.834
R-Sq 51.9%
R-Sq(adj) 51.8%
RF
CTTB BLUP = 31.11 + 1.202*RKHS
S 110.764
R-Sq 44.2%
R-Sq(adj) 44.0%
RKHS

Figure S15.

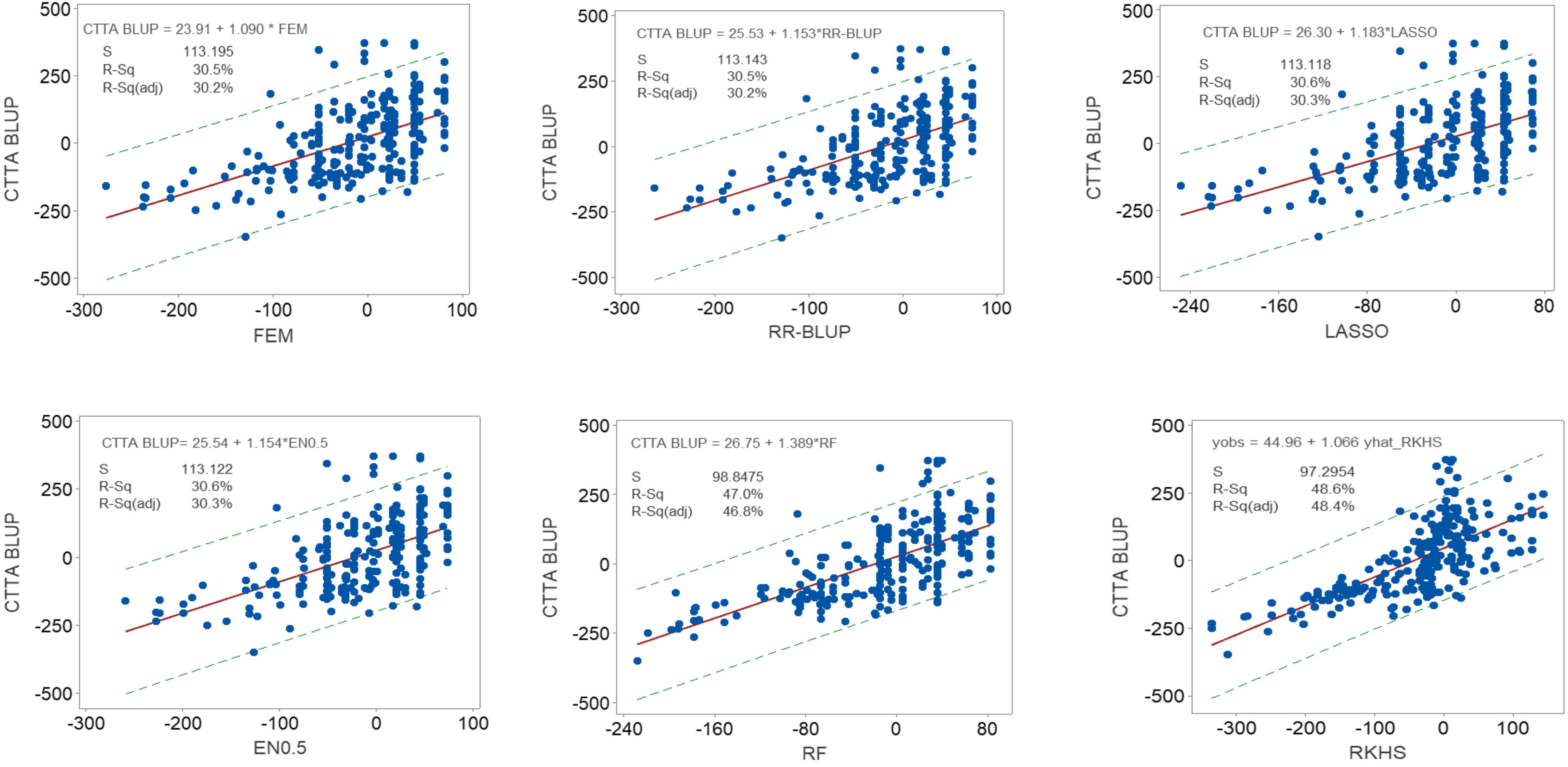

CTTA BLUP = 23.91 + 1.090 * FEM
S 113.195
R-Sq 30.5%
R-Sq(adj) 30.2%
CTTA BLUP
FEM
CTTA BLUP = 25.53 + 1.153*RR-BLUP
S 113.143
R-Sq 30.5%
R-Sq(adj) 30.2%
CTTA BLUP
RR-BLUP
CTTA BLUP = 26.30 + 1.183*LASSO
S 113.118
R-Sq 30.6%
R-Sq(adj) 30.3%
CTTA BLUP
LASSO
CTTA BLUP= 25.54 + 1.154*EN0.5
S 113.122
R-Sq 30.6%
R-Sq(adj) 30.3%
CTTA BLUP
EN0.5
CTTA BLUP = 26.75 + 1.389*RF
S 98.8475
R-Sq 47.0%
R-Sq(adj) 46.8%
CTTA BLUP
RF
yobs = 44.96 + 1.066 yhat_RKHS
S 97.2954
R-Sq 48.6%
R-Sq(adj) 48.4%
CTTA BLUP
RKHS

Figure S16.

Table S1.

Phenotype	Chr.	Pos.	Ref. Allele	Alt. Allele	Significance (-log*P*)	MAF	Nobs	Model
CTTA_1	1	59238807	G	A	8.49	0.17	230	M, C, F, B
	2	26024238	C	G	6.46	0.43	230	M, C, F, B
	3	245846102	C	T	9.65	0.38	230	M, C, B
	7	160010057	T	G	5.33	0.41	230	M, C, F, B
	7	162215421	G	C	11.13	0.39	230	M, C, F, B
CTTA_2	1	44201188	G	T	6.44	0.22	221	M, C, F,
	1	59238807	G	A	10.66	0.18	221	M, C, F, B
	1	59261500	C	T	5.55	0.19	221	M, C
	1	59765951	C	T	6.51	0.20	221	M, C
	7	160010057	T	G	6.15	0.41	221	M, C, F, B
	7	162215421	G	C	5.79	0.39	221	M, C, B

Table S2.

Model	Intercept a	Slope b	S (RMSE)	R^2 (%)	Approx. 95% PI half-width ≈1.96×S≈1.96
(OLS)	13.97	1.089	116.5	38.3	228.4
RR-BLUP	14.90	1.141	116.6	38.1	228.5
LASSO	14.73	1.130	116.6	37.9	228.5
EN (0.5)	14.94	1.141	116.6	38.1	228.5
RF	13.71	1.225	102.8	51.9	201.6
RKHS	31.11	1.202	110.8	44.2	217.1

Table S3.

Model	N	N_cv	Mean_r2	SD_r^2	Mean_r	sd_r	Mean_rmse	SD_rmse	Mean_slope	SD_slope	Mean_slope0
FEM	24	25	0.27	0.14	0.49	0.17	123.50	19.39	1.04	0.61	1.04
RR-BLUP	24	25	0.27	0.14	0.50	0.17	123.52	20.77	1.14	0.68	1.14
LASSO	24	25	0.27	0.14	0.50	0.17	123.93	20.95	1.13	0.69	1.13
EN(a=0.5)	24	25	0.27	0.14	0.50	0.17	123.80	21.18	1.15	0.70	1.15
RF	24	25	0.27	0.13	0.50	0.16	122.03	21.75	1.12	0.49	1.12
RKHS	25	25	0.27	0.13	0.50	0.14	124.83	21.45	338.13	1683.91	338.13

Table S4.

Model	Intercept a	Slope b	S (RMSE)	R^2 (%)	Approx. 95% PI half-width ≈1.96×S≈1.96
FEM	25.2	1.15	110.6	32.5	206
RR-BLUP	24.6	1.12	109.9	33.4	201
LASSO	25.9	1.18	111.2	31.9	209
EN (0.5)	25.1	1.14	110.3	32.9	204
RF	22.7	1.09	96.4	51.2	181
RKHS	23.9	1.07	95.1	50.2	178

Table S5.

Model	N	N_cv	Mean_r^2	sd_r^2	mean_r	sd_r	mean_rmse	sd_rmse	mean_slope	sd_slope	mean_slope0
FEM	25	25	0.23	0.14	0.45	0.18	120.52	11.93	0.93	0.34	0.93
RR-BLUP	25	25	0.24	0.14	0.46	0.17	119.88	11.60	1.03	0.36	1.03
LASSO	25	25	0.23	0.13	0.45	0.17	120.45	11.52	1.00	0.36	1.00
EN(a=0.5)	25	25	0.24	0.13	0.46	0.17	120.34	11.47	1.02	0.36	1.02
RF	25	25	0.31	0.15	0.54	0.15	117.07	18.10	1.13	0.30	1.13
RKHS	25	25	0.27	0.14	0.50	0.15	126.55	16.59	1.44	0.48	1.44

Table S6.

KNOU name	Accession name	Commercial name	Lettuce type	Leaf color	Source institution	Country of source	Country of origin
L0001	PI_593431	DOMINGOS 42	CH	G	USDA	USA	USA
L0002	PI_536705	CLIMAX	CH	G	USDA	USA	USA
L0003	PI_600913	FM 8248	CH	G	USDA	USA	USA
L0004	PI_583879	PRIMETIME	CH	G	USDA	USA	USA
L0005	PI_536737	LAKES SHIPPER	CH	G	USDA	USA	USA
L0006	PI_536728	EAT LAKES R-200	CH	G	USDA	USA	USA
L0007	PI_593416	SUPER 59	CH	G	USDA	USA	USA
L0008	PI_601113	CENTURION	CH	G	USDA	USA	USA
L0009	PI_595596	AMARAL 400	CH	G	USDA	USA	USA
L0010	PI_601481	SALVERDE	CH	G	USDA	USA	USA
L0011	PI_561196	IMPACT	CH	G	USDA	USA	AUS
L0012	PI_536741	IMPERIAL 101	CH	G	USDA	USA	USA
L0013	PI_342476-2	Cavolo di Napol	CH	G	USDA	USA	USA
L0014	PI_342476-1	Cavolo di Napol	CH	G	USDA	USA	USA
L0015	PI_358043-1	Zimska zelena	CH	G	USDA	USA	YUG
L0016	PI_536742	IMPERIAL 847	CH	G	USDA	USA	USA
L0017	PI_165492-1	-	CH	G	USDA	USA	USA
L0018	PI_418990-1	-	CH	G	USDA	USA	USA
L0019	PI_379363	Veleska	CH	G	USDA	USA	YUG
L0020	PI_342502-1	a: blonde a bord	CH	G	USDA	USA	USA
L0021	PI_342514-2	gante de Tucum	CH	G	USDA	USA	NLD
L0022	PI_342514-1	gante de Tucum	CH	G	USDA	USA	NLD
L0023	PI_342514-4	gante de Tucum	CH	G	USDA	USA	NLD
L0024	PI_342504-2*	Gloire de Nantes	CH	G	USDA	USA	USA
L0025	PI_342504-1	Gloire de Nantes	CH	G	USDA	USA	USA
L0026	PI_342503-3	avia: blonde de F	CH	G	USDA	USA	USA
L0027	PI_342503-1	avia: blonde de F	CH	G	USDA	USA	USA
L0028	PI_543962	EASTERN LAKES	CH	G	USDA	USA	USA
L0029	PI_491078	-	LF	G	USDA	USA	USA
L0030	PI_491062	-	LF	G	USDA	USA	USA

L0031	PI_164937	-	LF	G	USDA	USA	USA
L0032	PI_491070	-	LF	G	USDA	USA	USA
L0033	PI_344366-1	Kivincik Morul	LF	G	USDA	USA	TUR
L0034	PI_177419	-	LF	G	USDA	USA	USA
L0035	PI_175736	-	LF	G	USDA	USA	USA
L0036	PI_146078-7*	-	LF	G	USDA	USA	USA
L0037	PI_146078-1	-	LF	G	USDA	USA	USA
L0038	PI_491080	-	LF	G	USDA	USA	USA
L0039	PI_491008	-	RM	G	USDA	USA	USA
L0040	PI_536700	CK-SEEDED SIMP	LF	G	USDA	USA	USA
L0041	PI_289016-2*	ericanischer Brau	LF	R	USDA	USA	HUN
L0042	PI_271476-1	-	LF	R	USDA	USA	USA
L0043	PI_578884	RED GIANT	LF	R	USDA	USA	NLD
L0044	PI_601060	RED SAILS	LF	R	USDA	USA	USA
L0045	PI_507926-3	-	CH	R	USDA	USA	USA
L0046	PI_507928-1	-	CH	G	USDA	USA	USA
L0047	PI_491031	-	RM	G	USDA	USA	USA
L0048	PI_491056	-	RM	G	USDA	USA	USA
L0049	PI_176980	Chbekli	RM	G	USDA	USA	USA
L0050	PI_169509-1	-	RM	G	USDA	USA	USA
L0051	PI_169493	Marul	RM	G	USDA	USA	USA
L0052	PI_278070-1	-	RM	G	USDA	USA	USA
L0053	PI_491025	-	RM	G	USDA	USA	USA
L0054	PI_491039	-	RM	G	USDA	USA	USA
L0055	PI_278072	-	RM	G	USDA	USA	USA
L0056	PI_536794	TRIANON COS	RM	G	USDA	USA	USA
L0057	PI_176579-2	-	RM	G	USDA	USA	USA
L0058	PI_491045	-	RM	G	USDA	USA	USA
L0059	PI_358039-1	Crna marula	RM	G	USDA	USA	YUG
L0060	PI_172918	-	RM	G	USDA	USA	USA
L0061	PI_182209-1	-	RM	G	USDA	USA	USA
L0062	PI_171665-1	Pevrek	RM	G	USDA	USA	USA
L0063	PI_368630-1*	Crna	RM	G	USDA	USA	YUG
L0064	PI_278099-1	-	RM	G	USDA	USA	USA

L0065	PI_491053	-	RM	G	USDA	USA	USA
L0066	PI_278081-1	-	RM	G	USDA	USA	USA
L0067	PI_491049	-	RM	G	USDA	USA	USA
L0068	PI_288244-3	Balady	RM	G	USDA	USA	EGY
L0069	PI_491040	-	RM	G	USDA	USA	USA
L0070	PI_577120	ROMANA VERDE	RM	G	USDA	USA	USA
L0071	PI_491034	-	RM	G	USDA	USA	USA
L0072	PI_491213*	-	RM	G	USDA	USA	USA
L0073	PI_491212	-	RM	G	USDA	USA	USA
L0074	PI_220665-1	Kahoo	RM	G	USDA	USA	USA
L0075	PI_278108-1	-	RM	G	USDA	USA	USA
L0076	PI_140394	-	RM	G	USDA	USA	USA
L0077	PI_342541-4	allone o Bougiva	RM	G	USDA	USA	NLD
L0078	PI_171666	Siyqh	RM	G	USDA	USA	USA
L0079	PI_491022	-	RM	G	USDA	USA	USA
L0080	PI_176588	Adi	RM	G	USDA	USA	USA
L0081	PI_285657	Rakowicka	RM	G	USDA	USA	HUN
L0082	PI_251501	Kahu	ST	G	USDA	USA	IRN
L0083	PI_221936-1	Kahoo	ST	G	USDA	USA	USA
L0084	PI_220524*	Kahoo	ST	G	USDA	USA	USA
L0085	PI_173909	Kahu	ST	G	USDA	USA	USA
L0086	PI_536777	URPEES CELTUC	ST	G	USDA	USA	USA
L0087	PI_391601-1	Chi-kua-sun	ST	G	USDA	USA	CHN
L0088	PI_342524-1*	Native	LF	G	USDA	USA	NLD
L0089	PI_342523-1	Fi Native	LF	G	USDA	USA	NLD
L0090	PI_278094	-	RM	G	USDA	USA	USA
L0091	PI_169494	-	LF	G	USDA	USA	USA
L0092	PI_615060-1	Ziegler's Heirloon	LF	G	USDA	USA	DEU
L0093	PI_342474-1	ccola a foglie lisc	LF	G	USDA	USA	USA
L0094	PI_536821	FORDHOOK	RM	G	USDA	USA	USA
L0095	PI_342552-1	Galiega driver	RM	G	USDA	USA	NLD
L0096	PI_381933*	de Invierno (sele	RM	G	USDA	USA	ARG
L0097	PI_381932-1	de Invierno (sele	BH	G	USDA	USA	ARG
L0098	PI_342555-2*	Madrilene	BH	G	USDA	USA	NLD

L0099	PI_342555-1	Madrilene	BH	G	USDA	USA	NLD
L0100	PI_289018	ohlblattriger Butt	BH	G	USDA	USA	HUN
L0101	PI_617943-1	Lolla Rossa	LF	R	USDA	USA	USA
L0102	PI_536699	K-SEEDED BATH	LF	G	USDA	USA	USA
L0103	PI_278109-1	-	LF	G	USDA	USA	USA
L0104	PI_344367-1	-	LF	G	USDA	USA	USA
L0105	PI_278080-2	-	LF	G	USDA	USA	USA
L0106	PI_536826	DARK GRN GR	LF	G	USDA	USA	USA
L0107	PI_508477-1	Seoul Green	LF	G	USDA	USA	KOR
L0108	PI_212099	-	LF	G	USDA	USA	USA
L0109	PI_176578*	-	LF	G	USDA	USA	USA
L0110	PI_175737	-	LF	G	USDA	USA	USA
L0111	PI_172916-1	Gobekli	LF	G	USDA	USA	USA
L0112	PI_358021	Kadrava	LF	G	USDA	USA	YUG
L0113	PI_491079	-	LF	G	USDA	USA	USA
L0114	PI_358001-1	Strumicka	LF	G	USDA	USA	YUG
L0115	PI_491065	-	LF	G	USDA	USA	USA
L0116	PI_617958-1	Misticanza	LF	R	USDA	USA	USA
L0117	PI_182210-1	-	LF	G	USDA	USA	USA
L0118	PI_342528-2	oeftuins Blackpo	BH	G	USDA	USA	NLD
L0119	PI_342517-1	Ancora	BH	G	USDA	USA	NLD
L0120	PI_342532-1	Korrekt	BH	G	USDA	USA	NLD
L0121	PI_342525-1	ivo (sel. uit IVT 5	BH	G	USDA	USA	NLD
L0122	PI_342533-1	Kwadraat	BH	G	USDA	USA	NLD
L0123	PI_342531-1	Kordaat	BH	G	USDA	USA	NLD
L0124	PI_342445-1	Regina	BH	G	USDA	USA	NLD
L0125	W6_29842	-	BH	G	USDA	USA	USA
L0126	PI_342457-1	Amplus '63'	BH	G	USDA	USA	NLD
L0127	PI_342449-1	Kolos	BH	G	USDA	USA	NLD
L0128	PI_342460-1	saro (sel. uit IVT	BH	G	USDA	USA	NLD
L0129	PI_342458-1	Iveto	BH	G	USDA	USA	NLD
L0130	PI_342456-1	Atlas	BH	G	USDA	USA	NLD
L0131	PI_342459-3	Akcel	BH	G	USDA	USA	NLD
L0132	PI_342455-1	Progo (No. 39)	BH	G	USDA	USA	USA

L0133	PI_342487-1	ion Blanche a gr	BH	G	USDA	USA	USA
L0134	PI_373914	Rapide	BH	G	USDA	USA	NLD
L0135	PI_503631	Start	BH	G	USDA	USA	DEU
L0136	PI_503622	Muck	BH	G	USDA	USA	DEU
L0137	PI_342464-1	Bonus	BH	G	USDA	USA	NLD
L0138	PI_372907	Amanda	BH	G	USDA	USA	NLD
L0139	PI_342468-1	Kloek	BH	G	USDA	USA	NLD
L0140	PI_342518-1	Doppio	BH	G	USDA	USA	NLD
L0141	PI_289025-1	rinz v. Lowenstei	BH	G	USDA	USA	HUN
L0142	PI_342512-1	Reine de Mai	BH	G	USDA	USA	USA
L0143	PI_342490-1	ai de pleine terr	BH	G	USDA	USA	USA
L0144	PI_503642	CSSR 1981:81	BH	G	USDA	USA	SUN
L0145	PI_342467-1*	No. 1049	BH	G	USDA	USA	USA
L0146	PI_342462-1	Hivera-winter	BH	G	USDA	USA	NLD
L0147	PI_342446-1	Plenos	BH	G	USDA	USA	NLD
L0148	PI_342486-1	Princeps Treib	BH	G	USDA	USA	USA
L0149	PI_273606	Treibsalat Presto	BH	G	USDA	USA	USA
L0150	PI_503617	Ioffmanns Auror	BH	G	USDA	USA	DEU
L0151	PI_342500-1	Premice	BH	G	USDA	USA	USA
L0152	PI_503618	Kison	BH	G	USDA	USA	DEU
L0153	PI_342465-1	Silva	BH	G	USDA	USA	NLD
L0154	PI_342463-2	altura (v/h Kosur	BH	G	USDA	USA	NLD
L0155	PI_342509-1	sion blonde a gr	BH	G	USDA	USA	USA
L0156	PI_342510-1	te d'hiver de Cha	BH	G	USDA	USA	USA
L0157	PI_342493-1	Brune d'hiver	BH	R	USDA	USA	USA
L0158	PI_342557-1	sion blanche a g	BH	G	USDA	USA	NLD
L0159	PI_379357	Glavesta	BH	G	USDA	USA	YUG
L0160	PI_271937-1*	-	BH	G	USDA	USA	USA
L0161	PI_264335	Col de Napoles	BH	G	USDA	USA	ESP
L0162	PI_264671	erkopfsalat Maiw	BH	G	USDA	USA	DEU
L0163	PI_507930-1	-	BH	G	USDA	USA	USA
L0164	PI_273612	-	BH	G	USDA	USA	USA
L0165	PI_503605-1	Brauner Winter	BH	G	USDA	USA	DEU
L0166	PI_370472-1	Puter	BH	R	USDA	USA	YUG

L0167	PI_503641	ditalien 1981:74	BH	R	USDA	USA	ITA
L0168	PI_273205-1	-	BH	G	USDA	USA	USA
L0169	PI_342448-1	rosse brune tetu	BH	R	USDA	USA	NLD
L0170	PI_342558-2*	Blonde de Prieur	BH	G	USDA	USA	NLD
L0171	W6_29814-1	-	BH	G	USDA	USA	USA
L0172	PI_342473-2*	Attrazione	BH	G	USDA	USA	USA
L0173	PI_342470-1	Bohemia	BH	G	USDA	USA	NLD
L0174	PI_342507-1	Lorthois	BH	G	USDA	USA	USA
L0175	PI_342485-4	AWu	BH	G	USDA	USA	USA
L0176	PI_342440-3	Ramses	BH	G	USDA	USA	NLD
L0177	PI_342554-2	Lilloise	BH	G	USDA	USA	NLD
L0178	PI_342554-1	Lilloise	BH	G	USDA	USA	NLD
L0179	PI_385971-2*	ll The Year Roun	BH	G	USDA	USA	KEN
L0180	PI_385971-1	ll The Year Roun	BH	G	USDA	USA	KEN
L0181	PI_342540-1	ocadero La Prefe	BH	G	USDA	USA	NLD
L0182	PI_390975-1	Rinat Hokfar	BH	G	USDA	USA	ISR
L0183	PI_289026-3	Rudolfs Liebling	BH	G	USDA	USA	HUN
L0184	PI_342498-3	Gloire de Nantes	BH	G	USDA	USA	USA
L0185	PI_250429	Stupicky	BH	G	USDA	USA	CSK
L0186	W6_29844	-	BH	G	USDA	USA	USA
L0187	IT_100511	뚝섬적축면상추	LF	R	ASIA SEED	KOR	CHN
L0188	IT_228745	그랜드래피드 TB	LF	G	ASIA SEED	KOR	USA
L0189	IT_363750	제일진홍적축면	LF	R	Jeil Seed	KOR	KOR
L0190	IT_328525	권농포찹이상추	LF	R	WONNONG SEE	KOR	USA
L0191	-	토종맛적축면	LF	R	WONNONG SEE	KOR	USA
L0192	-	정통포기상추	LF	R	Farm Hannong	KOR	CHN
L0193	-	적하계	LF	R	RDA	KOR	KOR
L0194	IT_363749	여름적치마상추	LF	R	ASIA SEED	KOR	CHN
L0195	-	레드원상추	LF	R	WONNONG SEE	KOR	USA
L0196	IT_100513	적치마상추	LF	R	SAKATA KOREA	KOR	USA
L0197	IT_300154	수라적치마	LF	R	Samsung Seed	KOR	CHN
L0198	-	크레드맛있는상	LF	R	Samsung Seed	KOR	CHN
L0199	-	농적치마	LF	R	Syngenta Korea	KOR	USA
L0200	-	혜선적치마	LF	R	RDA	KOR	KOR

L0201	-	여름아삭이생채	LF	G	Jeil Seed	KOR	CHN
L0202	-	제일청축면상추	LF	G	Jeil Seed	KOR	ITA
L0203	-	하청-1	LF	G	RDA	KOR	KOR
L0204	IT_100512	청치마상추	LF	G	ASIA SEED	KOR	CHN
L0205	-	고향담배상추	LF	G	Jeil Seed	KOR	CHN
L0206	-	청일점상추	LF	G	Jeil Seed	KOR	CHN
L0207	IT_363756	청품여름치마상추	LF	G	WONNONG SEE	KOR	USA
L0208	-	골드그린상추	LF	G	WONNONG SEE	KOR	USA
L0209	-	어울림청치마	LF	G	Samsung Seed	KOR	CHN
L0210	-	스노우그린	LF	G	Ggdares	KOR	KOR
L0211	-	경기가-6호	LF	G	Ggdares	KOR	KOR
L0212	-	흑쌈치마상추	LF	R	ASIA SEED	KOR	USA
L0213	-	자바흑치마상추	LF	R	WONNONG SEE	KOR	USA
L0214	-	삼복먹치마	LF	R	WONNONG SEE	KOR	USA
L0215	-	먹돌이상추	LF	R	Syngenta Korea	KOR	USA
L0216	IT_363731	아이스퀸상추	CH	G	ASIA SEED	KOR	ITA
L0217	-	쓰리랑양상추	CH	G	ASIA SEED	KOR	CHN
L0218	-	상추.생채	CH	G	ASIA SEED	KOR	USA
L0219	-	홍쌈로메인	RM	R	ASIA SEED	KOR	USA
L0220	-	슈퍼시저스레드	RM	R	ASIA SEED	KOR	CHN
L0221	IT_290072	Zijing	ST	G	RDA	KOR	CHN
L0222	IT_277867	Kafei han baoluc	ST	G	RDA	KOR	CHN
L0223	-	오크린상추	Oakleaf	G	ASIA SEED	KOR	ITA
L0224	-	오크린상추_1	Oakleaf	G	RDA	KOR	ITA
L0225	-	Rafeto Red	LF	R	RDA	KOR	KOR
L0226	-	횡성적상추	LF	R	Ggdares	KOR	KOR
L0227	-	적배추상추	LF	R	Ggdares	KOR	KOR
L0228	-	제일알로에상추	LF	G	Jeil Seed	KOR	CHN
L0229	IT_363723	그린생채	LF	G	AGRC	KOR	JPN
L0230	-	햇살적축면	LF	R	Ggdares	KOR	KOR
L0231	IT_195215	토종부루	LF	R	Ggdares	KOR	KOR
L0232	-	유리생채	LF	G	RDA	KOR	USA
L0233	-	아리랑 양상추	LF	G	RDA	KOR	KOR
L0234	-	담배상추-에쎄	LF	G	RDA	KOR	KOR

L0235	-	밸러드 익스프레스	LF	G	RDA	KOR	KOR
L0236	-	아시아모둠상추	LF	R	RDA	KOR	KOR
L0237	-	홍아적축면상추	LF	R	RDA	KOR	KOR
L0238	IT_363733	청하청치마	LF	G	RDA	KOR	KOR
L0239	IT_100512	청치마상추-1	LF	G	RDA	KOR	CHN
L0240	-	시아여름적치마상	LF	R	RDA	KOR	KOR
L0241	IT_363732	진빨롤라상추	LF	R	RDA	KOR	USA
L0242	-	슈러시저드레드	LF	R	RDA	KOR	KOR
L0243	-	아바타	LF	R	RDA	KOR	KOR
L0244	-	여름로메인	LF	G	RDA	KOR	CHN
L0245	IT_363740	여름청치마상추	LF	G	RDA	KOR	CHN
L0246	-	흑치마상추	LF	R	Jeil Seed	KOR	CHN
L0247	IT_363740	여름적치마상추	LF	R	ASIA SEED	KOR	CHN
L0248	-	여름진홍적축면	LF	R	RDA	KOR	KOR
L0249	-	여름개량담배상추	LF	G	RDA	KOR	KOR
L0250	-	제일생채상추	LF	G	RDA	KOR	USA
L0251	-	로메레기나상추	LF	G	RDA	KOR	KOR
L0252	-	청일점상추	LF	G	Jeil Seed	KOR	CHN
L0253	-	웰빙참맛상추	LF	G	Jeil Seed	KOR	CHN
L0254	-	진아로에상추	LF	G	RDA	KOR	KOR
L0255	IT_231530	아삭아삭생채상추	LF	G	RDA	KOR	USA
L0256	-	오크리프상추	LF	G	RDA	KOR	KOR
L0257	IT_228753	삼선적축면상추	LF	R	Farm Hannong	KOR	CHL
L0258	-	파워선적축면상추	LF	R	RDA	KOR	KOR
L0259	IT_231530	열풍 적치마	LF	R	RDA	KOR	KOR
L0260	PI_617952	La Brillante	LF	G	USDA	USA	USA
L0261	PI_617959	Little Gem	LF	G	USDA	USA	USA
L0262	PI_536849	ARNWOOD GEN	LF	G	USDA	USA	USA
L0263	PI_613577	ERUPTION	LF	G	USDA	USA	USA
L0264	PI_667691	Defender	LF	G	USDA	USA	USA
L0265	PI_667699	Infantry	LF	R	USDA	USA	USA
L0266	PI_667702	Merlot	LF	R	USDA	USA	USA
L0267	PI_667705	Pavane	LF	G	USDA	USA	USA
L0268	PI_667707	Sucrine	LF	G	USDA	USA	USA

L0269	-	퍼원레드로메인	LF	R	RDA	KOR	KOR
L0270	-	청솔담배	LF	G	RDA	KOR	KOR
L0271	-	오크린상추	LF	G	ASIA SEED	KOR	ITA
L0272	-	상추아시아적오크	LF	R	RDA	KOR	KOR
L0273	-	상추적생채	LF	R	RDA	KOR	KOR
L0274	-	불꽃상추	LF	R	RDA	KOR	KOR
L0275	-	진빨상추	LF	R	RDA	KOR	KOR
L0276	IT_363762	홈런왕	LF	R	RDA	KOR	KOR
L0277	IT_231531	미풍포참	LF	R	RDA	KOR	KOR
L0278	-	미래웰빙담배	LF	G	RDA	KOR	KOR
L0279	-	결구양상추	LF	G	RDA	KOR	KOR
L0280	-	삼계청치마	LF	G	RDA	KOR	CHN
L0281	-	유-레이크 상추	LF	G	RDA	KOR	KOR
L0282	-	청치마	LF	G	ASIA SEED	KOR	CHN
L0283	IT_178434	장수	LF	R	RDA	KOR	KOR
L0284	-	만상	LF	G	RDA	KOR	KOR
L0285	-	썬레드버터	LF	R	RDA	KOR	KOR
L0286	IT_231532	선풍	LF	R	RDA	KOR	KOR
L0287	-	만상	LF	G	RDA	KOR	KOR
L0288	-	하청-2	LF	G	RDA	KOR	KOR

Table S7.

No.	Chr1_59238807	Chr2_196799584	Chr4_71593965	Chr5_59213356	Chr5_80193683	Chr7_98088697	Chr7_160010057	Chr7_162215421	Chr7_162466345	CTTB_BLUP
1	1	1	1	1	-1	1	1	1	1	5.9788442
2	1	1	1	1	-1	1	1	1	1	51.303645
3	1	1	1	1	-1	1	1	1	1	74.861149
4	1	1	1	1	-1	1	1	1	1	202.90964
5	1	1	1	1	-1	1	1	1	1	58.867745
6	1	1	1	1	-1	1	1	1	0	170.07227
7	1	1	1	1	-1	1	1	1	1	135.69739
8	1	1	1	-1	1	1	1	1	1	85.407722
9	1	-1	1	-1	-1	1	1	1	1	57.024769
10	1	1	1	1	-1	1	1	1	1	127.43748
11	1	1	1	1	-1	1	1	1	1	203.17132
12	1	1	1	1	-1	1	1	1	1	65.720456
13	1	1	1	-1	-1	1	1	1	1	15.209813
14	1	1	1	-1	-1	1	1	1	1	37.873891
15	1	1	1	1	-1	1	1	1	1	66.537766
16	1	1	1	1	-1	1	1	1	1	51.303645
17	1	-1	1	1	-1	1	1	1	1	37.726664
18	1	1	1	1	-1	1	1	1	1	24.395733
19	1	1	1	1	-1	1	1	1	1	-49.919681
20	1	1	1	1	-1	1	1	1	1	37.504813
21	1	1	1	1	-1	1	1	1	1	-10.776161
22	1	1	1	1	-1	1	1	1	1	39.318365
23	1	1	1	1	-1	1	1	1	1	50.920782
24	1	1	1	1	-1	1	1	1	1	70.174874
25	1	0	1	1	-1	1	1	1	1	39.109467
26	0	1	1	1	-1	1	0	1	1	136.51441

27	1	1	1	1	-1	1	1	1	1	99.362342
28	1	1	1	1	1	1	1	1	1	43.537812
29	0	1	-1	-1	-1	-1	-1	-1	-1	-179.10608
30	-1	1	-1	-1	-1	1	-1	-1	-1	-173.919
31	1	1	1	-1	-1	-1	-1	-1	-1	-210.05316
32	-1	1	1	0	-1	1	-1	-1	-1	-260.6145
33	1	-1	1	0	-1	1	1	1	-1	-136.55371
34	0	0	1	0	-1	1	1	-1	-1	-176.76862
35	-1	1	-1	1	-1	-1	-1	-1	0	-151.50212
36	1	1	1	1	-1	1	1	1	1	23.602504
37	1	1	1	1	N	1	1	1	1	43.706233
38	-1	1	-1	1	-1	-1	-1	1	1	-252.83635
39	-1	1	1	0	-1	1	1	1	1	-252.43815
40	1	1	1	1	-1	1	1	1	1	-185.23647
41	1	-1	1	1	1	1	1	1	1	30.108215
42	1	1	1	1	1	1	1	1	1	2.2635017
43	1	1	1	1	1	1	1	1	1	104.29762
44	1	-1	1	1	1	1	1	1	1	65.720456
45	0	1	1	-1	-1	1	1	1	1	8.0615469
46	1	1	1	-1	-1	1	1	1	1	15.586896
47	-1	1	1	1	-1	1	1	1	1	-124.2309
48	-1	1	1	1	-1	1	1	1	1	-53.414165
49	-1	1	1	1	-1	1	1	1	0	18.79392
50	-1	1	1	1	-1	1	-1	-1	-1	-199.43619
51	-1	1	1	1	-1	1	-1	-1	-1	-179.86517
52	1	1	1	1	-1	1	-1	-1	0	-157.25719
53	-1	1	-1	-1	-1	1	1	1	1	-226.62794
54	1	1	1	0	-1	1	1	1	1	-13.086292
55	1	1	1	1	-1	1	-1	1	0	-117.0223

56	1	1	1	1	-1	1	1	1	1	16.242154
57	-1	1	1	1	-1	1	-1	-1	-1	-197.54492
58	-1	1	1	1	-1	1	1	1	1	56.774919
59	1	1	1	1	-1	1	1	1	1	8.379044
60	1	1	1	1	-1	1	0	-1	-1	-75.689411
61	0	1	-1	-1	-1	-1	1	1	1	-85.269983
62	1	1	1	1	-1	-1	-1	-1	-1	-223.92611
63	1	1	1	1	-1	1	1	1	0	21.121001
64	1	0	1	1	-1	0	1	1	1	69.886847
65	1	1	1	-1	-1	1	1	1	1	-180.60207
66	1	1	1	-1	-1	1	0	0	0	-42.782134
67	0	1	1	-1	-1	1	1	1	1	-88.264538
68	1	1	1	-1	-1	1	1	1	1	-105.43804
69	1	1	1	-1	-1	1	-1	1	1	-99.005826
70	1	1	1	-1	-1	1	1	-1	-1	-242.98073
71	-1	1	1	-1	-1	1	1	1	1	-117.39917
72	1	1	1	-1	-1	1	1	1	1	-73.597693
73	1	1	1	-1	-1	1	1	1	1	-108.29596
74	1	1	1	-1	-1	1	1	1	1	-124.80915
75	1	1	1	-1	-1	-1	1	1	1	-80.223017
76	1	1	1	-1	-1	1	-1	-1	-1	-112.44274
77	-1	1	1	-1	1	1	1	1	1	107.3245
78	-1	1	1	-1	-1	1	1	1	1	-131.15678
79	-1	1	1	1	-1	-1	1	1	1	-79.912888
80	1	1	-1	-1	-1	1	-1	1	1	-149.36474
81	1	1	1	-1	-1	1	-1	-1	-1	-4.1920359
82	1	1	-1	1	-1	-1	1	1	1	-134.0584
83	1	1	-1	-1	-1	-1	1	-1	0	-161.20575
84	1	1	-1	-1	-1	-1	1	-1	0	-174.22463

85	1	1	-1	-1	-1	-1	1	-1	1	-155.16459
86	1	1	1	-1	1	1	-1	-1	-1	-140.69691
87	1	1	1	-1	-1	-1	-1	-1	0	-106.92575
88	-1	1	1	-1	-1	-1	1	-1	-1	-236.92558
89	-1	1	1	-1	-1	-1	1	-1	-1	-236.92558
90	-1	1	1	-1	-1	1	-1	-1	-1	-235.00937
91	-1	1	-1	-1	-1	1	-1	-1	1	-242.05666
92	1	1	1	-1	1	1	-1	-1	-1	-183.66729
93	1	1	0	-1	-1	1	-1	-1	-1	-228.51693
94	1	1	1	1	-1	1	-1	-1	-1	-11.440017
95	1	1	1	1	-1	1	-1	-1	-1	-2.151891
96	1	1	1	1	-1	1	-1	-1	0	-9.2260031
97	1	1	1	1	-1	1	-1	-1	-1	-0.5536543
98	1	1	1	1	N	1	1	1	1	33.230153
99	1	1	1	0	-1	1	1	1	1	14.047085
100	1	1	1	-1	1	N	-1	0	-1	-35.649144
101	1	1	-1	-1	1	1	0	0	1	-29.413204
102	1	1	1	0	-1	1	1	1	1	-113.53219
103	1	1	0	-1	1	1	-1	0	-1	-170.84722
104	1	1	1	-1	N	1	-1	1	-1	-249.64951
105	0	1	1	-1	N	1	-1	-1	1	-335.38643
106	1	1	1	-1	-1	1	-1	1	0	-24.380686
107	1	1	1	-1	-1	1	-1	1	-1	-238.96615
108	-1	1	1	-1	-1	1	-1	0	1	-207.89645
109	-1	-1	1	-1	-1	1	-1	1	1	-181.05313
110	-1	1	1	-1	-1	1	-1	1	1	-198.47266
111	-1	1	1	-1	-1	1	1	1	0	-224.5785
112	1	1	1	-1	-1	1	-1	-1	-1	-128.45111
113	1	1	0	-1	-1	N	-1	-1	-1	-222.8933

114	1	1	1	-1	-1	1	-1	-1	-1	-185.7665
115	1	1	1	1	-1	1	-1	1	0	-161.14247
116	1	1	-1	-1	1	1	-1	1	-1	-202.27919
117	1	0	-1	-1	-1	1	-1	1	1	-320.66038
118	-1	1	1	-1	1	-1	0	-1	-1	-111.41417
119	1	1	1	-1	N	1	-1	-1	-1	-36.990389
120	1	1	1	-1	1	1	-1	0	-1	-126.80538
121	1	0	1	-1	-1	1	-1	1	-1	-15.420617
122	1	1	1	1	1	1	-1	-1	-1	-153.2912
123	1	1	1	-1	-1	1	-1	1	0	-58.261917
124	1	-1	1	-1	-1	1	-1	-1	-1	-54.972275
125	1	1	1	-1	-1	1	-1	-1	-1	69.455354
126	1	1	1	-1	1	1	-1	-1	-1	-20.789226
127	1	1	1	-1	N	1	-1	-1	-1	2.3542335
128	1	1	1	-1	-1	1	-1	-1	-1	14.325324
129	1	1	1	-1	1	1	-1	-1	-1	-50.149309
130	1	1	1	-1	N	N	1	0	-1	-38.295035
131	1	1	1	-1	-1	1	-1	-1	-1	16.38523
132	1	1	1	-1	-1	1	-1	-1	-1	-6.1212993
133	1	1	1	-1	-1	1	-1	-1	-1	27.309354
134	1	1	1	-1	-1	1	-1	-1	-1	-59.931374
135	1	1	1	-1	-1	1	-1	-1	-1	-121.73551
136	1	1	1	-1	1	1	-1	-1	-1	-121.2197
137	1	1	1	-1	1	1	-1	-1	-1	-85.525523
138	1	1	1	-1	1	1	-1	-1	-1	-58.833123
139	1	1	1	-1	-1	1	-1	-1	-1	-103.54756
140	1	1	1	-1	-1	1	-1	-1	-1	-119.71269
141	-1	1	1	-1	-1	1	-1	-1	-1	-20.256851
142	1	1	1	-1	1	1	-1	-1	-1	11.133472

143	1	1	1	-1	1	-1	-1	-1	0	3.7273394
144	1	1	1	-1	N	1	-1	-1	-1	-7.3312818
145	1	1	1	-1	1	1	-1	-1	-1	-57.299935
146	1	1	1	-1	1	1	-1	-1	-1	-117.13853
147	1	1	1	1	-1	1	1	1	1	-57.773174
148	1	1	1	-1	1	1	-1	-1	-1	-24.03291
149	1	1	1	-1	-1	1	-1	-1	-1	-64.05274
150	1	1	1	-1	-1	1	-1	-1	-1	-41.10823
151	1	1	1	-1	-1	1	-1	-1	-1	-53.287338
152	1	1	1	-1	-1	1	-1	-1	-1	-41.95315
153	1	1	1	-1	1	1	-1	1	-1	-24.464776
154	1	1	1	-1	1	1	-1	1	-1	50.006314
155	1	1	1	-1	-1	1	1	-1	-1	28.564093
156	1	1	1	-1	N	1	1	-1	-1	-22.341983
157	1	1	1	-1	-1	1	1	-1	-1	-113.52849
158	1	1	1	-1	-1	1	-1	-1	-1	-23.258773
159	1	1	1	-1	-1	1	-1	-1	-1	13.192164
160	1	0	1	1	1	1	-1	-1	-1	-93.345752
161	1	1	1	1	1	1	-1	-1	-1	-89.777677
162	1	1	1	1	1	1	1	1	1	-74.498974
163	1	1	1	-1	-1	1	-1	1	1	-29.737742
164	1	1	1	0	-1	1	1	1	1	-94.434226
165	1	1	1	-1	-1	1	-1	-1	-1	-54.384626
166	1	1	1	-1	-1	1	-1	1	1	-99.903316
167	1	1	1	-1	N	1	1	1	1	-90.24801
168	1	1	1	-1	-1	1	1	1	1	69.136363
169	-1	1	1	-1	-1	1	1	1	1	13.408045
170	1	1	1	-1	-1	1	1	1	1	-14.142182
171	1	1	1	-1	-1	1	1	1	1	-41.440002

172	1	1	1	-1	1	1	1	1	1	16.701795
173	1	1	1	-1	1	1	1	1	1	-3.1831053
174	1	1	1	-1	N	N	1	1	1	0.6522608
175	1	1	1	-1	-1	1	1	1	1	-2.7729041
176	1	1	1	-1	-1	1	1	1	1	-6.8539252
177	1	1	1	-1	1	1	1	1	1	38.723753
178	1	1	1	-1	1	1	1	1	1	-114.45792
179	1	1	1	-1	-1	1	1	1	1	-17.472022
180	1	1	1	-1	-1	1	1	1	1	-49.786695
181	1	1	1	-1	-1	1	-1	1	1	-211.37016
182	1	1	1	-1	-1	1	1	1	1	-37.03846
183	1	1	1	-1	N	N	1	-1	1	-45.973222
184	1	1	1	-1	-1	1	1	1	1	-16.144987
185	1	-1	1	-1	1	1	-1	-1	-1	155.02948
186	1	1	1	1	1	1	1	1	1	71.00475
187	1	1	1	-1	-1	1	1	1	1	-45.114082
188	1	0	1	-1	-1	1	1	1	1	31.293265
189	1	1	1	0	N	1	1	1	1	112.19323
190	1	1	1	-1	N	1	-1	-1	1	123.15778
191	1	1	1	-1	N	1	1	0	1	-62.512364
192	1	1	1	-1	-1	1	0	0	1	177.55971
193	1	1	1	-1	-1	1	1	1	-1	74.607612
194	1	1	1	-1	1	1	1	1	1	280.29337
195	1	1	1	1	-1	1	1	1	1	18.483091
196	1	1	1	-1	1	1	1	1	1	19.459843
197	1	1	1	1	-1	1	1	1	1	0.7063033
198	1	1	1	0	1	1	1	1	1	321.07418
199	1	1	1	-1	1	1	1	1	1	-17.994396
200	1	1	1	-1	1	1	1	1	1	333.5464

201	1	1	1	1	-1	1	1	1	1	316.94584
202	1	1	1	1	-1	1	1	1	1	214.78563
203	1	1	1	1	-1	1	1	1	1	31.492622
204	1	1	1	-1	-1	1	1	-1	-1	168.4752
205	1	1	1	-1	-1	1	1	1	1	-96.871282
206	1	1	1	-1	1	-1	1	-1	-1	124.97461
207	1	1	1	-1	1	1	1	1	1	463.03283
208	1	1	1	-1	-1	0	-1	1	1	368.88424
209	1	1	1	-1	1	1	1	-1	-1	74.659222
210	1	1	1	1	-1	1	1	1	1	133.28602
211	1	1	1	1	-1	1	1	1	1	404.14374
212	1	1	1	-1	0	1	1	-1	-1	53.460999
213	1	1	1	-1	-1	1	1	1	1	147.60404
214	1	1	1	-1	-1	1	1	0	1	224.60825
215	1	1	1	-1	1	1	1	1	1	221.42125
216	1	1	1	-1	-1	1	1	1	1	157.03694
217	1	1	1	1	N	1	1	1	1	463.03283
218	1	1	1	1	-1	1	1	1	1	391.00581
219	1	1	1	1	-1	1	1	1	1	188.59033
220	1	1	-1	-1	1	1	1	1	1	87.153763
221	1	1	-1	-1	1	1	1	1	1	100.11016
222	1	1	1	-1	-1	1	1	-1	-1	-114.02442
223	1	1	1	-1	-1	1	0	1	1	-6.5030222
224	1	1	1	-1	-1	1	1	1	1	-59.439743
225	1	1	0	0	1	1	1	1	1	86.957927
226	1	1	-1	-1	1	N	1	1	1	-75.789731
227	1	1	-1	-1	1	1	1	1	1	-69.003075
228	-1	1	1	-1	1	1	1	-1	-1	-232.98492
229	1	1	1	1	-1	1	1	1	1	521.62671

230	1	1	1	-1	1	1	1	1	1	463.0591
231	1	1	1	-1	-1	-1	1	-1	-1	-153.66583
232	1	1	1	1	-1	1	1	-1	-1	-233.48798
233	1	1	1	0	1	N	1	1	1	463.03283
234	1	1	1	1	-1	1	1	-1	-1	-58.824763
235	1	1	1	1	1	1	1	1	1	214.7342
236	1	1	1	1	1	1	1	1	1	143.71063
237	1	1	1	-1	1	1	1	1	1	152.25002
238	1	1	1	-1	1	1	1	1	1	99.679154
239	1	1	1	-1	1	-1	1	-1	-1	161.51375
240	1	1	1	1	1	1	1	1	1	127.43725
241	1	-1	-1	-1	1	1	1	1	1	146.57802
242	1	1	0	-1	-1	1	1	1	1	70.9878
243	1	1	1	1	-1	1	1	1	1	-65.114318
244	1	1	1	1	-1	1	1	1	1	105.24741
245	1	1	1	-1	1	1	1	1	1	294.86378
246	1	1	1	-1	-1	-1	1	-1	-1	63.757247
247	1	1	1	-1	-1	1	1	1	1	97.83606
248	1	1	1	-1	1	1	1	1	1	159.80736
249	1	1	1	1	-1	1	1	1	1	241.83848
250	-1	1	1	-1	1	1	1	1	1	-79.488465
251	1	1	1	-1	-1	-1	1	1	1	-0.5529857
252	1	N	1	1	-1	N	1	-1	-1	101.27634
253	1	1	1	1	-1	1	1	1	1	245.72802
254	-1	1	1	-1	1	-1	1	1	1	-127.97639
255	1	1	1	1	-1	1	1	1	1	19.103991
256	1	1	1	-1	-1	1	1	1	1	-18.939156
257	1	1	1	-1	N	1	1	1	1	-66.548874
258	1	1	1	-1	N	1	1	1	1	87.557099

259	1	1	1	-1	-1	1	1	1	1	171.897
260	1	1	1	-1	-1	1	1	1	1	100.83229
261	1	1	1	1	-1	1	1	1	1	51.097664
262	1	1	1	1	-1	1	1	1	1	14.939823
263	1	1	1	-1	-1	1	1	1	1	129.59688
264	1	1	1	1	-1	1	1	1	1	89.048253
265	1	1	1	-1	1	1	1	1	1	70.875295
266	1	1	0	1	1	1	1	1	1	127.27636
267	1	1	1	1	-1	1	1	1	1	-57.597514
268	1	1	1	1	-1	1	1	1	1	6.5045347
269	1	1	-1	1	-1	1	1	1	1	2.3730178
270	1	1	1	-1	-1	1	1	1	-1	-80.303532
271	1	1	1	-1	-1	1	1	1	1	-9.0877199
272	1	1	1	-1	-1	1	1	1	1	30.704256
273	1	1	1	1	-1	1	1	1	1	236.63277
274	1	-1	1	-1	1	1	1	1	1	79.26743
275	1	1	-1	-1	1	1	1	1	1	158.46588
276	1	1	1	-1	-1	1	1	1	1	262.03928
277	1	1	1	-1	1	1	1	1	1	85.685144
278	1	1	1	1	1	1	1	-1	-1	-92.749684
279	1	1	1	1	-1	1	1	1	1	163.18115
280	1	1	1	-1	N	1	1	1	1	294.29156
281	1	1	1	1	-1	1	0	1	1	236.47463
282	1	1	1	-1	1	-1	1	-1	-1	11.463349
283	1	1	1	-1	-1	1	1	1	1	235.3009
284	1	1	1	1	-1	1	1	1	1	74.74865
285	1	1	1	-1	-1	1	1	1	1	29.239582
286	1	1	1	-1	1	1	1	1	1	90.251771
287	1	1	1	1	-1	1	1	1	1	111.52294

288	1	1	1	1	-1	1	1	1	1	232.69172

Table S8.

No.	Chr1_5923880	Chr1_4420116	Chr1_5923882	Chr1_5926156	Chr2_2602429	hr3_24584610	hr7_16001005	hr7_1622154	CTTA_BLUP
1	1	1	1	1	0	1	1	1	129.084899
2	1	1	1	1	1	1	1	1	90.172177
3	1	1	1	1	0	0	1	1	192.297259
4	1	0	1	1	1	1	1	1	192.297259
5	1	1	1	1	0	1	1	1	NA
6	1	1	1	1	1	1	1	1	NA
7	1	1	1	1	0	0	1	1	113.132706
8	1	1	1	1	0	1	1	1	101.392034
9	1	1	1	1	0	-1	1	1	305.496055
10	1	1	1	1	1	1	1	1	71.4462996
11	1	1	1	1	1	1	1	1	NA
12	1	1	1	1	0	1	1	1	-3.9577776
13	1	1	1	1	1	0	1	1	102.179183
14	1	1	1	1	1	1	1	1	165.306533
15	1	1	1	1	1	1	1	1	184.028253
16	1	1	1	1	1	1	1	1	234.369103
17	1	0	1	1	0	0	1	1	164.271673
18	1	1	1	1	1	-1	1	1	102.307343
19	1	1	1	1	0	-1	1	1	-15.575511
20	1	1	1	1	0	0	1	1	-6.8045995
21	1	1	1	1	0	0	1	1	16.6453538
22	1	1	1	1	1	0	1	1	81.1039003
23	1	1	1	1	1	1	1	1	29.8903381
24	1	1	1	1	1	0	1	1	26.3822312
25	1	1	1	1	1	-1	1	1	60.78633
26	0	0	0	1	1	1	0	1	23.5083907
27	1	1	1	1	0	-1	1	1	95.4516975

28	1	1	1	1	0	0	1	1	62.8161716
29	0	1	-1	1	0	-1	-1	-1	-147.70684
30	-1	1	-1	1	0	0	-1	-1	-171.65133
31	1	1	1	1	0	0	-1	-1	-106.30568
32	-1	1	-1	1	0	0	-1	-1	-204.44287
33	1	1	0	1	-1	0	1	1	-129.40922
34	0	1	0	1	1	1	1	-1	-109.31323
35	-1	1	-1	1	0	-1	-1	-1	-157.03426
36	1	1	1	1	0	0	1	1	-16.713249
37	1	1	1	1	0	0	1	1	38.9481492
38	-1	1	-1	-1	1	0	-1	1	-31.964788
39	-1	1	-1	1	-1	0	1	1	-209.13334
40	1	1	1	1	0	0	1	1	-137.04378
41	1	1	1	1	-1	-1	1	1	-23.966888
42	1	1	1	1	0	0	1	1	53.3775036
43	1	1	1	1	-1	0	1	1	24.0270964
44	1	1	1	1	-1	1	1	1	128.216784
45	0	1	0	1	1	-1	1	1	116.772484
46	1	1	1	1	1	0	1	1	36.1527988
47	-1	0	-1	-1	0	0	1	1	-85.218276
48	-1	0	-1	-1	1	0	1	1	-125.30091
49	-1	-1	-1	-1	0	0	1	1	-96.042104
50	-1	-1	-1	-1	-1	-1	-1	-1	-158.35372
51	-1	1	-1	1	0	-1	-1	-1	-203.43217
52	1	1	1	1	1	0	-1	-1	-145.53854
53	-1	0	-1	-1	0	-1	1	1	-186.89764
54	1	1	1	1	0	0	1	1	-115.24497
55	1	0	1	1	0	0	-1	1	-110.47843
56	1	0	1	1	1	0	1	1	37.5264019

57	-1	0	-1	-1	0	-1	-1	-1	-235.22378
58	-1	-1	-1	-1	0	0	1	1	-101.21087
59	1	0	1	1	-1	0	1	1	116.43018
60	1	0	1	1	-1	-1	0	-1	-47.38122
61	0	1	1	1	0	0	1	1	-111.52703
62	1	1	1	1	0	0	-1	-1	-171.18511
63	1	1	1	1	0	1	1	1	-5.4617409
64	1	1	1	1	-1	1	1	1	56.1022116
65	1	0	1	1	0	1	1	1	-181.99426
66	1	1	1	1	1	1	0	0	-107.76147
67	0	1	1	1	0	0	1	1	1.06782038
68	1	1	1	1	0	1	1	1	-139.43702
69	1	1	1	1	0	0	-1	1	-115.51857
70	1	1	1	1	0	-1	1	-1	-199.01232
71	-1	1	-1	-1	0	0	1	1	-139.18412
72	1	1	1	1	0	0	1	1	-90.818825
73	1	1	1	1	-1	-1	1	1	-82.3961
74	1	1	1	1	0	0	1	1	-128.22823
75	1	0	1	1	0	0	1	1	-176.75945
76	1	0	1	1	0	1	-1	-1	-146.32659
77	-1	1	-1	-1	0	0	1	1	182.313316
78	-1	0	-1	1	1	0	1	1	9.3198511
79	-1	-1	-1	-1	0	-1	1	1	-85.867706
80	1	0	1	1	0	1	-1	1	-115.51717
81	1	-1	1	1	0	0	-1	-1	67.2463562
82	1	1	1	1	0	-1	1	1	-52.244431
83	1	0	1	1	0	-1	1	-1	-126.32048
84	1	1	1	1	0	-1	1	-1	-111.17108
85	1	1	1	1	0	0	1	-1	-127.47444

86	1	1	1	1	1	1	-1	-1	7.30721124
87	1	1	1	1	1	1	-1	-1	-0.3208487
88	-1	0	-1	-1	1	0	1	-1	-215.63004
89	-1	0	-1	-1	0	-1	1	-1	-249.52679
90	-1	-1	-1	-1	1	1	-1	-1	-233.52824
91	-1	1	-1	-1	-1	0	-1	-1	-200.54939
92	1	-1	1	1	-1	-1	-1	-1	-122.16757
93	1	1	1	1	0	-1	-1	-1	-174.05234
94	1	0	1	1	0	0	-1	-1	-105.3932
95	1	0	1	1	0	1	-1	-1	-2.4619103
96	1	1	1	1	0	1	-1	-1	2.50872204
97	1	0	1	1	0	1	-1	-1	-9.0485923
98	1	0	1	N	1	1	1	1	112.556613
99	1	1	1	N	0	1	1	1	163.059082
100	1	1	1	N	0	1	-1	0	88.2223153
101	1	1	1	1	1	0	0	0	-51.151628
102	1	1	1	1	0	1	1	1	-104.22752
103	1	1	1	1	1	1	-1	0	-93.08951
104	1	1	1	N	1	0	-1	1	-82.075868
105	0	0	0	1	1	1	-1	-1	-263.27643
106	1	1	1	1	0	0	-1	1	-125.81285
107	1	1	1	1	0	0	-1	1	-130.1259
108	-1	1	-1	1	1	1	-1	0	-108.52901
109	-1	1	-1	1	1	1	-1	1	-98.772904
110	-1	1	-1	N	1	1	-1	1	-100.147
111	-1	1	-1	1	0	1	1	1	-123.749
112	1	1	1	1	0	0	-1	-1	-78.663666
113	1	1	1	1	0	1	-1	-1	-155.08603
114	1	1	1	1	1	1	-1	-1	-165.239

115	1	1	1	1	0	0	-1	1	-144.59026
116	1	1	1	1	1	0	-1	1	-99.214805
117	1	1	1	1	0	0	-1	1	-157.49784
118	-1	1	-1	-1	1	1	0	-1	-140.1874
119	1	1	1	1	0	1	-1	-1	1.87907242
120	1	1	1	1	0	1	-1	0	-23.306487
121	1	1	1	1	0	1	-1	1	-49.741618
122	1	1	1	1	1	1	-1	-1	-90.955408
123	1	1	1	1	0	1	-1	1	-69.308092
124	1	-1	1	1	0	1	-1	-1	29.1697694
125	1	1	1	1	0	1	-1	-1	112.153309
126	1	1	1	1	1	1	-1	-1	0.34071979
127	1	1	1	1	1	1	-1	-1	134.277838
128	1	1	1	1	1	1	-1	-1	111.06596
129	1	1	1	1	0	1	-1	-1	40.9421914
130	1	1	1	N	1	1	1	0	13.8675883
131	1	1	1	1	0	1	-1	-1	106.535977
132	1	1	1	1	0	1	-1	-1	68.4744718
133	1	1	1	1	1	1	-1	-1	106.060776
134	1	1	1	1	1	1	-1	-1	-23.767722
135	1	1	1	1	0	1	-1	-1	-123.24325
136	1	1	1	1	1	1	-1	-1	-45.919204
137	1	1	1	1	0	1	-1	-1	-56.17476
138	1	1	1	1	0	1	-1	-1	49.5069658
139	1	1	1	N	0	1	-1	-1	-51.804412
140	1	1	1	1	0	1	-1	-1	-101.93984
141	-1	0	-1	-1	0	1	-1	-1	-102.03878
142	1	1	1	1	1	1	-1	-1	87.8681644
143	1	1	1	1	1	1	-1	-1	118.33344

144	1	1	1	1	0	1	-1	-1	3.79498418
145	1	1	1	1	0	1	-1	-1	-75.895169
146	1	1	1	1	0	1	-1	-1	-103.36754
147	1	1	1	1	0	1	1	1	-35.223809
148	1	1	1	1	0	1	-1	-1	47.3936035
149	1	1	1	1	0	1	-1	-1	-3.4933344
150	1	1	1	1	0	1	-1	-1	18.940589
151	1	1	1	1	1	1	-1	-1	107.728033
152	1	1	1	1	0	1	-1	-1	130.863599
153	1	1	1	1	0	1	-1	1	68.6449883
154	1	1	1	1	1	1	-1	1	123.438698
155	1	1	1	1	0	1	1	-1	10.2409642
156	1	1	1	1	1	1	1	-1	-64.476301
157	1	1	1	1	1	1	1	-1	-128.99165
158	1	1	1	1	0	1	-1	-1	-39.667644
159	1	1	1	N	0	0	-1	-1	25.3176842
160	1	1	1	1	0	0	-1	-1	-6.2442002
161	1	1	1	1	-1	1	-1	-1	-122.5718
162	1	1	1	1	0	0	1	1	-90.205438
163	1	1	1	1	-1	1	-1	1	-126.31822
164	1	1	1	1	0	1	1	1	-46.180799
165	1	1	1	1	1	1	-1	-1	-42.688257
166	1	1	1	1	0	0	-1	1	-72.440856
167	1	1	1	1	0	1	1	1	-77.515375
168	1	1	1	1	1	1	1	1	168.536448
169	-1	-1	-1	-1	1	1	1	1	39.0150217
170	1	1	1	1	0	0	1	1	-14.620735
171	1	1	1	1	0	0	1	1	-57.20407
172	1	1	1	N	-1	1	1	1	-0.6840295

173	1	1	1	N	0	1	1	1	42.5524506
174	1	1	1	N	0	1	1	1	90.2765913
175	1	1	1	1	0	1	1	1	-26.534041
176	1	1	1	1	-1	1	1	1	69.7940357
177	1	1	1	1	0	1	1	1	81.0633614
178	1	1	1	1	0	1	1	1	62.0479668
179	1	1	1	1	0	1	1	1	-20.715042
180	1	1	1	1	0	0	1	1	-3.7622233
181	1	1	1	1	0	1	-1	1	-206.33817
182	1	1	1	N	1	1	1	1	39.219946
183	1	1	1	1	0	1	1	-1	85.4009489
184	1	1	1	1	-1	1	1	1	2.42292771
185	1	1	1	1	0	1	-1	-1	69.1107055
186	1	1	1	1	1	1	1	1	244.868481
187	1	1	1	1	1	1	1	1	-18.942336
188	1	1	1	1	0	0	1	1	172.537341
189	1	1	1	N	-1	0	1	1	NA
190	1	1	1	N	0	1	-1	-1	346.237012
191	1	1	1	1	0	1	1	0	-2.0463795
192	1	1	1	1	-1	1	0	0	-117.20945
193	1	1	1	1	1	1	1	1	192.165474
194	1	1	1	1	1	-1	1	1	NA
195	1	1	1	1	0	0	1	1	67.5638908
196	1	1	1	1	0	0	1	1	45.5293431
197	1	1	1	1	0	1	1	1	149.497487
198	1	1	1	1	0	1	1	1	NA
199	1	1	1	1	0	-1	1	1	82.3050766
200	1	1	1	1	1	-1	1	1	NA
201	1	0	1	1	1	-1	1	1	NA

202	1	0	1	1	1	0	1	1	NA
203	1	1	1	1	0	1	1	1	192.165474
204	1	1	1	1	1	1	1	-1	NA
205	1	0	1	1	0	-1	1	1	-110.51707
206	1	1	1	1	0	1	1	-1	184.545758
207	1	0	1	1	1	1	1	1	NA
208	1	1	1	1	0	1	-1	1	-117.20945
209	1	1	1	1	1	1	1	-1	NA
210	1	1	1	1	1	1	1	1	NA
211	1	0	1	1	1	1	1	1	NA
212	1	1	1	1	0	1	1	-1	143.311116
213	1	1	1	1	0	1	1	1	89.3110138
214	1	1	1	1	0	1	1	0	NA
215	1	1	1	1	-1	1	1	1	152.089744
216	1	1	1	1	0	1	1	1	247.50781
217	1	1	1	1	1	1	1	1	NA
218	1	0	1	1	1	0	1	1	NA
219	1	1	1	1	0	1	1	1	362.82586
220	1	1	1	1	1	0	1	1	212.243774
221	1	1	1	1	1	-1	1	1	NA
222	1	1	1	1	1	1	1	-1	-110.44454
223	1	1	1	1	0	1	0	1	17.4522801
224	1	1	1	1	0	1	1	1	-128.77589
225	1	1	1	1	0	1	1	1	152.242179
226	1	1	1	1	1	0	1	1	-30.989082
227	1	1	1	1	1	-1	1	1	-39.316478
228	-1	0	-1	-1	0	1	1	-1	-348.0539
229	1	0	1	1	0	0	1	1	NA
230	1	1	1	1	1	1	1	1	NA

231	1	1	1	1	0	-1	1	-1	-63.885641
232	1	1	1	1	1	-1	1	-1	-149.32032
233	1	1	1	1	1	1	1	1	NA
234	1	0	1	1	0	-1	1	-1	-93.519344
235	1	1	1	1	1	0	1	1	NA
236	1	1	1	1	0	1	1	1	219.499809
237	1	1	1	1	0	1	1	1	NA
238	1	-1	1	1	1	-1	1	1	NA
239	1	1	1	1	1	1	1	-1	NA
240	1	1	1	1	1	1	1	1	300.533653
241	1	1	1	1	1	0	1	1	NA
242	1	1	1	1	1	1	1	1	156.378685
243	1	-1	1	1	1	0	1	1	NA
244	1	1	1	1	-1	-1	1	1	154.459101
245	1	0	1	1	1	0	1	1	NA
246	1	-1	1	1	1	1	1	-1	256.538286
247	1	1	1	1	1	1	1	1	NA
248	1	1	1	1	-1	1	1	1	175.584542
249	1	1	1	1	0	1	1	1	372.305987
250	-1	1	-1	-1	1	0	1	1	-38.355309
251	1	1	1	1	0	-1	1	1	16.2327954
252	1	1	1	1	0	1	1	-1	102.95848
253	1	0	1	1	1	0	1	1	NA
254	-1	0	-1	1	1	0	1	1	-137.14644
255	1	1	1	1	1	0	1	1	154.614255
256	1	-1	1	1	0	0	1	1	106.722021
257	1	1	1	1	-1	-1	1	1	-67.650619
258	1	1	1	N	0	0	1	1	NA
259	1	1	1	1	0	-1	1	1	332.682512

260	1	1	1	1	0	1	1	1	NA
261	1	1	1	1	0	1	1	1	177.220592
262	1	1	1	1	1	0	1	1	198.723353
263	1	1	1	1	0	-1	1	1	NA
264	1	1	1	1	1	0	1	1	218.726408
265	1	1	1	1	0	-1	1	1	372.565022
266	1	1	1	1	1	-1	1	1	39.0264726
267	1	1	1	1	1	-1	1	1	81.4375606
268	1	0	1	1	1	0	1	1	169.607043
269	1	0	1	1	1	-1	1	1	96.0233702
270	1	0	1	N	0	0	1	1	-94.418178
271	1	1	1	1	0	-1	1	1	52.3642285
272	1	1	1	1	0	0	1	1	64.0377035
273	1	0	1	1	1	0	1	1	NA
274	1	1	1	1	1	-1	1	1	109.885856
275	1	1	1	1	1	-1	1	1	NA
276	1	1	1	1	1	1	1	1	NA
277	1	1	1	1	0	1	1	1	NA
278	1	0	1	1	0	0	1	-1	-110.38284
279	1	1	1	1	0	1	1	1	253.038423
280	1	1	1	1	0	0	1	1	NA
281	1	1	1	N	1	0	0	1	NA
282	1	1	1	1	1	1	1	-1	NA
283	1	1	1	1	-1	-1	1	1	291.444003
284	1	1	1	1	1	0	1	1	21.9174906
285	1	1	1	1	1	-1	1	1	161.741155
286	1	1	1	1	1	0	1	1	NA
287	1	1	1	1	0	-1	1	1	159.497632
288	1	1	1	1	-1	1	1	1	372.305987

Table S9.

KNOU_II	Chr1_442011	Chr1_592388	Chr1_592388	Chr1_592615	Chr2_260242	Chr2_196799	Chr3_245846	Chr4_715939	Chr5_592133	Chr5_801936	Chr7_980886	Chr7_160010	Chr7_162215	Chr7_162466	CTTB_BLUP	CTTA_BLUP
1	1	1	1	1	0	1	1	1	1	-1	1	1	1	1	5.979	129.1
2	1	1	1	1	1	1	1	1	1	-1	1	1	1	1	51.3	90.17
3	1	1	1	1	0	1	0	1	1	-1	1	1	1	1	74.86	192.3
4	0	1	1	1	1	1	1	1	1	-1	1	1	1	1	202.9	192.3
5	1	1	1	1	0	1	1	1	1	-1	1	1	1	1	58.87	NA
6	1	1	1	1	1	1	1	1	1	-1	1	1	1	0	170.1	NA
7	1	1	1	1	0	1	0	1	1	-1	1	1	1	1	135.7	113.1
8	1	1	1	1	0	1	1	1	-1	1	1	1	1	1	85.41	101.4
9	1	1	1	1	0	-1	-1	1	-1	-1	1	1	1	1	57.02	305.5
10	1	1	1	1	1	1	1	1	1	-1	1	1	1	1	127.4	71.45
11	1	1	1	1	1	1	1	1	1	-1	1	1	1	1	203.2	NA
12	1	1	1	1	0	1	1	1	1	-1	1	1	1	1	65.72	-3.958
13	1	1	1	1	1	1	0	1	-1	-1	1	1	1	1	15.21	102.2
14	1	1	1	1	1	1	1	1	-1	-1	1	1	1	1	37.87	165.3
15	1	1	1	1	1	1	1	1	1	-1	1	1	1	1	66.54	184
16	1	1	1	1	1	1	1	1	1	-1	1	1	1	1	51.3	234.4
17	0	1	1	1	0	-1	0	1	1	-1	1	1	1	1	37.73	164.3
18	1	1	1	1	1	1	-1	1	1	-1	1	1	1	1	24.4	102.3
19	1	1	1	1	0	1	-1	1	1	-1	1	1	1	1	-49.92	-15.58
20	1	1	1	1	0	1	0	1	1	-1	1	1	1	1	37.5	-6.805
21	1	1	1	1	0	1	0	1	1	-1	1	1	1	1	-10.78	16.65
22	1	1	1	1	1	1	0	1	1	-1	1	1	1	1	39.32	81.1
23	1	1	1	1	1	1	1	1	1	-1	1	1	1	1	50.92	29.89
24	1	1	1	1	1	1	0	1	1	-1	1	1	1	1	70.17	26.38
25	1	1	1	1	1	0	-1	1	1	-1	1	1	1	1	39.11	60.79
26	0	0	0	1	1	1	1	1	1	-1	1	0	1	1	136.5	23.51

27	1	1	1	1	0	1	-1	1	1	-1	1	1	1	1	99.36	95.45
28	1	1	1	1	0	1	0	1	1	1	1	1	1	1	43.54	62.82
29	1	0	-1	1	0	1	-1	-1	-1	-1	-1	-1	-1	-1	-179.1	-147.7
30	1	-1	-1	1	0	1	0	-1	-1	-1	1	-1	-1	-1	-173.9	-171.7
31	1	1	1	1	0	1	0	1	-1	-1	-1	-1	-1	-1	-210.1	-106.3
32	1	-1	-1	1	0	1	0	1	0	-1	1	-1	-1	-1	-260.6	-204.4
33	1	1	0	1	-1	-1	0	1	0	-1	1	1	1	-1	-136.6	-129.4
34	1	0	0	1	1	0	1	1	0	-1	1	1	-1	-1	-176.8	-109.3
35	1	-1	-1	1	0	1	-1	-1	1	-1	-1	-1	-1	0	-151.5	-157
36	1	1	1	1	0	1	0	1	1	-1	1	1	1	1	23.6	-16.71
37	1	1	1	1	0	1	0	1	1	N	1	1	1	1	43.71	38.95
38	1	-1	-1	-1	1	1	0	-1	1	-1	-1	-1	1	1	-252.8	-31.96
39	1	-1	-1	1	-1	1	0	1	0	-1	1	1	1	1	-252.4	-209.1
40	1	1	1	1	0	1	0	1	1	-1	1	1	1	1	-185.2	-137
41	1	1	1	1	-1	-1	-1	1	1	1	1	1	1	1	30.11	-23.97
42	1	1	1	1	0	1	0	1	1	1	1	1	1	1	2.264	53.38
43	1	1	1	1	-1	1	0	1	1	1	1	1	1	1	104.3	24.03
44	1	1	1	1	-1	-1	1	1	1	1	1	1	1	1	65.72	128.2
45	1	0	0	1	1	1	-1	1	-1	-1	1	1	1	1	8.062	116.8
46	1	1	1	1	1	1	0	1	-1	-1	1	1	1	1	15.59	36.15
47	0	-1	-1	-1	0	1	0	1	1	-1	1	1	1	1	-124.2	-85.22
48	0	-1	-1	-1	1	1	0	1	1	-1	1	1	1	1	-53.41	-125.3
49	-1	-1	-1	-1	0	1	0	1	1	-1	1	1	1	0	18.79	-96.04
50	-1	-1	-1	-1	-1	1	-1	1	1	-1	1	-1	-1	-1	-199.4	-158.4
51	1	-1	-1	1	0	1	-1	1	1	-1	1	-1	-1	-1	-179.9	-203.4
52	1	1	1	1	1	1	0	1	1	-1	1	-1	-1	0	-157.3	-145.5
53	0	-1	-1	-1	0	1	-1	-1	-1	-1	1	1	1	1	-226.6	-186.9
54	1	1	1	1	0	1	0	1	0	-1	1	1	1	1	-13.09	-115.2
55	0	1	1	1	0	1	0	1	1	-1	1	-1	1	0	-117	-110.5

56	0	1	1	1	1	1	0	1	1	-1	1	1	1	1	16.24	37.53
57	0	-1	-1	-1	0	1	-1	1	1	-1	1	-1	-1	-1	-197.5	-235.2
58	-1	-1	-1	-1	0	1	0	1	1	-1	1	1	1	1	56.77	-101.2
59	0	1	1	1	-1	1	0	1	1	-1	1	1	1	1	8.379	116.4
60	0	1	1	1	-1	1	-1	1	1	-1	1	0	-1	-1	-75.69	-47.38
61	1	0	1	1	0	1	0	-1	-1	-1	-1	1	1	1	-85.27	-111.5
62	1	1	1	1	0	1	0	1	1	-1	-1	-1	-1	-1	-223.9	-171.2
63	1	1	1	1	0	1	1	1	1	-1	1	1	1	0	21.12	-5.462
64	1	1	1	1	-1	0	1	1	1	-1	0	1	1	1	69.89	56.1
65	0	1	1	1	0	1	1	1	-1	-1	1	1	1	1	-180.6	-182
66	1	1	1	1	1	1	1	1	-1	-1	1	0	0	0	-42.78	-107.8
67	1	0	1	1	0	1	0	1	-1	-1	1	1	1	1	-88.26	1.068
68	1	1	1	1	0	1	1	1	-1	-1	1	1	1	1	-105.4	-139.4
69	1	1	1	1	0	1	0	1	-1	-1	1	-1	1	1	-99.01	-115.5
70	1	1	1	1	0	1	-1	1	-1	-1	1	1	-1	-1	-243	-199
71	1	-1	-1	-1	0	1	0	1	-1	-1	1	1	1	1	-117.4	-139.2
72	1	1	1	1	0	1	0	1	-1	-1	1	1	1	1	-73.6	-90.82
73	1	1	1	1	-1	1	-1	1	-1	-1	1	1	1	1	-108.3	-82.4
74	1	1	1	1	0	1	0	1	-1	-1	1	1	1	1	-124.8	-128.2
75	0	1	1	1	0	1	0	1	-1	-1	-1	1	1	1	-80.22	-176.8
76	0	1	1	1	0	1	1	1	-1	-1	1	-1	-1	-1	-112.4	-146.3
77	1	-1	-1	-1	0	1	0	1	-1	1	1	1	1	1	107.3	182.3
78	0	-1	-1	1	1	1	0	1	-1	-1	1	1	1	1	-131.2	9.32
79	-1	-1	-1	-1	0	1	-1	1	1	-1	-1	1	1	1	-79.91	-85.87
80	0	1	1	1	0	1	1	-1	-1	-1	1	-1	1	1	-149.4	-115.5
81	-1	1	1	1	0	1	0	1	-1	-1	1	-1	-1	-1	-4.192	67.25
82	1	1	1	1	0	1	-1	-1	1	-1	-1	1	1	1	-134.1	-52.24
83	0	1	1	1	0	1	-1	-1	-1	-1	-1	1	-1	0	-161.2	-126.3
84	1	1	1	1	0	1	-1	-1	-1	-1	-1	1	-1	0	-174.2	-111.2

85	1	1	1	1	0	1	0	-1	-1	-1	-1	1	-1	1	-155.2	-127.5
86	1	1	1	1	1	1	1	1	-1	1	1	-1	-1	-1	-140.7	7.307
87	1	1	1	1	1	1	1	1	-1	-1	-1	-1	-1	0	-106.9	-0.321
88	0	-1	-1	-1	1	1	0	1	-1	-1	-1	1	-1	-1	-236.9	-215.6
89	0	-1	-1	-1	0	1	-1	1	-1	-1	-1	1	-1	-1	-236.9	-249.5
90	-1	-1	-1	-1	1	1	1	1	-1	-1	1	-1	-1	-1	-235	-233.5
91	1	-1	-1	-1	-1	1	0	-1	-1	-1	1	-1	-1	1	-242.1	-200.5
92	-1	1	1	1	-1	1	-1	1	-1	1	1	-1	-1	-1	-183.7	-122.2
93	1	1	1	1	0	1	-1	0	-1	-1	1	-1	-1	-1	-228.5	-174.1
94	0	1	1	1	0	1	0	1	1	-1	1	-1	-1	-1	-11.44	-105.4
95	0	1	1	1	0	1	1	1	1	-1	1	-1	-1	-1	-2.152	-2.462
96	1	1	1	1	0	1	1	1	1	-1	1	-1	-1	0	-9.226	2.509
97	0	1	1	1	0	1	1	1	1	-1	1	-1	-1	-1	-0.554	-9.049
98	0	1	1	N	1	1	1	1	1	N	1	1	1	1	33.23	112.6
99	1	1	1	N	0	1	1	1	0	-1	1	1	1	1	14.05	163.1
100	1	1	1	N	0	1	1	1	-1	1	N	-1	0	-1	-35.65	88.22
101	1	1	1	1	1	1	0	-1	-1	1	1	0	0	1	-29.41	-51.15
102	1	1	1	1	0	1	1	1	0	-1	1	1	1	1	-113.5	-104.2
103	1	1	1	1	1	1	1	0	-1	1	1	-1	0	-1	-170.8	-93.09
104	1	1	1	N	1	1	0	1	-1	N	1	-1	1	-1	-249.6	-82.08
105	0	0	0	1	1	1	1	1	-1	N	1	-1	-1	1	-335.4	-263.3
106	1	1	1	1	0	1	0	1	-1	-1	1	-1	1	0	-24.38	-125.8
107	1	1	1	1	0	1	0	1	-1	-1	1	-1	1	-1	-239	-130.1
108	1	-1	-1	1	1	1	1	1	-1	-1	1	-1	0	1	-207.9	-108.5
109	1	-1	-1	1	1	-1	1	1	-1	-1	1	-1	1	1	-181.1	-98.77
110	1	-1	-1	N	1	1	1	1	-1	-1	1	-1	1	1	-198.5	-100.1
111	1	-1	-1	1	0	1	1	1	-1	-1	1	1	1	0	-224.6	-123.7
112	1	1	1	1	0	1	0	1	-1	-1	1	-1	-1	-1	-128.5	-78.66
113	1	1	1	1	0	1	1	0	-1	-1	N	-1	-1	-1	-222.9	-155.1

114	1	1	1	1	1	1	1	1	-1	-1	1	-1	-1	-1	-185.8	-165.2
115	1	1	1	1	0	1	0	1	1	-1	1	-1	1	0	-161.1	-144.6
116	1	1	1	1	1	1	0	-1	-1	1	1	-1	1	-1	-202.3	-99.21
117	1	1	1	1	0	0	0	-1	-1	-1	1	-1	1	1	-320.7	-157.5
118	1	-1	-1	-1	1	1	1	1	-1	1	-1	0	-1	-1	-111.4	-140.2
119	1	1	1	1	0	1	1	1	-1	N	1	-1	-1	-1	-36.99	1.879
120	1	1	1	1	0	1	1	1	-1	1	1	-1	0	-1	-126.8	-23.31
121	1	1	1	1	0	0	1	1	-1	-1	1	-1	1	-1	-15.42	-49.74
122	1	1	1	1	1	1	1	1	1	1	1	-1	-1	-1	-153.3	-90.96
123	1	1	1	1	0	1	1	1	-1	-1	1	-1	1	0	-58.26	-69.31
124	-1	1	1	1	0	-1	1	1	-1	-1	1	-1	-1	-1	-54.97	29.17
125	1	1	1	1	0	1	1	1	-1	-1	1	-1	-1	-1	69.46	112.2
126	1	1	1	1	1	1	1	1	-1	1	1	-1	-1	-1	-20.79	0.341
127	1	1	1	1	1	1	1	1	-1	N	1	-1	-1	-1	2.354	134.3
128	1	1	1	1	1	1	1	1	-1	-1	1	-1	-1	-1	14.33	111.1
129	1	1	1	1	0	1	1	1	-1	1	1	-1	-1	-1	-50.15	40.94
130	1	1	1	N	1	1	1	1	-1	N	N	1	0	-1	-38.3	13.87
131	1	1	1	1	0	1	1	1	-1	-1	1	-1	-1	-1	16.39	106.5
132	1	1	1	1	0	1	1	1	-1	-1	1	-1	-1	-1	-6.121	68.47
133	1	1	1	1	1	1	1	1	-1	-1	1	-1	-1	-1	27.31	106.1
134	1	1	1	1	1	1	1	1	-1	-1	1	-1	-1	-1	-59.93	-23.77
135	1	1	1	1	0	1	1	1	-1	-1	1	-1	-1	-1	-121.7	-123.2
136	1	1	1	1	1	1	1	1	-1	1	1	-1	-1	-1	-121.2	-45.92
137	1	1	1	1	0	1	1	1	-1	1	1	-1	-1	-1	-85.53	-56.17
138	1	1	1	1	0	1	1	1	-1	1	1	-1	-1	-1	-58.83	49.51
139	1	1	1	N	0	1	1	1	-1	-1	1	-1	-1	-1	-103.5	-51.8
140	1	1	1	1	0	1	1	1	-1	-1	1	-1	-1	-1	-119.7	-101.9
141	0	-1	-1	-1	0	1	1	1	-1	-1	1	-1	-1	-1	-20.26	-102
142	1	1	1	1	1	1	1	1	-1	1	1	-1	-1	-1	11.13	87.87

143	1	1	1	1	1	1	1	1	-1	1	-1	-1	-1	0	3.727	118.3
144	1	1	1	1	0	1	1	1	-1	N	1	-1	-1	-1	-7.331	3.795
145	1	1	1	1	0	1	1	1	-1	1	1	-1	-1	-1	-57.3	-75.9
146	1	1	1	1	0	1	1	1	-1	1	1	-1	-1	-1	-117.1	-103.4
147	1	1	1	1	0	1	1	1	1	-1	1	1	1	1	-57.77	-35.22
148	1	1	1	1	0	1	1	1	-1	1	1	-1	-1	-1	-24.03	47.39
149	1	1	1	1	0	1	1	1	-1	-1	1	-1	-1	-1	-64.05	-3.493
150	1	1	1	1	0	1	1	1	-1	-1	1	-1	-1	-1	-41.11	18.94
151	1	1	1	1	1	1	1	1	-1	-1	1	-1	-1	-1	-53.29	107.7
152	1	1	1	1	0	1	1	1	-1	-1	1	-1	-1	-1	-41.95	130.9
153	1	1	1	1	0	1	1	1	-1	1	1	-1	1	-1	-24.46	68.64
154	1	1	1	1	1	1	1	1	-1	1	1	-1	1	-1	50.01	123.4
155	1	1	1	1	0	1	1	1	-1	-1	1	1	-1	-1	28.56	10.24
156	1	1	1	1	1	1	1	1	-1	N	1	1	-1	-1	-22.34	-64.48
157	1	1	1	1	1	1	1	1	-1	-1	1	1	-1	-1	-113.5	-129
158	1	1	1	1	0	1	1	1	-1	-1	1	-1	-1	-1	-23.26	-39.67
159	1	1	1	N	0	1	0	1	-1	-1	1	-1	-1	-1	13.19	25.32
160	1	1	1	1	0	0	0	1	1	1	1	-1	-1	-1	-93.35	-6.244
161	1	1	1	1	-1	1	1	1	1	1	1	-1	-1	-1	-89.78	-122.6
162	1	1	1	1	0	1	0	1	1	1	1	1	1	1	-74.5	-90.21
163	1	1	1	1	-1	1	1	1	-1	-1	1	-1	1	1	-29.74	-126.3
164	1	1	1	1	0	1	1	1	0	-1	1	1	1	1	-94.43	-46.18
165	1	1	1	1	1	1	1	1	-1	-1	1	-1	-1	-1	-54.38	-42.69
166	1	1	1	1	0	1	0	1	-1	-1	1	-1	1	1	-99.9	-72.44
167	1	1	1	1	0	1	1	1	-1	N	1	1	1	1	-90.25	-77.52
168	1	1	1	1	1	1	1	1	-1	-1	1	1	1	1	69.14	168.5
169	-1	-1	-1	-1	1	1	1	1	-1	-1	1	1	1	1	13.41	39.02
170	1	1	1	1	0	1	0	1	-1	-1	1	1	1	1	-14.14	-14.62
171	1	1	1	1	0	1	0	1	-1	-1	1	1	1	1	-41.44	-57.2

172	1	1	1	N	-1	1	1	1	-1	1	1	1	1	1	16.7	-0.684
173	1	1	1	N	0	1	1	1	-1	1	1	1	1	1	-3.183	42.55
174	1	1	1	N	0	1	1	1	-1	N	N	1	1	1	0.652	90.28
175	1	1	1	1	0	1	1	1	-1	-1	1	1	1	1	-2.773	-26.53
176	1	1	1	1	-1	1	1	1	-1	-1	1	1	1	1	-6.854	69.79
177	1	1	1	1	0	1	1	1	-1	1	1	1	1	1	38.72	81.06
178	1	1	1	1	0	1	1	1	-1	1	1	1	1	1	-114.5	62.05
179	1	1	1	1	0	1	1	1	-1	-1	1	1	1	1	-17.47	-20.72
180	1	1	1	1	0	1	0	1	-1	-1	1	1	1	1	-49.79	-3.762
181	1	1	1	1	0	1	1	1	-1	-1	1	-1	1	1	-211.4	-206.3
182	1	1	1	N	1	1	1	1	-1	-1	1	1	1	1	-37.04	39.22
183	1	1	1	1	0	1	1	1	-1	N	N	1	-1	1	-45.97	85.4
184	1	1	1	1	-1	1	1	1	-1	-1	1	1	1	1	-16.14	2.423
185	1	1	1	1	0	-1	1	1	-1	1	1	-1	-1	-1	155	69.11
186	1	1	1	1	1	1	1	1	1	1	1	1	1	1	71	244.9
187	1	1	1	1	1	1	1	1	-1	-1	1	1	1	1	-45.11	-18.94
188	1	1	1	1	0	0	0	1	-1	-1	1	1	1	1	31.29	172.5
189	1	1	1	N	-1	1	0	1	0	N	1	1	1	1	112.2	NA
190	1	1	1	N	0	1	1	1	-1	N	1	-1	-1	1	123.2	346.2
191	1	1	1	1	0	1	1	1	-1	N	1	1	0	1	-62.51	-2.046
192	1	1	1	1	-1	1	1	1	-1	-1	1	0	0	1	177.6	-117.2
193	1	1	1	1	1	1	1	1	-1	-1	1	1	1	-1	74.61	192.2
194	1	1	1	1	1	1	-1	1	-1	1	1	1	1	1	280.3	NA
195	1	1	1	1	0	1	0	1	1	-1	1	1	1	1	18.48	67.56
196	1	1	1	1	0	1	0	1	-1	1	1	1	1	1	19.46	45.53
197	1	1	1	1	0	1	1	1	1	-1	1	1	1	1	0.706	149.5
198	1	1	1	1	0	1	1	1	0	1	1	1	1	1	321.1	NA
199	1	1	1	1	0	1	-1	1	-1	1	1	1	1	1	-17.99	82.31
200	1	1	1	1	1	1	-1	1	-1	1	1	1	1	1	333.5	NA

201	0	1	1	1	1	1	-1	1	1	-1	1	1	1	1	316.9	NA
202	0	1	1	1	1	1	0	1	1	-1	1	1	1	1	214.8	NA
203	1	1	1	1	0	1	1	1	1	-1	1	1	1	1	31.49	192.2
204	1	1	1	1	1	1	1	1	-1	-1	1	1	-1	-1	168.5	NA
205	0	1	1	1	0	1	-1	1	-1	-1	1	1	1	1	-96.87	-110.5
206	1	1	1	1	0	1	1	1	-1	1	-1	1	-1	-1	125	184.5
207	0	1	1	1	1	1	1	1	-1	1	1	1	1	1	463	NA
208	1	1	1	1	0	1	1	1	-1	-1	0	-1	1	1	368.9	-117.2
209	1	1	1	1	1	1	1	1	-1	1	1	1	-1	-1	74.66	NA
210	1	1	1	1	1	1	1	1	1	-1	1	1	1	1	133.3	NA
211	0	1	1	1	1	1	1	1	1	-1	1	1	1	1	404.1	NA
212	1	1	1	1	0	1	1	1	-1	0	1	1	-1	-1	53.46	143.3
213	1	1	1	1	0	1	1	1	-1	-1	1	1	1	1	147.6	89.31
214	1	1	1	1	0	1	1	1	-1	-1	1	1	0	1	224.6	NA
215	1	1	1	1	-1	1	1	1	-1	1	1	1	1	1	221.4	152.1
216	1	1	1	1	0	1	1	1	-1	-1	1	1	1	1	157	247.5
217	1	1	1	1	1	1	1	1	1	N	1	1	1	1	463	NA
218	0	1	1	1	1	1	0	1	1	-1	1	1	1	1	391	NA
219	1	1	1	1	0	1	1	1	1	-1	1	1	1	1	188.6	362.8
220	1	1	1	1	1	1	0	-1	-1	1	1	1	1	1	87.15	212.2
221	1	1	1	1	1	1	-1	-1	-1	1	1	1	1	1	100.1	NA
222	1	1	1	1	1	1	1	1	-1	-1	1	1	-1	-1	-114	-110.4
223	1	1	1	1	0	1	1	1	-1	-1	1	0	1	1	-6.503	17.45
224	1	1	1	1	0	1	1	1	-1	-1	1	1	1	1	-59.44	-128.8
225	1	1	1	1	0	1	1	0	0	1	1	1	1	1	86.96	152.2
226	1	1	1	1	1	1	0	-1	-1	1	N	1	1	1	-75.79	-30.99
227	1	1	1	1	1	1	-1	-1	-1	1	1	1	1	1	-69	-39.32
228	0	-1	-1	-1	0	1	1	1	-1	1	1	1	-1	-1	-233	-348.1
229	0	1	1	1	0	1	0	1	1	-1	1	1	1	1	521.6	NA

230	1	1	1	1	1	1	1	1	-1	1	1	1	1	1	463.1	NA
231	1	1	1	1	0	1	-1	1	-1	-1	-1	1	-1	-1	-153.7	-63.89
232	1	1	1	1	1	1	-1	1	1	-1	1	1	-1	-1	-233.5	-149.3
233	1	1	1	1	1	1	1	1	0	1	N	1	1	1	463	NA
234	0	1	1	1	0	1	-1	1	1	-1	1	1	-1	-1	-58.82	-93.52
235	1	1	1	1	1	1	0	1	1	1	1	1	1	1	214.7	NA
236	1	1	1	1	0	1	1	1	1	1	1	1	1	1	143.7	219.5
237	1	1	1	1	0	1	1	1	-1	1	1	1	1	1	152.3	NA
238	-1	1	1	1	1	1	-1	1	-1	1	1	1	1	1	99.68	NA
239	1	1	1	1	1	1	1	1	-1	1	-1	1	-1	-1	161.5	NA
240	1	1	1	1	1	1	1	1	1	1	1	1	1	1	127.4	300.5
241	1	1	1	1	1	-1	0	-1	-1	1	1	1	1	1	146.6	NA
242	1	1	1	1	1	1	1	0	-1	-1	1	1	1	1	70.99	156.4
243	-1	1	1	1	1	1	0	1	1	-1	1	1	1	1	-65.11	NA
244	1	1	1	1	-1	1	-1	1	1	-1	1	1	1	1	105.2	154.5
245	0	1	1	1	1	1	0	1	-1	1	1	1	1	1	294.9	NA
246	-1	1	1	1	1	1	1	1	-1	-1	-1	1	-1	-1	63.76	256.5
247	1	1	1	1	1	1	1	1	-1	-1	1	1	1	1	97.84	NA
248	1	1	1	1	-1	1	1	1	-1	1	1	1	1	1	159.8	175.6
249	1	1	1	1	0	1	1	1	1	-1	1	1	1	1	241.8	372.3
250	1	-1	-1	-1	1	1	0	1	-1	1	1	1	1	1	-79.49	-38.36
251	1	1	1	1	0	1	-1	1	-1	-1	-1	1	1	1	-0.553	16.23
252	1	1	1	1	0	N	1	1	1	-1	N	1	-1	-1	101.3	103
253	0	1	1	1	1	1	0	1	1	-1	1	1	1	1	245.7	NA
254	0	-1	-1	1	1	1	0	1	-1	1	-1	1	1	1	-128	-137.1
255	1	1	1	1	1	1	0	1	1	-1	1	1	1	1	19.1	154.6
256	-1	1	1	1	0	1	0	1	-1	-1	1	1	1	1	-18.94	106.7
257	1	1	1	1	-1	1	-1	1	-1	N	1	1	1	1	-66.55	-67.65
258	1	1	1	N	0	1	0	1	-1	N	1	1	1	1	87.56	NA

259	1	1	1	1	0	1	-1	1	-1	-1	1	1	1	1	171.9	332.7
260	1	1	1	1	0	1	1	1	-1	-1	1	1	1	1	100.8	NA
261	1	1	1	1	0	1	1	1	1	-1	1	1	1	1	51.1	177.2
262	1	1	1	1	1	1	0	1	1	-1	1	1	1	1	14.94	198.7
263	1	1	1	1	0	1	-1	1	-1	-1	1	1	1	1	129.6	NA
264	1	1	1	1	1	1	0	1	1	-1	1	1	1	1	89.05	218.7
265	1	1	1	1	0	1	-1	1	-1	1	1	1	1	1	70.88	372.6
266	1	1	1	1	1	1	-1	0	1	1	1	1	1	1	127.3	39.03
267	1	1	1	1	1	1	-1	1	1	-1	1	1	1	1	-57.6	81.44
268	0	1	1	1	1	1	0	1	1	-1	1	1	1	1	6.505	169.6
269	0	1	1	1	1	1	-1	-1	1	-1	1	1	1	1	2.373	96.02
270	0	1	1	N	0	1	0	1	-1	-1	1	1	1	-1	-80.3	-94.42
271	1	1	1	1	0	1	-1	1	-1	-1	1	1	1	1	-9.088	52.36
272	1	1	1	1	0	1	0	1	-1	-1	1	1	1	1	30.7	64.04
273	0	1	1	1	1	1	0	1	1	-1	1	1	1	1	236.6	NA
274	1	1	1	1	1	-1	-1	1	-1	1	1	1	1	1	79.27	109.9
275	1	1	1	1	1	1	-1	-1	-1	1	1	1	1	1	158.5	NA
276	1	1	1	1	1	1	1	1	-1	-1	1	1	1	1	262	NA
277	1	1	1	1	0	1	1	1	-1	1	1	1	1	1	85.69	NA
278	0	1	1	1	0	1	0	1	1	1	1	1	-1	-1	-92.75	-110.4
279	1	1	1	1	0	1	1	1	1	-1	1	1	1	1	163.2	253
280	1	1	1	1	0	1	0	1	-1	N	1	1	1	1	294.3	NA
281	1	1	1	N	1	1	0	1	1	-1	1	0	1	1	236.5	NA
282	1	1	1	1	1	1	1	1	-1	1	-1	1	-1	-1	11.46	NA
283	1	1	1	1	-1	1	-1	1	-1	-1	1	1	1	1	235.3	291.4
284	1	1	1	1	1	1	0	1	1	-1	1	1	1	1	74.75	21.92
285	1	1	1	1	1	1	-1	1	-1	-1	1	1	1	1	29.24	161.7
286	1	1	1	1	1	1	0	1	-1	1	1	1	1	1	90.25	NA
287	1	1	1	1	0	1	-1	1	1	-1	1	1	1	1	111.5	159.5

288	1	1	1	1	-1	1	1	1	1	-1	1	1	1	1	232.7	372.3
